# Guidelines for Neuroprognostication in Critically ill Adults with Acute Ischemic Stroke

**DOI:** 10.1007/s12028-026-02486-3

**Published:** 2026-04-06

**Authors:** Shraddha Mainali, Gabriel V. Fontaine, Venkatakrishna Rajajee, Felipe A. Montellano, Susanne Muehlschlegel, Katja E. Wartenberg, Sheila A. Alexander, Katharina M. Busl, Sara E. Hocker, David Y. Hwang, Keri S. Kim, Dominik Madzar, Dea Mahanes, Juergen Meixensberger, Oliver W. Sakowitz, Panayiotis N. Varelas, Christian Weimar, Thomas Westermaier, Claire J. Creutzfeldt

**Affiliations:** 1 Department of Neurology, Virginia Commonwealth University, Richmond, VA USA; 2 Departments of Pharmacy and Neurosciences, Intermountain Health, Salt Lake City, UT USA; 3 Departments of Neurology and Neurosurgery, University of Michigan, Ann Arbor, MI USA; 4 Department of Neurology, Institute for Clinical Epidemiology and Biometry, University Hospital Würzburg (UKW), Würzburg, Germany; 5 Department of Neurology, Department of Anesthesiology/Critical Care Medicine, and Department of Neurosurgery, Neurosciences Critical Care Division, Johns Hopkins School of Medicine, Baltimore, MD USA; 6 Department of Neurology, University of Leipzig, Leipzig, Germany; 7 School of Nursing, University of Pittsburgh, Pittsburgh, PA USA; 8 Departments of Neurology and Neurosurgery, College of Medicine, University of Florida, Gainesville, FL USA; 9 Department of Neurology, Mayo Clinic, Rochester, MN USA; 10 Department of Neurology, University of North Carolina at Chapel Hill, Chapel Hill, NC USA; 11 Pharmacy Practice, University of Illinois, Chicago, IL USA; 12 Department of Neurology, University of Erlangen, Erlangen, Germany; 13 Departments of Neurology and Neurosurgery, University of Virginia Health, Charlottesville, VA USA; 14 Department of Neurosurgery, University of Leipzig, Leipzig, Germany; 15 Department of Neurosurgery, Neurosurgery Center Ludwigsburg-Heilbronn, Ludwigsburg, Germany; 16 Department of Neurology, Albany Medical College, Albany, NY USA; 17 Institute of Medical Informatics, Biometry, and Epidemiology, University Hospital Essen, Essen, Germany; 18 BDH-Clinic Elzach, Elzach, Germany; 19 Department of Neurosurgery, University of Wurzburg, Würzburg, Germany; 20 Department of Neurology, University of Washington, Seattle, WA USA

**Keywords:** Ischemic stroke, Neurocritical care, Prognosis, Prognostication, Outcome

## Abstract

**Background:**

Patients with acute ischemic stroke (AIS) may require intensive care unit admission for several reasons, including post-procedural care, management of large hemispheric infarction, and cardiopulmonary instability. The objective of this document is to provide recommendations on the reliability of select individual predictors of outcome, and multivariate prediction models, in the context of counseling critically ill patients with AIS and their surrogates. In addition, broad principles of neuroprognostication in this population were identified.

**Methods:**

A narrative systematic review was completed using Grading of Recommendations Assessment, Development and Evaluation (GRADE) methodology. Candidate predictors, including clinical variables and prediction models, were selected on the basis of clinical relevance and the presence of an appropriate body of evidence. The Population/Intervention/Comparator/Outcome/Timing/Setting (PICOTS) question was framed as follows: “When counseling critically ill adults with AIS or their surrogates, should <predictor> be considered a reliable predictor of poor functional outcome at 3 months or later?” Recommendations were based on quality of evidence, balance of desirable and undesirable consequences, values and preferences, and resource use. Recommendations that met GRADE criteria for good practice statements addressed broad principles of neuroprognostication.

**Results:**

A total of 518 articles met eligibility criteria to guide recommendations. Good practice recommendations include avoiding premature neuroprognostication, avoiding confounders, the use of multimodal assessment, predicting recovery of swallow function, predicting tracheostomy decannulation, and counseling of patients with large hemispheric infarction and their surrogates prior to neurological decline. Early neurological improvement within 24 h of revascularization was identified as a moderately reliable predictor of good functional outcome. No other individual predictor was considered reliable for the prediction of mortality or functional outcome.

**Conclusions:**

These guidelines suggest broad principles of neuroprognostication and provide recommendations on the reliability of predictors of functional outcome in the context of counseling critically ill patients with AIS and their surrogates.

**Supplementary Information:**

The online version contains supplementary material available at 10.1007/s12028-026-02486-3.

## Introduction

Each year approximately 795,000 people in the USA experience a new or recurrent stroke, of which about 87% are ischemic [1]. Stroke ranks as the second-leading cause of death worldwide and the third-leading cause of both death and disability combined [2]. Approximately 10–20% of patients with acute ischemic stroke (AIS) will be admitted to an intensive care unit (ICU) [3, 4]. Reasons for admission to the ICU include disorders of consciousness, cerebral edema, cardiopulmonary complications, and procedural complications [5]. Patients may also be admitted to the ICU following reperfusion therapy. The mortality of patients with AIS admitted to the ICU is approximately 20–40%, but is highly variable overall and likely depends on the criteria for ICU admission [4–8]. Similarly, depending on the criteria for ICU admission, approximately a fifth to approximately one half of all patients with AIS admitted to the ICU will achieve long-term functional independence [4, 5, 9–11].

Patients with severe stroke are often unable to participate in life-or-death treatment decisions, leaving surrogates to make decisions on escalation or continuation of life sustaining therapy. Most people who die early after stroke do so after a patient/surrogate decision to withhold or withdraw life-sustaining treatment (WLST) [12–14]. Most patients with AIS with compromised consciousness who die do so following WLST [15]. Decisions to withhold or withdraw life sustaining treatment are typically preceded by discussions with clinicians who provide a best estimate of neurological prognosis. While such decisions are often entirely appropriate, and in accordance with the patient’s preferences, prognostication in critically ill patients with AIS is highly challenging given that the stakes are typically high. The risk of premature WLST in a patient who may have recovered to functional independence must be balanced against the risk of prolonging the suffering of individuals with poor quality of life [16]. While prognostication in critically ill patients after ischemic stroke is often focused on the possibility of a *poor* outcome, prediction of a likely *good* outcome is also meaningful to patients and surrogates. Clinicians are fortunately faced with the latter situation more often than ever before, with the availability of effective revascularization strategies such as mechanical thrombectomy, and the expansion in eligibility of patients to receive these treatments [17].

Since neuroprognostication following AIS is highly consequential it is critical that clinicians base their assessments on reliable, evidence-based predictors of outcome. Clinical practice varies greatly, however, around multiple aspects of prognostication following acute ischemic stroke: timing, predictors used to formulate a prognosis, language during counseling, and disclaimers of uncertainty. There is an urgent need for guidance on best practices, and on the reliability of predictors clinicians commonly use to formulate a prognosis. The specific objectives of these Neurocritical Care Society (NCS) and Deutsche Gesellschaft für Neurointensivmedizin (DGNI, German Society for Neuro-intensive and Emergency Medicine) guidelines were to provide broad principles of neuroprognostication, and recommendations on the reliability of individual clinical variables and multivariate prediction models used by clinicians to formulate a prognosis in the context of counseling critically ill patients with AIS and their surrogates.

### Scope of the Guidelines

The scope of these Grading of Recommendations Assessment, Development, and Evaluation (GRADE) guidelines is the prognostication of neurological outcome in adults with AIS who have received standard-of-care treatment. Standard of care treatment includes, but is not limited to, revascularization therapy in appropriately selected individuals. The primary focus of this document is on patients who require intensive care unit (ICU) admission for management of the stroke itself, including post-procedural care following revascularization therapy. These guidelines are therefore not focused on patients with mild ischemic stroke or transient ischemic attack, who typically do not require ICU admission, and whose prognosis for long-term neurological recovery from the index event is generally good. The recommendations in these guidelines are focused on early neuroprognostication, particularly in the ICU. Patients admitted to inpatient rehabilitation units, or otherwise receiving post-discharge rehabilitation services, are outside the scope of this document.

### How to Use These Guidelines

Individual predictors of outcome and multivariate prediction models were categorized as *reliable*, *moderately reliable*, or *not reliable* (Table 1). This was based on a GRADE-based assessment of certainty in the body of evidence, as well as effect size (quantification of predictor accuracy) across published studies. The degree of uncertainty that is conveyed while counseling surrogates is tailored to the reliability of the predictors used during prognostication.

**Table 1:** Reliable and moderately reliable predictors

Category of predictor/model	GRADE criteria					Point estimates of accuracy in the body of evidence	Use during counseling of patients or surrogates?	Presence of additional specific reliable or moderately reliable predictors required for use during counseling?	Suggested language during counseling of patients or surrogates	
	Risk of bias	Inconsistency	Imprecision	Indirectness	Quality of evidence: overall				Likelihood of outcome	Disclaimer of uncertainty during counseling
Reliable	One downgrade permitted	Downgrade NOT permitted	Downgrade NOT permitted	Downgrade NOT permitted	Moderate or high	Individual predictors require false positive rate (FPR) ≤ 3% (upper limit 95% confidence interval < 10%). Prediction models require AUC > 0.8, no evidence of miscalibration in external validation studies	Yes	Preferred, but not absolutely required	“Very likely”	Present, but low
Moderately reliable	One downgrade permitted	Downgrade NOT permitted	One downgrade permitted	One downgrade permitted	Any	Individual predictors of poor outcome require FPR ≤ 3% (imprecision allowed). Predictors of good outcome require positive predictive value > 60%	Yes	Yes	“Likely”	Substantial
Moderately reliable clinical prediction models	One downgrade permitted	Downgrade NOT permitted	One downgrade permitted	One downgrade permitted	Any	AUC > 0.7, some miscalibration allowed in external validation studies	Yes	No	Use predicted probability of outcome	“The predicted probability is an estimate, subject to considerable uncertainty”
Not reliable	Downgrade permitted	Downgrade permitted	Downgrade permitted	Downgrade permitted	Any	Any	*No	Not applicable	Not applicable	Not applicable

*Many predictors designated “not reliable” are practically utilized by clinicians in formulating and communicating real-world subjective impressions of prognosis. The purpose of these guidelines is to identify predictors, if any, that meet reliable or moderately reliable criteria

A key distinction exists between a *reliable* predictor of outcome in the context of counseling surrogates of patients requiring life-sustaining therapy and an *independent* predictor of outcome. An *independent* predictor fulfills one criterion—a statistically significant association with the outcome of interest in an appropriately conducted multivariable analysis. In clinical practice, independent predictors of outcome may be used in risk stratification, selection of patients for targeted treatment (such as chemotherapy regimens for cancer), or as building blocks of clinical prediction models. A *reliable* predictor in the context of counseling surrogates of patients requiring life-sustaining therapy must be independent, but also fulfill other criteria as described in the “Evidence to Recommendation (EToR)” section. Confidence in the accuracy of the predictor should be sufficiently high to overcome concerns about the undesirable consequence of inappropriate withdrawal of life-sustaining therapy.

These guidelines were structured to align with the principles of ethical clinical practice. Non-maleficence was prioritized, to avoid patient harm from the self-fulfilling prophecy and inaccurate predictions that lead to inappropriate WLST.

## Methods

An in-depth description of the methodology used in these guidelines is available in Supplementary Appendix 1.

### Selection of Candidate Predictors

The following candidate predictors were selected on the basis of clinical relevance AND the presence of an appropriate body of literature, using criteria described in Supplementary Appendix 1:

#### Clinical Variables

1. Age
2. National Institutes of Health Stroke Scale (NIHSS) on admission
3. Blood glucose
4. Cerebral collateral circulation status
5. Hypertension
6. Infarct size
7. History of previous stroke
8. Revascularization status
9. Early neurological improvement (ENI)

#### Clinical Prediction Models

1. Acute Stroke Registry and Analysis of Lausanne (ASTRAL) score
2. Dense Artery, mRS, Age, Glucose, Onset-to-Treatment, and NIHSS (DRAGON) score
3. Ischemic Stroke Predictive Risk Score (iScore)
4. Totaled Health Risks in Vascular Events (THRIVE) score

### PICOTS Question

The Population/Intervention/Comparator/Outcome/Time frame/Setting (PICOTS) question was then framed for the specific candidate predictors as follows:

“When counseling critically ill adults with acute ischemic stroke or their surrogates, should <predictor or prediction model, with time of assessment if appropriate> be considered a reliable predictor of <outcome>?”

### Selection of Outcomes

The topic experts and the patient and family representative rated outcomes on the GRADE 1–9 scale on the basis of their perceived importance. Outcomes with a median rating of 7–9 were considered “critical.” While quality of life, cognitive function, and depression were critical outcomes, the body of evidence with these outcome measures was insufficient to support any recommendations. In addition, the risk of bias from the self-fulfilling prophecy within the existing body of literature on predictors of mortality was considered too high to recommend any single clinical variable to predict death *when all available means of life support are used, indefinitely and without limitation* [18]. A brief summary of the GRADE evidence profile and summary of findings for predictors of mortality is given in Supplementary Table 1, while a summary of recommendations for predictors of mortality is provided in Supplementary Table 2. The recommendations within these guidelines are primarily focused on the prediction of functional outcome.

### Functional Outcome Assessment

The majority of studies used the modified Rankin Scale (mRS), which ranges from 0 (asymptomatic) to 6 (death), as a measure of functional outcome [19, 20]. The definition of a “good” or “poor” functional outcome has varied across the literature. In recognition of the evolution in outcome analysis in the ischemic stroke literature (described in Supplementary Appendix 1), an inclusive definition was used for the purposes of this systematic review, encompassing all of the mRS thresholds and analyses described. Variability in outcome definition was, therefore, an inherent limitation of the review. Other acceptable measures of functional outcome in the literature included the Barthel Index, Glasgow Outcome Scale (GOS), Functional Independence Measure (FIM), and Lawton’s Activities of Daily Living (ADL) scale.

### Systematic Review Methodology

**Fig. 1:**
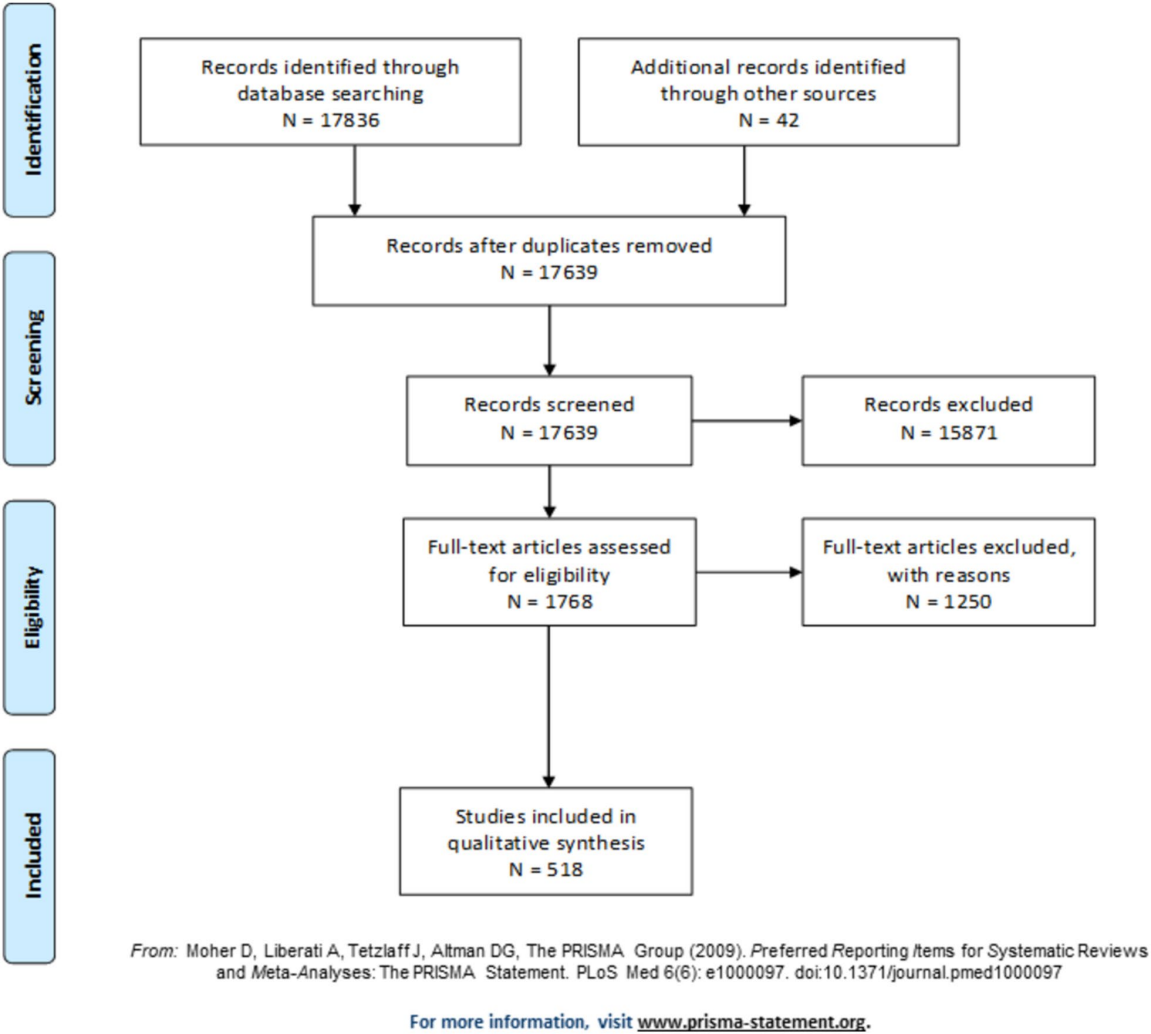
PRISMA 2009 flow diagram depicting the study selection process for the systematic review, including records identified, screened, excluded, and included in the final analysis

Since the literature on prognosis was expected to be heterogeneous, a narrative systematic review was performed. An in-depth description of systematic review methodology overall for the NCS–DGNI neuroprognostication guidelines project is given in Supplementary Appendix 1. The initial librarian search string was appropriate to the question “What are the reliable predictors (prognostic factors, variables, tests, scores, and multivariable models), prior to and on admission as well as during the hospital course to predict patient outcome at different follow-up time-points following each specific disease ?”, to identify candidate predictors. Screening of articles was completed using DistillerSR software (Evidence Partners, Ottawa, Canada). The librarian search string used for this systematic review is provided in Supplementary Appendix 2. The Preferred Reporting Items for Systematic Reviews and Meta-Analyses (PRISMA) flow diagram is shown in Fig. 1. The initial librarian search was performed on 20 February 2019 and encompassed the period from 1946 to the search date. Databases searched included MEDLINE via PubMed, EMBASE, Web of Science, and the Cochrane Database of Systematic Reviews. Updated searches were performed on 1 August 2022 and 5 February 2024.

### Article Screening

Following title and abstract screening, full-text screening was performed with the following exclusion criteria: (1) sample size less than 100 adult patients, (2) studies focused exclusively on transient ischemic attack (TIA) and/or mild stroke, (3) studies focused on a highly selected subgroup (such as periprocedural stroke), (4) studies of predictors not evaluated in multivariate analysis, (5) studies focused on a genetic polymorphism as a predictor, and (6) studies of clinical prediction models that did not report model discrimination.

### Risk of Bias (RoB) Assessment

Data extraction and assessment for risk of bias (RoB) was performed for studies that addressed the PICOTS question and fulfilled full-text selection criteria. The Quality in Prognostic Studies (QUIPS) RoB instrument was used to evaluate studies of individual prognostic variables [21]. The Prediction model Risk Of Bias ASsessment Tool (PROBAST) instrument was used to evaluate studies of clinical prediction models [22, 23]. In addition to the standard domains of these RoB instruments, studies were evaluated for the risk of bias related to the self-filling prophecy with an additional domain that included three questions: whether a treatment suspension policy was used in the study, whether clinicians were blinded to the predictor, and whether the predictor was systematically utilized by clinicians for prognostication during the time period of the study. A content expert evaluated each study for RoB, with secondary review by another content expert. Articles written or co-written by one of the authors were independently assessed for risk of bias by another author and a methodologist. Following assessment of risk of bias in each domain, an overall risk of bias—*high, moderate, or low*—was assigned to each study.

### GRADE Assessment and Evidence Profile

Following data extraction and assessment of risk of bias of individual studies, a GRADE evidence profile with summary of findings table was constructed. The body of evidence was downgraded once for a moderate overall RoB across all individual studies and twice for a high overall RoB. Since most studies of predictors following AIS were not limited to critically ill patients admitted to the ICU, a downgrade for indirectness was required for the body of evidence for most PICOTS questions.

A summary of individual studies is provided in Supplementary Table 3. The GRADE evidence profile and summary of findings table for predictors of functional outcome are presented in Table 2. A summary of PICO recommendations is given in Table 3, while a color-coded summary of reliability of predictors is presented in Table 4. A summary of good practice statements is given in Table 5.

**Table 2:** GRADE evidence profile/summary of findings table: neuroprognostication—acute ischemic stroke

Outcome	Predictor	Quality of evidence					Summary of findings (narrative of effect size)
		Risk of bias	Inconsistency	Indirectness	Imprecision	Quality of evidence: summary	
*Individual clinical variables*							
Poor functional outcome	Age	↓	↓	↓		Very low	OR: 0.74–5.25
Poor functional outcome	NIHSS	↓	↓	↓	↓	Very low	OR: 0.53–20.1
Poor functional outcome	Blood glucose	↓	↓	↓	↓	Very low	OR: 1.021–5.23
Poor functional outcome	Cerebral collateral circulation status	↓			↓	Low	OR: 0.21–14.13
Poor functional outcome	Hemorrhagic transformation (HT)	↓↓	↓	↓	↓	Very low	OR: 0.96–3.23
Poor functional outcome	Infarct size	↓↓	↓	↓	↓	Very low	OR: 0.57–9.15
Poor functional outcome	Previous stroke	↓↓	↓	↓	↓	Very Low	OR: 1.23–4.05
Good functional outcome	Revascularization	↓			↓	Low	OR: 1.69–34.08
Good functional outcome	Early neurological improvement (ENI)	↓			↓	Low	OR: 1.27–22.6; 61–84% of patients with ENI return to functional independence
*Clinical prediction models*							
Poor functional outcome	ASTRAL	↓	↓			Low	*C*-stat: 0.73–0.94
Poor functional outcome	DRAGON	↓↓	↓			Very low	*C*-stat: 0.73–0.85
Poor functional outcome	iScore	↓↓	↓			Very low	*C*-stat: 0.68–0.86
Good functional outcome	THRIVE	↓↓				Low	*C*-stat: 0.65–0.85

**Table 3:** Recommendations: guidelines for neuroprognostication in acute ischemic stroke

	Recommendations: Clinical variables as predictors of functional outcome
1	When counseling critically ill adults with AIS or their surrogates, we suggest age alone not be considered a reliable predictor of poor functional outcome assessed at 3 months or later *(weak recommendation; very low quality evidence)*
2	When counseling critically ill adults with AIS or their surrogates, we suggest the admission National Institutes of Health Stroke Scale (NIHSS) alone not be considered a reliable predictor of poor functional outcome assessed at 3 months or later *(weak recommendation; very low quality evidence)*
3	When counseling critically ill patients with AIS or their surrogates, we suggest hemorrhagic transformation alone not be considered a reliable predictor of poor functional outcome assessed at 3 months or later *(weak recommendation; very low quality evidence)*
4	When counseling critically ill patients with AIS or their surrogates, we suggest infarct size alone not be considered a reliable predictor of poor functional outcome assessed at 3 months or later *(weak recommendation; very low quality evidence)*
5	When counseling of critically ill patients with AIS or their surrogates, we suggest a history of previous stroke alone not be considered a reliable predictor of poor functional outcome assessed at 3 months or later *(weak recommendation; very low quality evidence)*
6	When counseling critically ill patients with AIS or their surrogates, we suggest successful revascularization alone not be considered a reliable predictor of good functional outcome assessed at 3 months or later *(weak recommendation; moderate quality evidence)*
7	When counseling critically ill patients with AIS or their surrogates, we suggest early neurological improvement (ENI) assessed 24 h following revascularization, or 24 h following admission for patients who are not candidates for revascularization, be considered a moderately reliable predictor of good functional outcome assessed at 3 months or later. A good outcome is expected in patients with recovery to an NIHSS of 0 to 1 at 24 h *(weak recommendation; low quality evidence)*
8	When counseling critically ill patients with AIS or their surrogates, we suggest admission hyperglycemia alone not be considered a reliable predictor of poor functional outcome assessed at 3 months or later *(weak recommendation; very low quality evidence)*
9	When counseling critically ill patients with AIS or their surrogates, we suggest cerebral collateral circulation status alone not be considered a reliable predictor of poor functional outcome assessed at 3 months or later *(weak recommendation; very low quality evidence)*
	Recommendations: Clinical prediction models as predictors of functional outcome
10	When counseling critically ill patients with AIS or their surrogates, we suggest the Acute Stroke Registry and Analysis of Lausanne (ASTRAL) score not be considered a reliable predictor of poor functional outcome assessed at 3 months or later *(weak recommendation; moderate quality evidence)*
11	When counseling critically ill patients with AIS or their surrogates, we suggest the Dense Artery, mRS, Age, Glucose, Onset-to-Treatment, and NIHSS (DRAGON), MR-DRAGON, and MT-DRAGON scores not be considered reliable predictors of poor functional outcome assessed at 3 months or later *(weak recommendation; very low quality evidence)*
12	When counseling critically ill patients with AIS or their surrogates, we suggest the Ischemic Stroke Predictive Risk Score (iScore) prediction model not be considered a reliable predictor of poor functional outcome assessed at 3 months or later *(weak recommendation; moderate quality evidence)*
13	When counseling critically ill patients with AIS or their surrogates, we suggest the Totaled Health Risks in Vascular Events (THRIVE), THRIVE-c, and THRIVE-EVT scores not be considered reliable predictors of good functional outcome assessed at 3 months or later *(weak recommendation; low quality evidence)*

**Table 4:** Reliability of predictors of functional outcome

Predictor	Reliability for prediction of functional outcome at 3 months or later
Clinical variables	
Age	Not reliable
NIHSS	Not reliable
Blood glucose	Not reliable
Cerebral collateral circulation status	Not reliable
Infarct size	Not reliable
Hemorrhagic transformation	Not reliable
Previous stroke	Not reliable
Revascularization status	Not reliable
Early neurological improvement	Moderately reliable
Clinical prediction models	
ASTRAL	Not reliable
DRAGON/MR-DRAGON/MT-DRAGON	Not reliable
iScore	Not reliable
THRIVE	Not reliable

Reliability of predictors of functional outcome at 3 months or later. Recommendations address prediction of good functional outcome for revascularization status, early neurological improvement, and THRIVE. Other recommendations address prediction of poor functional outcome. NIHSS, National Institutes of Health Stroke Scale; ASTRAL, Acute Stroke Registry and Analysis of Lausanne; DRAGON, Dense Artery, mRS, Age, Glucose, Onset-to-Treatment, and NIHSS; iScore, Ischemic Stroke Predictive Risk Score; THRIVE, Totaled Health Risks in Vascular Events

**Table 5:** Good practice statements

1	We recommend that prognostication be performed with consideration of the complete clinical condition. (Strong recommendation, evidence cannot be graded)
2	We recommend that the assessment of neurological prognosis be performed in the absence of potential confounders of the neurological exam. (Strong recommendation, evidence cannot be graded)
3	We recommend that preexisting functional impairment and comorbidities be considered when formulating a prognosis. (Strong recommendation, evidence cannot be graded)
4	We recommend that counseling of critically ill patients with AIS expected to survive the index hospital admission, and/or their surrogates, include discussion of long-term functional outcomes. Discussion should not focus on day-to-day changes and in-hospital outcomes alone. (Strong recommendation, evidence cannot be graded)
5	We recommend that acceptable outcomes for individual patients serve as the basis for prognostication and shared decision-making. (Strong recommendation, evidence cannot be graded)
6	We recommend avoiding premature neuroprognostication in critically ill patients with AIS, including patients with severe ischemic stroke. While early neurological improvement (ENI) following revascularization may clarify a likely good outcome at 24 h, the appropriate period of observation in an individual patient should be determined with careful consideration of factors such as the anticipated evolution of neurological illness, potential confounders of the neurological exam, and completion of appropriate diagnostic imaging. (Strong recommendation, evidence cannot be graded)
7	We recommend that critically ill patients with *severe* AIS, and/or their surrogates, be counseled that incremental functional recovery may occur up to 6 months, and sometimes longer, following the stroke whereas most patients with AIS of mild to moderate severity will achieve the majority of their functional recovery within 3 months. (Strong recommendation, evidence cannot be graded)
8	We recommend that critically ill patients with AIS who require artificial enteral nutrition, and/or their surrogates, be counseled that recovery of swallow function and removal of the feeding tube is likely, but not certain, within a period of several months among survivors. (Strong recommendation, evidence cannot be graded)
9	We recommend that critically ill patients with AIS who require tracheostomy, and/or their surrogates, be counseled that successful decannulation is possible within a period of several months among survivors. (Strong recommendation, evidence cannot be graded)
10	We recommend that discussions of prognosis and goals of care for patients with large hemispheric infarction occur prior to neurological deterioration where feasible, to allow patients and surrogates sufficient time to make informed decisions on decompressive craniectomy and other therapeutic interventions. (Strong recommendation, evidence cannot be graded)

### Evidence to Recommendation Criteria

1. Quality of evidence/certainty in the evidence and effect size: For the purposes of these guidelines, predictors described as “reliable” have both a higher overall certainty in the evidence and greater effect size than “moderately reliable” predictors (Table 1). The acceptable threshold for predictors of good outcome was considerably lower than for predictors of poor outcome, since an inappropriate prediction of good outcome is not expected to lead to a potentially irreversible decision such as WLST.
2. Balance of desirable and undesirable consequences: An accurate prediction of poor outcome is expected to result in grief, a sense of loss and anxiety about the future. However, accurate prediction of a poor outcome is overall desirable, since it allows surrogates and the clinical team to align goals of care to the perceived wishes of the patient with AIS, through the process of shared decision-making. Potential benefits to the family and surrogates in this situation include greater certainty and decreased decisional conflict in making patient value-congruent decisions, a sense of closure, and satisfaction from respecting the patient’s wishes. An *inaccurate* prediction of a poor outcome (i.e., a false-positive prediction of poor outcome), however, may lead to WLST in a patient with AIS who would otherwise have made a recovery to functional independence. Since WLST almost always leads to death in critically ill patients on life-sustaining therapy, the undesirable consequences of an inaccurate prediction of poor outcome were thought to greatly outweigh the desirable consequences, unless certainty in the accuracy of the predictor or prediction model was high. *The concern for inappropriate WLST was central in the panel’s decision to recommend, or not recommend, prognostication and counseling on the basis of the selected predictors of poor outcome*. The prediction of *good* outcome, however, involves a different balance of desirable and undesirable consequences. Patients and surrogates are likely to feel encouraged and hopeful, and continue life-sustaining treatment, which was overall considered desirable. Anticipation of a good long-term outcome may ease the psychological and physical burdens of critical illness and short-term debility, for both patients and surrogates. Anticipation of a good long-term outcome was also thought to be desirable for patient motivation during rehabilitation, which may be an independent predictor of good functional outcome following AIS [24]. An undesirable consequence of inaccurate prediction of a good outcome is disappointment and disillusionment among both patients and surrogates, which may adversely impact motivation. An inaccurate prediction of good outcome may also lead to prolongation of suffering among patients and surrogates. An additional undesirable consequence was thought to be discordance in the understanding of “good” outcome between clinicians and patients/surrogates. While clinicians generally consider a good outcome following AIS to be a return to functional independence, patients and surrogates may perceive psychological or cognitive impairment, and limitations in employment or leisure activities to represent a poor outcome despite a return to functional independence. Conversely, a physician’s perceived poor outcome might be seen by patients and families as acceptable [25–27]. Overall, the panel considered a prediction of good outcome to be desirable, using language commensurate to the actual likelihood of recovery, such as “more likely/more often than not,” or “highly likely, but not certain.” Clarity during counseling regarding the specific “good” outcome under discussion, such as recovery of ambulation, recovery of swallow function, or the ability to perform basic activities of daily living, was also thought to be important.
3. Values and preferences: The panel agreed that most individuals, as well as their families and surrogates, would consider an inaccurate prediction of poor outcome that led to the death of a patient who might otherwise have had a reasonable recovery to be more undesirable than a prolonged period of uncertainty in the outcome. Therefore, a high certainty in the evidence of predictor or prediction model accuracy was necessary to recommend consideration when counseling families and surrogates on prognosis in this context. Patient and family representatives on the panel, however, stated a preference for the clinical team communicating any expectations of recovery of function.
4. Resource use: An important consideration was the substantial financial burden that may be imposed on patients, surrogates, and the healthcare system with continuation of supportive measures based on an inaccurate prediction of good outcome—particularly when the patient’s outcome is inconsistent with their desired quality of life [28–30]. An accurate prediction of *poor* outcome will lead to better alignment of goals of care with the patient's wishes and avoid the extended use of resources, over days to years. The use of resources was therefore thought to favor consideration of a predictor or prediction model during prognostication, contingent on confidence in its predictive accuracy.

### Recommendations: Clinical Variables as Predictors of Functional Outcome

*PICO question 1*: “When counseling critically ill adults with AIS or their surrogates, should age alone be considered a reliable predictor of poor functional outcome assessed at 3 months or later?”.

*Description of the predictor*: Older age may reflect underlying frailty, the presence of comorbid conditions, and diminished cognitive reserve [31].

*Recommendation*: When counseling critically ill adults with AIS or their surrogates, we suggest age alone not be considered a reliable predictor of poor functional outcome assessed at 3 months or later *(weak recommendation; very low quality evidence).*

*Rationale*: The body of evidence was downgraded once each for inconsistency, indirectness, and risk of bias in the domain of study participation, attrition, outcome measurement, confounding, statistical analysis, and self-fulfilling prophecy. Despite the presence of some inconsistency, older age was a strong and independent predictor of poor functional outcome in most studies. However, age in isolation could not be recommended as a reliable predictor because older patients with AIS may nevertheless have a good functional outcome because of factors such as lower stroke severity or successful revascularization. Premorbid health and functional status varies among the elderly and may impact individual outcomes. An age threshold that universally predicts poor outcome has not been identified.

While several studies have shown that patients older than 80 years are more likely to have a poor outcome than younger patients following thrombectomy [32–34], data from clinical trials suggest that patients older than 85 years do not universally suffer a poor outcome. In the HERMES meta-analysis of clinical trials, patients > 85 years *with premorbid functional independence* treated with thrombectomy were more likely to regain functional independence (seen in 18% of patients) than control subjects [35]. Among patients 80 years and older who underwent thrombectomy, 30% regained functional independence (mRS 0–2) and 49% achieved independent ambulation (mRS 0–3) [36]. Data from clinical trials do not necessarily reflect real-world outcomes, however, since trial eligibility criteria typically select patients most likely to demonstrate benefit from the intervention. When counseling patients older than 85 years who have undergone thrombectomy, or their surrogates, we therefore suggest that statistics from clinical trials be considered a best-case estimate, and the entire range of possible outcomes be presented.

*PICO question 2*: “When counseling family members and/or surrogates of patients with AIS admitted to an ICU, should the admission NIHSS alone be considered a reliable predictor of poor functional outcome assessed at 3 months or later?”.

*Description of the predictor*: While several scales exist, the National Institutes of Health Stroke Scale (NIHSS), a 15-item scale first described in 1989 [37], was selected as the predictor in this PICO question since it is the best validated and most widely used. NIHSS was often included as a continuous variable; however, dichotomization or categorization (e.g., mild, moderate, severe) was also reported in included studies. NIHSS > 15 typically represents a severe stroke [38, 39]. Of note, the NIHSS was initially designed for use in clinical trials rather than bedside assessment, or prognostication [40].

*Recommendation*: When counseling critically ill adults with AIS or their surrogates, we suggest the NIHSS alone not be considered a reliable predictor of poor functional outcome assessed at 3 months or later *(weak recommendation; very low quality evidence).*

*Rationale*: The body of evidence was downgraded once each for inconsistency, indirectness, imprecision, and risk of bias in the domains of study participation, attrition, measurement of prognostic factors and outcomes, control of confounding variables, statistical analysis methods, and the self-fulfilling prophecy. Despite the presence of some inconsistency, admission NIHSS was a strong and independent predictor of poor functional outcome in most studies. However, the NIHSS in isolation could not be recommended as a reliable predictor in individual patients for several reasons. The NIHSS was measured at first assessment, prior to revascularization therapy, in most studies. Since revascularization is an effective treatment that may result in dramatic improvement regardless of baseline stroke severity, the NIHSS measured prior to therapeutic intervention is of limited prognostic value during counseling [41]. For example, in the HERMES meta-analysis, 32% of patients with the greatest stroke severity (NIHSS > 20) who underwent thrombectomy were independent (mRS 0–2) at 90 days, while 49% were able to walk independently (mRS 0–3) [36]. Second, recovery to good functional outcome was well documented across the body of evidence despite a high NIHSS assessed at various time-points during the index hospitalization. No single NIHSS threshold measured in the index hospitalization, especially in the ICU, can be used to reliably prognosticate a *poor* outcome in an individual patient, since factors such as young age and plasticity may result in dramatic long-term improvements with rehabilitation.

The predictive value of the NIHSS for poor outcomes does, however, increase with time: later measurements are progressively more reflective of final outcome [42, 43]. Since most patients demonstrate neurological improvement over time, the NIHSS that best predicts poor long-term functional outcome decreases with time. In the NINDS recombinant tissue Plasminogen Activator (rtPA) trial, NIHSS > 22 at 24 h had a positive predictive value (PPV) of 98% for inability to ambulate independently at 3 months, while NIHSS > 16 at 7–10 days had a PPV of 92% for prediction of the same outcome [44]. In a 2005 study, NIHSS ≥ 6 at 7 days after admission predicted poor functional outcome (inability to ambulate, death or Barthel Index < 60) with sensitivity of 84%, specificity of 92%, PPV of 77%, and NPV of 95% [43]. A consistent time of assessment and NIHSS threshold have not been identified, however. High-quality prospective studies in the era of third-generation thrombectomy devices are required to validate the predictive value of the NIHSS (or clinical prediction models that incorporate the NIHSS) measured at later time-points during the initial hospital stay [45].

*PICO question 3*: “When counseling critically ill patients with AIS or their surrogates, should hemorrhagic transformation alone be considered a reliable predictor of poor functional outcome assessed at 3 months or later?”.

*Description of the predictor*: Hemorrhagic conversion after ischemic stroke is the occurrence of bleeding within an area of the brain initially affected by ischemia. Hemorrhagic transformation has been variably defined across the literature. The European Cooperative Acute Stroke Trials (ECASS) classification includes three radiological categories: no HT, hemorrhagic infarction (HI), featuring small petechial hemorrhages along the infarct margins, and parenchymal hematoma (PH), characterized by confluent hematoma [46, 47]. The presence or absence of clinical deterioration (symptomatic vs. asymptomatic) may be incorporated into the classification, in addition to radiological features [48–50].

*Recommendation*: When counseling critically ill patients with AIS or their surrogates, we suggest hemorrhagic transformation alone not be considered a reliable predictor of poor functional outcome assessed at 3 months or later *(weak recommendation; very low quality evidence).*

*Rationale*: The body of evidence was downgraded once for inconsistency, once for indirectness, once for imprecision, and twice for risk of bias in the domains of study participation, attrition, prognostic factor measurement, outcome measurement, confounding, statistical analysis, and self-fulfilling prophecy. Across the body of evidence, a good functional outcome was feasible despite the occurrence of hemorrhagic transformation, even with concomitant clinical deterioration [51–58]. While more severe hemorrhagic transformation might be expected to have a greater impact on neurological status and functional outcome, there is an insufficient body of evidence evaluating the degree of hemorrhagic transformation as a predictor of poor outcome.

*PICO question 4*: “When counseling critically ill patients with AIS or their surrogates, should infarct size alone be considered a reliable predictor of poor functional outcome assessed at 3 months or later?”.

*Description of the predictor*: Infarct size is defined the total volume of brain tissue permanently damaged owing to insufficient blood flow. Early estimation of infarct size on computed tomography (CT) may be performed using the Alberta Stroke Program Early CT Score (ASPECTS), through identification of regions with ischemic changes [59]. Magnetic resonance imaging (MRI) with diffusion-weighted imaging (DWI) is more sensitive than CT for acute and hyperacute delineation of infarct size [60]. Final infarct size is generally established by day 30 [61]. True quantification of infarct volume requires manual tracing of the infarct perimeter, while other techniques include pixel thresholding for automated differentiation, stereological counting grids for statistical volume estimation, and measuring the largest diameters of the infarct [62]. Definitions and volume thresholds for “large” core infarcts have varied across the literature [63–66].

*Recommendation*: When counseling critically ill patients with AIS or their surrogates, we suggest infarct size alone not be considered a reliable predictor of poor functional outcome assessed at 3 months or later *(weak recommendation; very low quality evidence).*

*Rationale*: The body of evidence was downgraded once for inconsistency, once for indirectness, once for imprecision, and twice for risk of bias in the domains of study participation, attrition, prognostic factor measurement, outcome measurement, confounding, statistical analysis, and self-fulfilling prophecy. Substantial inconsistency was present in the body of evidence. A large core infarct at initial presentation could not, in isolation, be considered a reliable predictor of poor outcome. Infarct location and morphology may impact outcomes independent of size: the presence of infarcts confined to gray matter, infarcts sparing the corticospinal tract, and scattered (compared with territorial) infarcts have been shown to predict better outcomes, independent of infarct size [67–70]. The extent and neurological impact of cerebral edema associated with a large infarct may also impact outcome [71]. Older patients with greater cerebral atrophy may be less likely to suffer acute neurological deterioration from large infarcts [71].

Of note, several recent clinical trials of patients with large core infarcts (defined by an ASPECTS score 3–5 or core infarct volume ≥ 50 mL on perfusion imaging) have demonstrated the potential for recovery to ambulation, or even functional independence, with endovascular therapy [63]. Across trials, about 41% of patients who received endovascular therapy and 24% of patients treated medically regained independent ambulation, while 23% of patients who received endovascular therapy and 9% of patients treated medically regained functional independence. The results of clinical trials do not reflect the outcomes likely to be observed in real-world clinical practice: for example, these trials mostly excluded patients older than 80 years and all patients with baseline functional impairment. In addition, early trial termination may have resulted in overestimation of treatment effect. However, these trials do demonstrate the feasibility of good long-term outcome despite a large infarct core at presentation. We suggest that statistics from clinical trials of revascularization in patients with a large-infarct core be considered a best-case estimate of outcomes at the time of prognostication and counseling, and the entire range of possible outcomes should be discussed with patients and surrogates.

*PICO question 5*: “When counseling critically ill patients with AIS or their surrogates, should a history of previous stroke alone be considered a reliable predictor of poor functional outcome assessed at 3 months or later?”.

*Description of the predictor*: One in four patients presenting with stroke have had a prior stroke [1]. Across studies, these historical data were collected through a combination of patient- and/or surrogate-reported past medical history and examination of medical records.

*Recommendation*: When counseling of critically ill patients with AIS or their surrogates, we suggest a history of previous stroke alone not be considered a reliable predictor of poor functional outcome assessed at 3 months or later *(weak recommendation; very low quality evidence).*

*Rationale*: The body of evidence was downgraded once for inconsistency, once for imprecision, once for indirectness, and twice for risk of bias in the domains of study participation, attrition, prognostic factor measurement, outcome measurement, confounding, statistical analysis, and self-fulfilling prophecy. Across the body of evidence, a good long-term functional outcome was feasible in patients with a history of previous stroke.

*PICO question 6*: “When counseling critically ill patients with AIS or their surrogates, should successful revascularization alone be considered a reliable predictor of good functional outcome assessed at 3 months or later?”.

*Description of the predictor*: Revascularization or recanalization was determined after acute reperfusion therapies including thrombolysis, mechanical thrombectomy, or both. Revascularization/recanalization was most often classified according to the modified Thrombolysis in Cerebral Infarction (mTICI) scale, with successful revascularization defined as mTICI grade 2b (antegrade reperfusion of more than half of the previously occluded target artery ischemic territory), 2c (near-complete perfusion except for slow flow or distal emboli in a few distal cortical vessels), or 3 (complete antegrade reperfusion of the previously occluded target artery ischemic territory, with absence of visualized occlusion in all distal branches) [72, 73]. The thrombolysis in myocardial infarction (TIMI) grade has also been used: 0 = no recanalization, 1 = minimal recanalization, 2 = partial recanalization, and 3 = complete recanalization [74]. Repeat or follow-up imaging was most often obtained within 6–24 h after initial imaging, but could occur as early as 2 h and as late as 48 h. Typically revascularization was dichotomized into good or poor/none, though sometimes categorized as none, partial, or complete.

*Recommendation*: When counseling critically ill patients with AIS or their surrogates, we suggest successful revascularization alone not be considered a reliable predictor of good functional outcome assessed at 3 months or later *(weak recommendation; low quality evidence).*

*Rationale*: The body of evidence was downgraded once for imprecision and once for risk of bias in the domains of study participation, attrition, outcome measurement, confounding, statistical analysis, and self-fulfilling prophecy. The body of evidence was overall robust in identifying successful revascularization in patients who undergo thrombectomy as an *independent* predictor of good outcome. However, successful revascularization alone does not guarantee a good outcome since several patients will suffer infarct expansion prior to completion of revascularization or suffer a subsequent complication that compromises neurological recovery. Across clinical trials of thrombectomy, long-term recovery to functional independence was observed in fewer than half of patients in the intervention arm, despite rates of successful revascularization > 70% [36].

*PICO question 7*: “When counseling critically ill patients with AIS or their surrogates, should early neurological improvement (ENI) assessed 24 h following revascularization, or 24 h following admission for patients who are not candidates for revascularization, be considered a reliable predictor of good functional outcome assessed at 3 months or later?”.

*Description of the predictor*: Early (or rapid) neurological improvement (ENI), which is most commonly observed following successful revascularization but may also occur spontaneously, occurs in approximately 20–40% of patients with AIS [43, 75–87]. We defined this predictor as a ≥ 8 point improvement in the NIHSS, or an improvement to an NIHSS of 0 or 1 at 24 h), consistent with commonly used criteria in the literature [75, 77–79, 83]. Alternate definitions of ENI include, among others, an improvement in the NIHSS of 4 or more points [84], 1 or more points [85], 12% [86], or 30% [87]. The primary body of evidence for ENI as a predictor of outcome is from clinical trials and observational studies of revascularization therapy for AIS. However, ENI may also occur as a result of spontaneous recanalization, fragmentation of clot, or resolution of hemodynamic disturbance.

*Recommendation*: When counseling critically ill patients with AIS or their surrogates, we suggest early neurological improvement (ENI) assessed 24 h following revascularization, or 24 h following admission for patients who are not candidates for revascularization, be considered a moderately reliable predictor of good functional outcome assessed at 3 months or later. A good outcome is expected in patients with recovery to an NIHSS of 0 to 1 at 24 h *(weak recommendation; low quality evidence).*

*Rationale*: The body of evidence was downgraded once for imprecision and once for risk of bias in the domains of study participation, attrition, outcome measurement, confounding, statistical analysis, and self-fulfilling prophecy. About 61–84% of patients with a ≥ 8 point improvement in the NIHSS, or an improvement to an NIHSS of 0 or 1 at 24 h, will return to functional independence [75, 77–79, 83]. While the greatest certainty of recovery is in patients who improve to a NIHSS of 0 to 1 at 24 h, ongoing neurological recovery, more often than not, leads to functional independence by 3 months in patients who improve by ≥ 8 points but to an NIHSS > 1 at 24 h. Regardless of definition, the literature overwhelmingly demonstrates that ENI is an independent predictor of return to long-term functional independence at 3 months, and that a good outcome is observed in over 60% of patients with such improvement [43, 75–87]. A good outcome is not certain, since residual deficits are occasionally disabling despite a large magnitude of improvement. In addition, post-recanalization intracerebral hemorrhage or recurrent stroke may occur following initial improvement. Medical complications may also restrict a return to functional independence. While ENI may occur without revascularization therapy, data from clinical trials confirm that revascularization therapy—especially mechanical thrombectomy—is associated with a greater magnitude of neurological improvement at 24 h than with medical therapy alone [36, 81, 88]. It should be noted that the absence of ENI does not preclude neurological recovery, since the body of evidence demonstrates that delayed neurological improvement to varying degrees is seen in the majority of patients with AIS and can lead to a good functional outcome. In one study, over one in five patients with successful endovascular recanalization who failed to demonstrate ENI nevertheless achieved functional independence at 3 months [89].

In addition to improvements in the NIHSS, early improvement in specific neurological deficits is associated with recovery of function in the associated domains (Table 6). Most hemiparetic patients with some voluntary finger extension and shoulder abduction of the hemiplegic limb 2 days following the stroke will regain at least some hand dexterity at 6 months [90]. Most patients able to maintain sitting balance for 30 s and any muscle contraction in the paretic leg within the first 72 h will regain ambulation at 6 months [91].

**Table 6:** Recovery following domain-specific impairments in acute ischemic stroke

Domain	Proportion of patients who recover function within the domain	Time period of recovery	Early improvement and prediction of recovery	Comments
Upper limb function, hand dexterity [148]	Mild (MRC 4) initial impairment: 70–100% recover function Moderate (MRC 2–3) initial impairment: 30–50% recover function Severe initial impairment/flaccid weakness (MRC 0–1): 10–33%% recover function	Mild: 5–8 weeks Moderate: 10–12 weeks Severe: 11–17 weeks Recovery can extend beyond these periods in a subset of patients [189]	Most patients with voluntary finger extension and shoulder abduction of the hemiplegic limb at 2 days regain at least some hand dexterity at 6 months [148]	Recovery of upper limb function often defined by ability to perform feeding and grooming tasks
Lower limb function [149]	Variable, depending on severity of initial weakness: 10–95% May occur more frequently than upper limb recovery	Variable, depending on severity of initial weakness: 3–17 weeks, longer with more severe initial weakness. May occur faster than upper limb recovery Recovery can extend beyond this period in a subset of patients [189]	Most patients (98%) able to maintain sitting balance for 30 s and any muscle contraction in the leg within the first 72 h will regain ambulation at 6 months [149]	Recovery of lower limb function often defined by recovery of ambulation, which requires less dexterity and may be aided by muscle tone
Aphasia [49]	Most patients will demonstrate improvement over time, with magnitude of recovery dependent on initial severity	Most improvement occurs within 2 weeks for mild initial aphasia, and 10 weeks for severe aphasia [196]. However, recovery beyond 6 months can occur [49, 51]	–	–
Unilateral spatial neglect	The majority (50–80%) of patients with hemineglect will demonstrate resolution or major improvement in this domain at 6 months	Most recovery occurs within 3–6 months	–	Persistence of spatial neglect is associated with poor functional recovery
Unilateral visual field defect	Overall about 40–50% demonstrate resolution at 3 months [203]. At 1 month, 17% of complete and 72% of partial unilateral visual field defect demonstrated complete recovery [202].	Most of the improvement in visual field defects likely occurs within 1–3 months [203]	–	–
Unilateral sensory loss	Most patients demonstrate significant improvement or resolution of sensory deficits	Most recovery occurs within 3 months but may continue up to 12 months	–	Hyperesthesia and thalamic pain syndromes can also cause significant disability

In addition, hyperglycemia and poor cerebral collateral circulation status were not considered reliable predictors of poor functional outcome in the context of counseling critically ill patients with AIS or their surrogates (Tables 3 and 4, rationale in Supplementary Appendix 3).

### Recommendations: Clinical Prediction Models as Predictors of Functional Outcome

*PICO question 10*: “When counseling critically ill patients with AIS or their surrogates, should the Acute Stroke Registry and Analysis of Lausanne (ASTRAL) score be considered a reliable predictor of poor functional outcome assessed at 3 months or later?”.

*Description of the predictor*: The ASTRAL score, originally developed by Ntaios et al. in 2012 from a single-center stroke center in Lausanne, Switzerland, includes six clinical and laboratory predictors of poor functional outcome, defined as a modified Rankin Scale (mRS) score of 3–6 at 90-days [92]. These variables are admission glucose, age, any stroke-related visual field defect, level of consciousness, symptom onset to treatment time, and stroke severity (NIHSS).

*Recommendation*: When counseling critically ill patients with AIS or their surrogates, we suggest the ASTRAL score not be considered a reliable predictor of poor functional outcome assessed at 3 months or later *(weak recommendation; low quality evidence).*

*Rationale*: The body of evidence was downgraded once each for indirectness and for risk of bias in the following domains: participants, predictors and self-fulfilling prophecy, with concern about applicability in the participants domain. Several external validation studies have demonstrated acceptable discrimination [93–95], but not all [96]. Calibration was assessed in several of these cohorts and demonstrated a good fit. This model could not be recommended for routine clinical use primarily because, similar to other AIS prediction models, it is based entirely on admission variables and does not consider the impact of thrombectomy or other effective therapeutic interventions. Successful revascularization is a strong independent predictor of outcome in patients with AIS [36], and over a third of patients will demonstrate rapid neurological improvement in the first several days [43, 75–87], which is also an independent predictor of outcome. Baseline variables alone may not adequately explain outcome in the current era of stroke therapy. The score also does not consider pre-stroke dependency, imaging, or clinical events during hospitalization.

*PICO question 11*: “When counseling critically ill patients with AIS or their surrogates, should the DRAGON, MR-DRAGON, and MT-DRAGON scores be considered reliable predictors of poor functional outcome assessed at 3 months or later?”.

*Description of the predictor*: The DRAGON score includes the variables within the acronym: a dense cerebral artery or early infarct signs on computed tomography, pre-stroke modified Rankin Scale (mRS) score, age, baseline glucose level, onset to treatment time, and baseline NIHSS. The DRAGON score predicts a trichotomized outcome based on the mRS: good (mRS 0–2), poor (mRS 3–4), and miserable (mRS 5–6). A lower score indicates a higher likelihood of a good outcome [97]. The MR-DRAGON score uses the proximal middle cerebral artery (MCA) occlusion on magnetic resonance angiography instead of the CT hyperdense MCA sign, and diffusion-weighted imaging Alberta Stroke Program Early Computed Tomography Score (DWI ASPECTS) ≤ 5 instead of CT early infarct [98]. The MT-DRAGON score, which is specific to patients treated with thrombectomy, uses time to groin puncture instead of onset-to-IV-rtPA time and the site of large vessel occlusion [99].

*Recommendation*: When counseling critically ill patients with AIS or their surrogates, we suggest the DRAGON, MR-DRAGON, and MT-DRAGON scores not be considered reliable predictors of poor functional outcome assessed at 3 months or later *(weak recommendation; very low quality evidence).*

*Rationale*: The body of evidence was downgraded once for indirectness and twice for risk of bias in the following domains: participants, predictors, and self-fulfilling prophecy, with concern about applicability in the participants domain. In the original study, the area under the curve (AUC) was 0.84 in the derivation cohort and 0.80 in the validation cohort. Multiple subsequent external validation studies have revealed good discrimination and calibration [97, 100–102]. This score could not be recommended for the prediction of poor outcome because, while the score includes important imaging data that reflect completed infarction, it reflects the patient’s status at admission and does not include any measures of the efficacy of therapeutic intervention, or clinical evolution during the hospital admission. Interobserver reliability for interpretation of early infarct signs on CT may be variable [103]. While the MR-DRAGON and MT-DRAGON scores may provide a more objective assessment of the extent of completed infarction, validation in other settings and populations is required.

*PICO question 12*: “When counseling critically ill patients with AIS or their surrogates, should the iScore prediction model be considered a reliable predictor of poor functional outcome assessed at 3 months or later?”.

*Description of the predictor*: The Ischemic Stroke Predictive Risk Score (iScore) was developed to predict the risk of death and disability (mRS ≥ 3) or institutionalization at discharge. This scoring system comprises a combination of clinical parameters and chronic comorbid conditions: age, gender, stroke severity (measured by the NIHSS or the Canadian Stroke Scale), stroke subtype, history of atrial fibrillation, coronary artery disease, congestive heart failure, cancer, kidney disease requiring dialysis, smoking history, hyperglycemia at admission, and pre-stroke dependency [104]. The score has also been used to predict clinical response and risk of complications in patients undergoing thrombolytic therapy for ischemic stroke. While the score was originally developed to predict early (30 day) death or disability, subsequent external validation studies have examined the predictive accuracy of the score for 90-day [105–107] and 180-day poor functional outcome (mRS > 2) [108].

*Recommendation*: When counseling critically ill patients with AIS or their surrogates, we suggest the iScore prediction model not be considered a reliable predictor of functional outcome assessed at 3 months or later *(weak recommendation; very low quality evidence).*

*Rationale*: The body of evidence was downgraded once for indirectness and twice for risk of bias in the following domains: participants, predictors, and self-fulfilling prophecy, with concern about applicability in the participants domain. The iScore demonstrates acceptable discrimination for both mortality and functional outcome [101, 104, 109]; however, calibration was imperfect, on the basis of assessment with the Hosmer–Lemeshow test in the original study [101]. Similar to other prediction models, the iScore does not assess the impact of therapeutic intervention or clinical evolution during the hospital stay, and could not be considered reliable for the prediction of poor outcome.

*PICO question 13*: “When counseling critically ill patients with AIS or their surrogates, should the THRIVE/THRIVE-c/THRIVE-EVT scores be considered reliable predictors of good functional outcome assessed at 3 months or later?”.

*Description of the predictor:* The THRIVE score was originally derived from two single-arm trials (MERCI and Multi-MERCI) of 305 patients with AIS who underwent mechanical thrombectomy with a first-generation device, for the prediction of good functional outcomes (mRS 0–2) and mortality at 90 days [110]. The variables within the original THRIVE score were age, NIHSS, presence of diabetes mellitus, presence of hypertension, and presence of atrial fibrillation. An integer score between 0–9 was generated, with higher scores associated with a lower probability of a good outcome. The THRIVE score has also been used to predict intracerebral hemorrhage and outcomes following intravenous thrombolysis [111–113]. Subsequent studies validated the THRIVE score for prediction of outcome following thrombectomy with later-generation devices [114, 115]. In 2015, the THRIVE-c score was described, with the same variables, but with age and NIHSS included as continuous variables to improve model performance [116]. More recently, the THRIVE-EVT score was developed [117]. In addition to the THRIVE-c variables, THRIVE-EVT includes randomization to endovascular therapy as a variable, and, where available, the Alberta Stroke Program Early CT Score (ASPECTS). The THRIVE-EVT score estimates the probability of good functional outcome with and without endovascular treatment in an individual patient.

*Recommendation*: When counseling critically ill patients with AIS or their surrogates, we suggest the THRIVE/THRIVE-c/THRIVE-EVT scores not be considered reliable predictors of good functional outcome assessed at 3 months or later *(weak recommendation; low quality evidence).*

*Rationale*: The body of evidence was downgraded twice for risk of bias in the following domains: participants, predictors, and self-fulfilling prophecy. The THRIVE, THRIVE-c, and THRIVE-EVT scores were derived and validated in cohorts of patients treated with thrombectomy and therefore directly relevant to patients admitted to the ICU for post-procedural care, who fall within the scope of these guidelines. However, while the THRIVE score has undergone extensive external validation, discriminative performance is likely insufficient for the prognostication of outcome in the context of counseling surrogates of critically ill patients at risk for WLST. Despite derivation from endovascular clinical trial datasets, the score does not include successful revascularization as a variable. Similar to all other prediction models, the THRIVE score and its variants do not consider the clinical status of the patient following intervention. Pre-stroke dependency is not considered. While the THRIVE-EVT score may help estimate the probability of good outcome with and without endovascular therapy, the score cannot be used to exclude patients from intervention, who otherwise meet the eligibility criteria of major endovascular clinical trials.

### Good Practice Statements

In accordance with recommendations of the GRADE network, these statements were considered by the panel to be actionable, supported by indirect evidence where appropriate, and essential to guide the practice of neuroprognostication [118]. The good clinical practice reflected in these statements was considered by the panel to be unequivocally beneficial.

*1.* *Good practice statement 1*: We recommend that prognostication should be multimodal, with consideration of the complete clinical condition, and never based on a single variable. (Strong recommendation, evidence cannot be graded).

*Rationale: *This acknowledges the complex pathophysiology of stroke, individual patient variability, the dynamic nature of stroke progression, comorbidities, and prestroke functional baseline.

*2.* *Good practice statement 2*: We recommend that the assessment of neurological prognosis be performed in the absence of potential confounders of the neurological exam. (Strong recommendation, evidence cannot be graded).

*Rationale: *Confounders such as sedative medications, metabolic imbalances, or systemic illnesses may mask the patient’s true neurological status [119–121]. Resolution of confounders may clarify the presence of an encouraging neurological examination, with resolution of hemiparesis or other deficits.

*3. Good practice statement 3*: We recommend that preexisting functional impairment and comorbidities be considered when formulating a prognosis. (Strong recommendation, evidence cannot be graded)

*Rationale: *Prior limitations in mobility or cognition impact the baseline from which recovery is measured, while diseases such as congestive heart failure, chronic obstructive pulmonary disease, or prior neurodegenerative conditions may complicate or slow the recovery process [122–124].

*4.* *Good practice statement 4*: We recommend that counseling of critically ill patients with AIS expected to survive the index hospital admission, and/or their surrogates, include discussion of long-term functional outcomes. Discussion should not focus on day-to-day changes and in-hospital outcomes alone. (Strong recommendation, evidence cannot be graded).

*Rationale:* Counseling in the ICU is sometimes limited to the possibility of survival and a focus on short-term changes in various organ systems. Discussions may therefore be focused on daily changes in ventilator settings, blood pressure management, or laboratory derangements. While such discussions are important, several studies suggest families and surrogates experience frustration with information perceived as incomplete, unrealistic, or not meaningful [125]. While families and surrogates prefer communication of hope or guarded optimism, honesty about long-term prognosis, even if poor, is valued over withholding of information [125]. While discussions of long-term outcome may not be appropriate in the early period of the ICU stay when the patient’s condition is rapidly evolving, discussion at a reasonable time-point during the ICU stay is critical. Discussions on the importance of rehabilitation therapies such as physical therapy (PT), occupational therapy (OT), and speech therapy is also necessary in patients expected to survive.

*5.* *Good practice statement 5*: We recommend that acceptable outcomes for individual patients serve as the basis for prognostication and shared decision-making. (Strong recommendation, evidence cannot be graded).

*Rationale: *The extent of functional improvement that is considered acceptable can vary greatly between individuals. Establishing the level of functional recovery acceptable to an incapacitated patient may be complicated by the disability paradox [126, 127]. Some patients with marked functional disability from their stroke report satisfaction with their health and quality of life [128–130]. This phenomenon may reflect both variability in perceptions, and adaptation to long-term disability. Multiple discussions are often necessary to establish acceptable outcomes, which can serve as the basis for shared decision-making.

*6.* *Good practice statement 6*: We recommend avoiding premature neuroprognostication in critically ill patients with AIS, including patients with severe ischemic stroke. While early neurological improvement (ENI) following revascularization may clarify a likely good outcome at 24 h, the appropriate period of observation in an individual patient should be determined with careful consideration of factors such as the anticipated evolution of neurological illness, potential confounders of the neurological exam, and completion of appropriate diagnostic imaging. (Strong recommendation, evidence cannot be graded).

*Rationale*: The optimal time-point for accurate neuroprognostication following revascularization depends on several factors, including stroke severity, baseline comorbidities, and the presence of confounders. In addition, magnetic resonance imaging (MRI) or repeat CT may clarify final infarct size but may not be available for several days. The clinical neurological status of a critically ill, intubated patient with AIS with medical complications should not be assessed until confounders such as sedation and toxic–metabolic encephalopathy are no longer present, which may require many days, or longer. The value of ENI as a predictor of functional outcome has been addressed separately in the corresponding PICOTS question.

*7.* *Good practice statement 7*: We recommend that critically ill patients with *severe* AIS, defined as NIHSS > 15, and/or their surrogates, be counseled that incremental functional recovery may occur up to 6 months, and sometimes longer, following the stroke, whereas most patients with AIS of mild to moderate severity will achieve the majority of their functional recovery within 3 months. (Strong recommendation, evidence cannot be graded).

*Rationale*: While patients with mild to moderate stroke are more likely to achieve functional independence than patients with more severe strokes, the time duration to a plateau in recovery is directly proportional to initial severity [131, 132]. Stroke severity is variably defined, but a common definition of “severe” stroke uses a NIHSS threshold > 15 [38, 39]. The 3-month time-point, commonly used for the primary outcome measure in AIS clinical trials and observational research [133, 134], may not be applicable to patients with more severe stroke admitted to the ICU. Longitudinal studies have demonstrated that, while the majority of functional recovery in patients with mild to moderate AIS occurs within 3 months, patients with severe stroke frequently demonstrate continued functional improvement up to 5–6 months [131, 135], and sometimes longer [132, 136]. Improvement beyond 6 months may occur in individual patients, particularly in domains such as language and cognitive function that are not captured by the mRS [137–139]. Insufficient evidence currently exists on the likelihood and predictors of recovery beyond 6 months.

*8.* *Good practice statement 8*: We recommend that critically ill patients with AIS who require artificial enteral nutrition, and/or their surrogates, be counseled that recovery of swallow function and removal of the feeding tube is likely, but not certain, within a period of several months among survivors. (Strong recommendation, evidence cannot be graded).

*Rationale*: In addition to concerns about overall functional disability, surrogates of critically ill patients with AIS with dysphagia frequently express concern about permanent dependence on feeding tubes. The incidence of oropharyngeal dysphagia following AIS ranges from 37% to 45% with the use of a bedside water-swallow screen, to 64–78% with instrumental testing [140]. Age, prior stroke, stroke severity, and stroke volume are independent predictors of dysphagia on admission [141, 142], which increases the risk of aspiration and pneumonia [140].

*The need for prolonged artificial nutrition may be associated with an overall high risk of mortality and long-term disability* [143, 144]. In the Feed Or Ordinary Diet (FOOD) clinical trial of patients randomized to receive nasogastric or percutaneous gastrostomy (PEG) tube feeding within 30 days of AIS, 48% of patients were dead, and 37% were unable to walk independently (mRS > 3) at 6 months [144]. However, most patients with AIS with dysphagia on admission do not require *prolonged* tube feeding: in one study, over two-thirds of all patients with post-stroke dysphagia regained the ability to swallow within 1 month of the stroke [143]. Utilization of PEG tubes for prolonged artificial enteral nutrition varies across the USA, between 0% and 26% of patients with AIS [145]. Increased stroke severity, occurrence of bihemispheric infarcts, and brainstem infarction increase the likelihood of prolonged dysphagia and PEG placement [146, 147]. Among patients who do require prolonged tube feeding, rates of recovery of swallow function and feeding tube removal have varied in the literature, primarily as a result of variability in the time-point of assessment [148]. Most studies that assess rates of PEG removal at 6–12 months suggest the majority of survivors will recover swallow function within this time frame, allowing for removal of the feeding tube [146, 148, 149]. In one study, over 80% of patients with AIS with dysphagia had recovered swallow function by 6 months [149]. In a study of patients with AIS admitted to inpatient rehabilitation with a percutaneous gastrostomy (PEG) tube, recovery of swallow function and tube removal occurred in about one-third of patients prior to discharge and three-quarters of patients at 1 year [146]. While data derived from patients admitted to inpatient rehabilitation units are not directly applicable to the critically ill AIS population, it is reasonable to counsel patients and/or surrogates that recovery of swallow function generally does occur. Clinicians should consider individual patient factors when discussing the likelihood of long-term recovery of swallow function. There is no high-quality evidence on predictors of *permanent* dependence on artificial enteral nutrition following AIS.

*9.* *Good practice statement 9*: We recommend that critically ill patients with AIS who require tracheostomy, and/or their surrogates, be counseled that successful decannulation is possible within a period of several months among survivors. (Strong recommendation, evidence cannot be graded).

*Rationale*: In addition to concerns about long-term functional recovery, surrogates of critically ill patients with AIS on mechanical ventilation who require tracheostomy often express concern about permanent dependence on an artificial airway. Studies suggest that successful decannulation occurs within 6–12 months in 30–65% of patients with AIS who require tracheostomy [150–155]. Studies of patients with all forms of brain injury suggest that age, etiology, severity of injury, anatomical location, evidence of aspiration, cough strength, threshold to perform tracheostomy, and functional improvements are predictors of decannulation [25, 151–155]. There is limited longitudinal data specific to patients with AIS on functional recovery following tracheostomy. In some studies, over one in five patients with AIS who required tracheostomy achieved independence in basic activities of daily living at 1 year [25, 152, 156, 157]. Unlike some patients who require tracheostomy for respiratory failure [158], patients with AIS with tracheostomy dependence are likely to demonstrate persistent dysphagia, and continue to receive artificial enteral nutrition [150].

*10.* *Good practice statement 10*: We recommend that discussions of prognosis and goals of care for patients with large hemispheric infarction occur prior to neurological deterioration where feasible, to allow patients and surrogates sufficient time to make informed decisions on decompressive craniectomy and other therapeutic interventions. (Strong recommendation, evidence cannot be graded).

*Rationale*: Large hemispheric infarctions have previously been defined by the German Society for Neuro-Intensive and Emergency Medicine (Deutsche Gesellschaft Fur NeuroIntensiv-und Notfallmedizin) NCS/DGNI as affecting the total or subtotal territory of the middle cerebral artery (MCA) and at least partially affecting the basal ganglia, with or without involvement of adjacent territories [159]. These strokes typically occur following occlusion of the internal carotid artery or MCA, and are associated with a NIHSS > 15. Such patients are at high risk for progressive cerebral edema that leads to uncal herniation and death, with mortality of about 78% without surgical decompression [160]. These patients frequently require endotracheal intubation, prolonged artificial enteral nutrition, and tracheostomy [161]. The extent of infarction is typically evident on imaging prior to clinical neurological deterioration, which provides a window of hours to days for counseling and decisions on surgical decompression. While decompressive craniectomy is usually life-saving, and may improve functional outcome in select patients [162, 163], decision-making regarding surgical decompression can be challenging. Patients under 60 years of age who undergo decompression within 48 h are most likely to achieve independent ambulation, or even independence to basic activities of daily living, following decompressive craniectomy [162, 163]. However, a majority of patients will not regain independent ambulation and will be severely disabled (mRS > 3) [162, 163]. Patients and surrogates should be provided sufficient time to receive and process complex information, have conversations with other family members and friends, ask questions, and formulate a decision. Rushed conversations about prognosis and goals of care that occur following neurological deterioration may not allow families and surrogates sufficient time to make informed decisions that reflect the patient’s wishes.

Prognostication of patients with large hemispheric infarction is frequently based on the results of major clinical trials of surgical decompression. In a meta-analysis of seven trials from six countries, overall 37% of patients regained the ability to ambulate independently (mRS ≤ 3) and 17% regained functional independence (mRS ≤ 2) following surgical decompression, while only 15% of patients regained independent ambulation (*p* < 0.001 for the crude odds ratio) and 5% regained functional independence (*p* = 0.04) with medical management [162]. Among patients over the age of 60 years, only 28% of patients who underwent surgical decompression and 10% of patients managed with medical care achieved independence in ambulation. Too few patients older than 60 years achieved functional independence to permit meaningful analysis. Results were highly varied, however. Across the seven trials, the rates of independent ambulation in patients older than 60 years ranged from 0% to 66% among patients who underwent surgical decompression and from 0% to 23% in patients who underwent medical management. Prognostication based on the results of these clinical trials is inherently flawed, since outcome data were impacted by eligibility criteria, early trial termination, and small sample size, which may have resulted in an overestimation of treatment effect. Cognitive outcome and quality of life were not assessed in most studies. We therefore suggest that statistics from clinical trials be considered a best-case estimate of outcomes in this context, and the entire range of possible outcomes be presented to patients and surrogates.

### Approach to Neuroprognostication

**Fig. 2:**
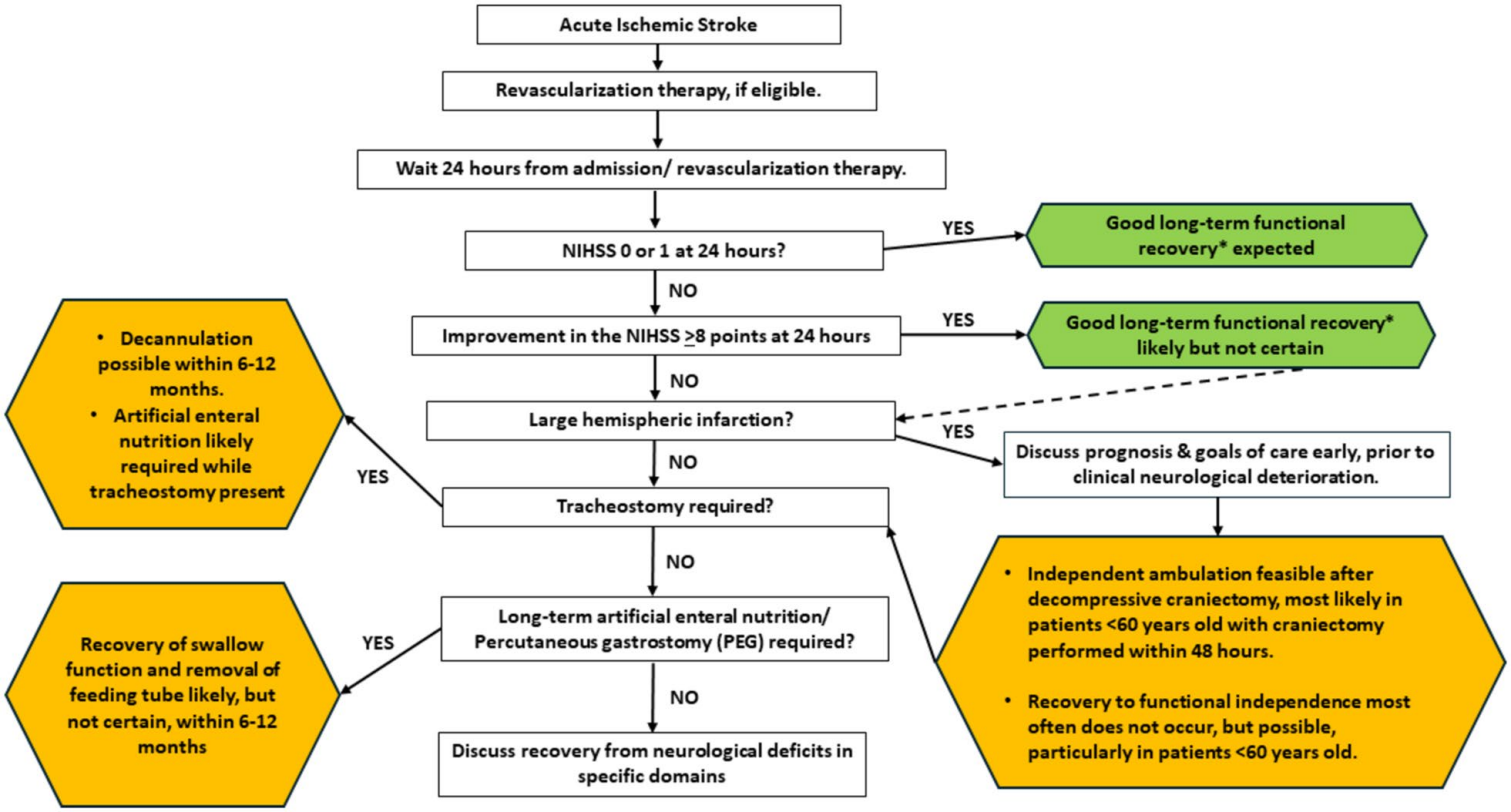
Algorithm for neuroprognostication in patients with AIS who require ICU admission. *In this context, good functional recovery is defined as the equivalent of modified Rankin Score ≤ 2: the ability to manage the patient’s own affairs. NIHSS: National Institutes of Health Stroke Scale. The interrupted line (–) in the algorithm represents a lower probability event: a patient with a ≥ 8 point improvement in the NIHSS with evidence of a large hemispheric infarct 24 h following admission and revascularization

A suggested approach to neuroprognostication for patients with AIS admitted to the ICU is shown in Fig. 2. Table 6 describes specific domains of recovery in these patients. Neuroprognostication in the context of critical illness, where a prediction of poor outcome may lead to withdrawal or limitation of life support, is typically more challenging than prognostication of functional recovery in patients with AIS in a rehabilitation or general care unit. A critical element in prognostication following AIS is the outcome of attempted revascularization. While clinical evolution and in-hospital events are an important prognostic consideration in all neurocritical care disease states, the availability of a highly effective therapy for AIS may result in especially dramatic changes in neurological status, and therefore prognosis, within the first few days following presentation.

### Gaps and Opportunities in the AIS Neuroprognostication Literature

Several opportunities exist for future neuroprognostication research in critically ill patients with AIS, based on the most common sources of bias identified in this systematic review:

1. *Reassessment after revascularization therapy*. In addition to the baseline assessment, prognostication studies (including studies of prediction models) in the current era of stroke therapy should consistently assess the NIHSS, or other measures of neurological status, at successive time-points following revascularization therapy (or following admission, in patients ineligible for revascularization therapy), starting at 24 h. Beyond the 24-h measurement, which is widely documented in stroke registries and clinical trials, assessment at 7 days or later may improve predictive accuracy. Similarly, prediction models derived from cohorts of patients who undergo endovascular therapy should consider both revascularization grade and neurological improvement following therapy.
2. *Timing of outcome assessment: severe stroke*. Functional outcome should ideally be assessed at 180 days or later, in addition to the commonly performed 90-day assessment, in patients with severe stroke.
3. *Patient-centered and patient-reported outcomes*. Future research should assess patient-centered outcomes beyond functional outcome, such as cognitive outcome. In addition, prognostication studies should assess patient-reported outcomes (rather than clinician-reported outcomes alone) such as functional status, quality of life, return to work, post-stroke depression, anxiety, and fatigue [164].
4. *Stroke registries*. Data from widely available multicenter stroke registries may provide greater statistical power and generalizability than single-center studies and datasets from clinical trials. However, these registries should ideally include a wide range of common data elements, including successive neurological assessments, details of revascularization therapy, level of prestroke dependence, major comorbidities, and assessment of function outcome at 90 days and beyond.
5. *Measurement of infarct volume*. Prediction models should ideally consider objective, quantitative measures of final infarct volume beyond CT ASPECTS alone.
6. *Outcomes following tracheostomy and PEG placement*. Multicenter longitudinal prospective data on rates of PEG removal, tracheostomy decannulation, and recovery of functional outcome in critically ill patients with AIS who require these interventions are required, beyond the context of patients admitted to inpatient rehabilitation units alone.
7. *Prediction model performance*. Studies of prediction models should consistently evaluate model calibration, in addition to discrimination, in populations and datasets outside the primary derivation and validation cohort(s).
8. *Impact of prognostic model use in routine care*. The impact of the systematic use of prognostic models to guide shared decision-making on rates of withdrawal of life-sustaining therapy and patient-centered outcomes is incompletely understood. Prospective studies comparing systematic model use for shared decision-making vs. the standard of care may help define the clinical role of prediction models [16].
9. *Self-fulfilling prophecy*. The impact of the self-fulfilling prophecy can be mitigated with the following measures:
  - Where feasible, in the absence of advanced directives or a moribund status on admission, prognostication and decisions on withdrawal of life-sustaining therapy should be delayed for an appropriate length of time to allow for resolution of confounders of the clinical examination, anticipated evolution of illness, and completion of appropriate diagnostic imaging.
  - Clinicians should be blinded to predictors that are not a routine part of clinical care. Assessors of outcome should also be blinded to the relevant predictors in prospective studies.

### Future Directions

Since no single variable is a reliable predictor of outcome following AIS, clinical prediction models incorporating multiple variables offer the most promising tool for prognostication of outcome. It is likely that artificial intelligence and machine learning will facilitate the future development of prediction models [165–168], and should use large, diverse datasets for training to minimize bias. Advanced imaging modalities such as diffusion tensor imaging and white matter tractography may provide insights into structural changes that correlate with impairment in various domains [169, 170], while functional MRI may help explain mechanisms of recovery [171, 172]. Functional imaging techniques may also provide valuable insights into anatomical correlates of recovery. Incorporation of genomic data and real-time physiological monitoring might enhance predictive accuracy [173, 174].

## Conclusions

These guidelines provide broad principles of neuroprognostication for patients with AIS admitted to the ICU and address the reliability of individual variables as well as widely studied prediction models in the specific context of counseling patients and families. These recommendations align clinical practice with evidence-based prognostication and optimize patient care through the adoption of good-practice recommendations.

## Supplementary Information

Below is the link to the electronic supplementary material.

Supplementary file1 (DOCX 345 KB)
