# Supplementary material for: Guidelines for Neuroprognostication in Critically ill Adults with Acute Ischemic Stroke
Source: Neurocrit Care. 2026 Apr 6;44(3):745–69. doi: 10.1007/s12028-026-02486-3 (PMC13249629; doi:10.1007/s12028-026-02486-3)
Supplement: Supplementary file 1 — Supplementary file1 (DOCX 345 KB) [file 12028_2026_2486_MOESM1_ESM.docx]

**Supplementary appendix 1**

**Neurocritical Care Society (NCS) and Deutsche Gesellschaft für Neurointensivmedizin (DGNI) Neuroprognostication guidelines: Methodology**

**Scope, purpose, and target audience**

The **scope** of these Grading of Recommendations Assessment, Development and Evaluation (GRADE) guidelines is the prognostication of neurological outcome in adults with AIS who have received standard-of-care treatment. Standard of care treatment includes, but is not limited to, revascularization therapy in appropriately selected individuals. The primary focus of this document is on patients who require intensive care unit (ICU) admission for management of the stroke itself, including post-procedural care following revascularization therapy. These guidelines are therefore not focused on patients with mild ischemic stroke or transient ischemic attack, who typically do not require ICU admission, and whose prognosis for long-term neurological recovery from the index event is generally good. The recommendations in these guidelines are focused on early neuroprognostication, particularly in the ICU. Patients admitted to inpatient rehabilitation units, or otherwise receiving post-discharge rehabilitation services, are outside the scope of this document. The **purpose** of these guidelines is to provide broad principles of neuroprognostication and evidence-based recommendations on the reliability of individual predictors of neurological outcome and clinical prediction models to assist clinicians in formulating an accurate prognosis that supports communication with patients/ surrogates, and assist with shared clinical decision making. A patient’s prognosis may serve as the basis for shared decision making on the use of some therapeutic interventions, such as decompressive craniectomy. However, these guidelines do not provide recommendations on clinical indications for therapeutic interventions. The **target audience** consists of clinicians responsible for counseling critically-ill AIS patients and/ or their surrogates.

**How to use these guidelines**

These guidelines provide recommendations on the reliability of selected individual clinical variables and prediction models (both hereafter referred to as “predictors”). These predictors have been categorized as reliable, moderately reliable, or not reliable. We based this categorization on the GRADE criteria supporting the use of the predictor in the context of counseling, as well as effect size. This categorization led to the recommendation to either use or not use the predictor to formulate a prognosis, caveats to the use of the predictor, and suggested language during counseling (**Predictor categorization tables 1 & 2,** in this supplementary document, below).

A key distinction exists between a *reliable* predictor of outcome in the context of counseling surrogates of patients requiring neurocritical care and an *independent* predictor of outcome. An independent predictor fulfills one criterion—a statistically significant association with the outcome of interest in an appropriately conducted multivariate analysis. In clinical practice, independent predictors of outcome may be used in risk stratification, selection of patients for targeted treatment (such as chemotherapy regimens for cancer), or as building blocks of clinical prediction models.[1, 2] A reliable predictor in the context of counseling patients requiring neurocritical care or their family members must be independent, but also fulfil other criteria as described in the “Effect size (Predictor accuracy)” and “Evidence to Recommendation (EtoR)” sections.

**Reliable** predictors, for the purposes of these guidelines, may be used to formulate a prognosis when the appropriate clinical context is present in the absence of potential confounders. These are predictors with clear, actionable thresholds or clinical/radiographic definitions and a low rate of error in prediction of poor outcomes, with at least moderate certainty in the body of evidence using GRADE criteria. When the prognosis is formulated on the basis of one or more reliable predictors, the clinician may describe the outcome as “very likely” during counseling. Given the inherent limitations in neuroprognostication research, the clinician must nevertheless acknowledge the presence of uncertainty—albeit low—in the prognosis.

**Moderately reliable** predictors may be used for prognostication *only* when additional reliable or moderately reliable predictors are present, in addition to the appropriate clinical context as specified above. These are also predictors with clear, actionable thresholds or clinical/radiographic definitions and a low rate of error in prediction of poor outcomes, but with lower certainty in the body of evidence using GRADE criteria, typically as a result of smaller (imprecision) and/ or fewer studies. When the prognosis is formulated on the basis of multiple moderately reliable predictors, the clinician may describe the outcome as “likely” during counseling, but must acknowledge “substantial” uncertainty in the prognosis.

While the panelists recognize that those predictors that do not meet the criteria to be described as reliable or moderately reliable are often used by clinicians in formulating their subjective impressions of prognosis, they have nevertheless been deemed **not reliable** for the purposes of these guidelines and cannot be formally recommended for prognostication on their own. Variables deemed not reliable however may be a component of reliable or moderately reliable prediction models.

**Guideline panel**

The NCS and DGNI recruited 20 professionals in neurocritical care, nursing, and pharmacy to create a panel of experts for the neurocritical care condition considered; including a NCS-assigned GRADE methodologist in 2018. The guideline panel consisted of two to four content experts who served as the primary authors for each disease, an affected patient or family member who served as the public representative, and the content experts of the other seven disease states which included the two chairs of the entire neuroprognostication guidelines. The inclusion of neurointensivists, neurosurgeons, advanced practice providers, pharmacists, and a neuropalliative care expert on the guideline panel created a multidisciplinary collaboration. A statistician-epidemiologist with expertise in neuroprognostication and medical decision making served as a consultant. The panel convened in monthly video-conferences, with in-person meetings at two consecutive annual meetings of the NCS and one annual meeting of DGNI. Additional small group video and telephone meetings occurred ad hoc among content experts, the chairs, the GRADE expert, and the public representative. Recommendations were voted on using online survey software (Google Forms, Google Inc., Mountain View, California, United States). Panelists were required to disclose all potential conflicts of interest prior to participation. Potential conflicts were reviewed by the co-chairs of the panel, as well as the NCS guidelines committee, and managed in accordance with policies of NCS.

**Selection of guideline questions**

An initial Patient, Intervention, Comparison, Outcome and Time (PICOTS) question was framed to help identify candidate predictors: *“What are the reliable predictors (prognostic factors, variables, tests, scores, and multivariable models), prior to and on admission as well as during the hospital course to predict patient outcome at different follow up time points following each specific disease?”*. A professional librarian executed a comprehensive literature search using search criteria appropriate to this initial question. Since this search was expected to generate a large volume of articles, additional criteria were used to select studies with a focus on reliability of predictors and appropriate outcomes. These criteria are described below in the “systematic review methodology” section. The content experts reviewed the full-text articles that fulfilled selection criteria. . Candidate predictors and prediction models were considered "clinically relevant" if, in the subjective opinion of the content experts and guideline chairs, the predictor or components of the prediction models were

a) accessible to clinicians (universal availability of predictors was not required); and

b) likely to be considered by clinicians while formulating a neurological prognosis.

An appropriate body of literature was considered present for any **predictor** evaluated in published studies that included (1) a minimum of 100 adult subjects and (2) an appropriate multivariate analysis which includes the predictor of interest, patient age, severity of neurological illness, and universally accepted parameters associated with the outcome as explanatory variables. The sample size was selected to ensure a broad overview of the appropriate literature, although the appropriate sample size for multivariate analyses of predictors of outcome in AIS has not been well established.[3, 4] For **biomarkers** an appropriate body of literature was considered present if at least one external validation study in addition to the initial report on discovery of biomarker as an independent predictor was found. For **clinical prediction models**, an appropriate body of literature was considered present if at least one external validation study in addition to the initial report on development of the model was found. For example, models such as the MRPREDICTS@24H postprocedural outcome prediction model, while otherwise promising, were not included as candidate models because of a lack of external validation studies beyond the original publication, at the time of last literature review.[5]

Based on these criteria, the following candidate predictors were selected:

Clinical variables:

1. Age
2. National Institute of Health Stroke Scale (NIHSS) on admission.
3. Blood glucose
4. Cerebral collateral circulation status.
5. Hypertension
6. Infarct size
7. History of previous stroke
8. Revascularization status
9. Early Neurological Improvement (ENI)

Clinical prediction models:

1. Acute Stroke Registry and Analysis of Lausanne (ASTRAL) score
2. Dense Artery, mRS, Age, Glucose, Onset-to-Treatment, and NIHSS (DRAGON) score
3. Ischemic stroke predictive risk score (iScore)
4. Totaled Health Risks in Vascular Events (THRIVE) score.

**PICOTS question**

The Population/ Intervention/ Comparator/ Outcome/ Time frame/ Setting (PICOTS) question was then framed for the specific candidate predictors as follows:

"When counseling critically-ill adults with acute ischemic stroke or their surrogates, should <predictor or prediction model, with time of assessment if appropriate> be considered a reliable predictor of <outcome>?”

**Selection of outcomes**

The topic experts and the patient and family representative rated outcomes on the GRADE 1-9 scale based on their perceived importance. Outcomes with a median rating 7-9 were considered “critical”. Outcomes were rated as follows:

1. Functional outcome- 9
2. Mortality- 8
3. Quality of life- 8
4. Cognitive outcome- 7
5. Depression- 7
6. Return to work- 6

While quality of life, cognitive function, and depression were critical outcomes, the body of evidence with these outcome measures was insufficient to support any recommendations. In addition, the risk of bias from the self-fulfilling prophecy within the existing body of literature on predictors of mortality was considered too high to recommend any single clinical variable to predict death *when all available means of life support are used, indefinitely and without limitation*.[6] A brief summary of the GRADE evidence profile and summary of findings for predictors of mortality is in **Supplementary Table 1**, while a summary of recommendations for predictors of mortality is in **Supplementary Table 2**. The recommendations within these guidelines are primarily focused on the prediction of functional outcome.

**Functional outcome assessment**

The majority of studies used the Modified Rankin Scale (mRS), which ranges from 0 (asymptomatic) to 6 (death), as a measure of functional outcome.[7, 8] The definition of a “good” functional outcome has varied across the literature. Clinical trials of revascularization, with either intravenous fibrinolytics or endovascular techniques, have typically used a mRS of 2 or lower as the primary outcome measure,[9] with the patient’s ability to independently manage their own affairs as the primary determinant. An mRS<=2 requires independence in all basic activities of daily living within the home, but also the ability to manage finances and procure groceries, while other pre-stroke activities (including leisure and employment) may no longer be possible. Clinical trials focused on patients with greater stroke severity - such as trials of hemicraniectomy- have used a different threshold to define “good” functional outcome, in recognition of the lower probability of achieving complete functional independence following a more severe stroke.[10] A mRS of 3 or lower is therefore frequently used as the definition of a “good” outcome in this context, with the ability to ambulate independently, with or without an assistive device, as the primary determinant. The choice of these dichotomous outcome measures has not necessarily reflected the preferences of AIS patients or their families, who may define a “good” functional outcome differently. In the SETPOINT2 clinical trial of early tracheostomy following stroke- entirely focused on critically-ill stroke patients- the primary outcome measure was based on structured conversations with patients and families, who expressed a strong preference for a mRS<=4 threshold of a “good” outcome.[11] The primary determinant of this threshold is simply the ability to sit up unassisted in bed, and remain unsupervised for limited periods of time.[12] Recognizing the limitations of dichotomization of outcome, more recent studies have used an ordinal or shift analysis, which recognizes that patients and families may value any meaningful improvement in functional outcome grades beyond arbitrary clinician-selected thresholds.[13, 14] Since some transitions across the mRS are considered more meaningful to patients than others- such as the recovery of independent ambulation compared to resolution of minor nondisabling symptoms- utility weighting of mRS transitions in ordinal analyses has also been used in recent studies.[15] In recognition of the evolution in outcome analysis in the ischemic stroke literature, an inclusive definition of good functional outcome was used for the purposes of this systematic review, encompassing all of the mRS thresholds and analyses described above. Variability in outcome definition was, therefore, an inherent limitation of the review. Other acceptable measures of functional outcome in the literature included the Barthel Index, Glasgow Outcome Scale (GOS), Functional Independence Measure (FIM), and Lawton’s Activities of Daily Living (ADL) scale.

**Systematic review methodology**

Since the literature on prognosis was expected to be heterogenous, a narrative systematic review was performed. As described earlier, the initial librarian search string was appropriate to the question *“What are the reliable predictors (prognostic factors, variables, tests, scores, and multivariable models), prior to and on admission as well as during the hospital course to predict patient outcome at different follow up time points following each specific disease ?”*, to identify candidate predictors. The initial librarian search was performed February 20, 2019, and encompassed the period from 1946 to the search date. Databases searched included MEDLINE via PubMed, EMBASE, Web of Science and the Cochrane Database of Systematic Reviews. Updated searches were performed August 1, 2022, and February 5, 2024. Screening of articles was completed using DistillerSR software (Evidence Partners, Ottawa, Canada).

Abstract screening (Level I) was performed with the question “Does this study address prognostication in the focus disease (Yes/ No)?”. Pediatric (<16 years) and animal studies were excluded, as were studies evaluating the efficacy of a therapeutic intervention.

Full text screening (Level II) was performed with the following exclusion criteria:

- Minimum sample size (≥100)
- Studies focused exclusively on TIA and/ or mild stroke
- Studies focused on a highly selected subgroup (such as periprocedural stroke),
- Studies focused entirely on genetic polymorphism,
- Studies that include multiple disease states and without an adequate sample size and separate analysis of the disease of interest,
- Studies of an intervention,
- Studies with neuroimaging not consistent with contemporary standards used to confirm the diagnosis,
- Studies that did not include one of the selected outcomes for the specific disease,
- Studies of predictors not established as independent with appropriate multivariate analysis,
- Studies of clinical prediction models that did not report model discrimination,
- Studies of laboratory biomarkers were included only if the biomarker was considered clinically relevant, and had been evaluated in more than one published study that met other criteria.

Data extraction and assessment for risk of bias (RoB) and was then performed only for studies that addressed the PICOTS question for each selected candidate predictor AND fulfilled full-text selection criteria. The Quality in Prognostic Studies (QUIPS) RoB instrument was used to evaluate studies of individual prognostic variables,[16] and the Prediction model Risk Of Bias ASsessment Tool (PROBAST) instrument used to evaluate studies of clinical prediction models.[17, 18] In addition to the standard domains of these RoB instruments, studies were evaluated for the risk of bias related to the self-filling prophecy with an additional domain that included three questions- whether a treatment suspension policy was used in the study, whether clinicians were blinded to the predictor and whether the predictor was systematically utilized by clinicians for prognostication during the time period of the study. A content expert evaluated each study for RoB, with secondary review by another content expert. Disagreements between reviewers were resolved through discussion. Articles written or co-written by one of the authors were independently assessed for risk of bias by another author and a methodologist. Where disagreements could not be resolved, adjudication was performed by the methodologist. Following assessment of risk of bias in each domain, an overall risk of bias- *high, moderate or low*- was assigned to each study.

Following data extraction and assessment of risk of bias of individual studies, a GRADE evidence profile with summary of findings table was constructed. Statistical measures of effect size varied across studies and were recorded in narrative form in the summary of findings. Decisions to downgrade the body of evidence for risk were based on review of risk of bias across all individual studies for the PICOTS question. The body of evidence was downgraded once for a moderate overall RoB across all individual studies and twice for a high overall RoB. The body of evidence for a specific predictor was downgraded for inconsistency only when the results of studies with approximately equal risk of bias were in conflict, with some studies suggesting the predictor was reliable and other studies showing a lack of statistical significance or a high rate of error. The evidence was downgraded for indirectness when population, predictor, outcome, or time of assessment varied significantly from the specifications of the PICOTS question. For example, since most studies of predictors following AIS were not limited to critically-ill patients admitted to the ICU, a downgrade for indirectness was required for the body of evidence for most PICOTS questions. Downgrading of the evidence for imprecision was applied when the estimated confidence intervals were thought to be excessive for the clinical question. Two downgrades each were also permitted for inconsistency, indirectness and imprecision but were rarely necessary. Publication bias could not be meaningfully evaluated, and the GRADE reasons to upgrade were not thought to be applicable to this body of evidence. Judgments on risk of bias, indirectness, inconsistency and imprecision were inherently subjective and focused on the respective prognostication question.

**Effect size (Predictor accuracy)**

Statistical measures of effect size varied across studies and were recorded in narrative form in the summary of findings. Predictor accuracy is often described using measures such as the odds ratio (OR), which measures the *relative* probability of the outcome when the predictor is present, compared to the probability of the outcome in the absence of the predictor. In the context of neuroprognostication, predictors of good and poor outcomes are considered. However, the absolute probability of the outcome when the predictor is present is also important. For example, an older patient may be statistically more likely to suffer a poor outcome than a younger patient, but a large proportion of older individuals may nevertheless have a good outcome.[19] While surrogates of neurocritical care patients may be interested in factors that increase the probability of poor outcome, the absolute probability of a poor outcome is most likely used to make decisions on escalation or withdrawal of treatment measures. The performance of a clinical prediction model is evaluated using its ability to discriminate binary (good vs poor) outcomes, with measures such as the c-statistic or area under the receiver operating characteristic curve (AUC). Another important measure is model calibration, or the ability to correctly specify the probability of an outcome. Model calibration is typically reported as a goodness of fit, often using the Hosmer-Lemeshow test, or with a calibration curve, slope or intercept.[20]

**Evidence to recommendation criteria**

The GRADE Evidence-to-Recommendation criteria encompassed the overall quality of evidence, balance between desirable and undesirable outcomes, confidence in values and preferences, and resource use. Common principles were established for each disease state by the guideline panel in consultation with the patient or family representative, when considering these criteria.

**1. Quality of Evidence/ Certainty in the evidence and effect size**: For the purposes of these guidelines, predictors described as “reliable” have both a higher overall certainty in the evidence and greater effect size than “moderately reliable” predictors **(Table 1)**. Predictors of poor outcome were required to demonstrate a low false positive rate, <3% for reliable predictors (with the upper limit of the 95% confidence interval <10%) and <5% (imprecision allowed) for moderately reliable predictors. This was because an inaccurate prediction of poor outcome may lead to inappropriate WLST. The acceptable threshold for predictors of good outcome was considerably lower, since an inappropriate prediction of good outcome is not expected to lead to a potentially irreversible decision such as WLST (see “Balance of desirable and undesirable consequences below”). A positive predictive value >60% was considered acceptable, with acknowledgment of uncertainty and language commensurate to the likelihood of recovery (see “Balance of desirable and undesirable consequences” below). Reliable clinical prediction models were required to demonstrate very good discrimination with an area under the curve (AUC) >0.8, without inconsistency, imprecision, indirectness or evidence of miscalibration in the body of evidence. Moderately reliable clinical prediction models were required to demonstrate an AUC >0.7, without inconsistency.

**2. Balance of desirable and undesirable consequences:** Neuroprognostication in the critically-ill is often focused on the prediction of poor outcomes. An accurate prediction of poor outcome is expected to result in grief, a sense of loss and anxiety about the future. However, accurate prediction of a poor outcome is overall desirable, since it allows surrogates and the clinical team to align goals of care to the perceived wishes of the AIS patient, through the process of shared decision making. Potential benefits to the family and surrogates in this situation include greater certainty and decreased decisional conflict in making patient value-congruent decisions, a sense of closure, and satisfaction from respecting the patient’s wishes. An *inaccurate* prediction of a poor outcome (i.e, a false-positive prediction of poor outcome), however, may lead to WLST in an AIS patient who would otherwise have made a recovery to functional independence. Since WLST almost always leads to death in critically-ill patients on life-sustaining therapy, the undesirable consequences of an inaccurate prediction of poor outcome were thought to greatly outweigh the desirable consequences, unless certainty in the accuracy of the predictor or prediction model was high. *The concern for inappropriate WLST was central in the panel’s decision to recommend, or not recommend, prognostication and counseling based on the selected predictors of poor outcome*.

The prediction of *good* outcome, however, involves a different balance of desirable and undesirable consequences. Patients and surrogates are likely to feel encouraged and hopeful, and continue life-sustaining treatment, which was overall considered desirable. Anticipation of a good long-term (defined as 3 or more months from the stroke in these guidelines) outcome may ease the psychological and physical burdens of critical illness and short-term debility, both for patients and surrogates. Anticipation of a good long term outcome was also thought to be desirable for patient motivation during rehabilitation, which may be an independent predictor of good functional outcome following AIS.[21] An undesirable consequence of inaccurate prediction of a good outcome is disappointment and disillusionment among both patients and surrogates, which may adversely impact motivation. An inaccurate prediction of good outcome may also lead to prolongation of suffering among patients and surrogates. An additional undesirable consequence was thought to be discordance in the understanding of “good” outcome between clinicians and patients/ surrogates. While clinicians generally consider a good outcome following AIS to be a return to functional independence, patients and surrogates may perceive psychological or cognitive impairment, and limitations in employment or leisure activities to represent a poor outcome despite a return to functional independence. Conversely, a physician’s perceived poor outcome might be seen by patients and families as acceptable.[11, 22, 23] Overall, the panel considered a prediction of good outcome to be desirable, using language commensurate to the actual likelihood of recovery, such as “more likely/ more often than not”, or “highly likely, but not certain”. Clarity during counseling of the specific “good” outcome under discussion, such as a recovery of ambulation, recovery of swallow function, or the ability to perform basic activities of daily living, was also thought to be important.

**3. Values and preferences:** The panel agreed that most individuals, as well as their families and surrogates, would consider an inaccurate prediction of poor outcome that led to the death of a patient who might otherwise have had a reasonable recovery to be more undesirable than a prolonged period of uncertainty in the outcome. Therefore, a high certainty in the evidence of predictor or prediction model accuracy was necessary to recommend consideration when counseling families and surrogates on prognosis in this context. Patient and family representatives on the panel, however, stated a preference for the clinical team communicating any expectations of recovery of function.

**4. Resource use:** The predictors evaluated in the systematic review are integral to the delivery of clinical care following AIS, and do not require additional resources. However, an important consideration was the substantial financial burden that may be imposed on patients, surrogates and the healthcare system with continuation of supportive measures based on an inaccurate prediction of good outcome- particularly when the patient’s outcome is inconsistent with their desired quality of life.[24-26] An accurate prediction of *poor* outcome will lead to better alignment of goals of care with the patient's wishes and avoid the extended use of resources, over days to years. The use of resources was therefore thought to favor consideration of a predictor or prediction model during prognostication, contingent on confidence in its predictive accuracy. In situations where goals of care have been established and are unlikely to change, however, resource utilization involved with performance of the test should likely be considered, and expensive tests not expected to alter the treatment plan should be avoided.

**Good practice statements**

In accordance with recommendations of the GRADE network these statements were considered by the panel to be actionable, supported by indirect evidence where appropriate, and essential to guide the practice of neuroprognostication.[27] The good clinical practice reflected in these statements were considered by the panel to be unequivocally beneficial.

**Supplementary appendix 2**

**Librarian search string:**

Database: All Ovid Medline <1946 - present>

Search Strategy:

--------------------------------------------------------------------------------

1 exp Brain Ischemia/ (100263)

2 ischemic.ti,ab. (188652)

3 ischemia.ti,ab. (164765)

4 ischaemic.ti,ab. (36413)

5 ischaemia.ti,ab. (26723)

6 2 or 3 or 4 or 5 (348858)

7 APOPLEXY.ti,ab. (2922)

8 cerebrovascular accident.ti,ab. (3937)

9 cerebrovascular disorder*.ti,ab. (2027)

10 (infarction adj10 (brain or cerebral)).mp. [mp=title, abstract, original title, name of substance word, subject heading word, floating sub-heading word, keyword heading word, protocol supplementary concept word, rare disease supplementary concept word, unique identifier, synonyms] (44483)

11 Intracranial Arteriosclerosis.ti,ab. (7)

12 Intracranial.ti,ab. (100585)

13 stroke/ (88198)

14 stroke.ti,ab. (212557)

15 7 or 8 or 9 or 10 or 11 or 12 or 13 or 14 (349358)

16 6 and 15 (84733)

17 1 or 16 (144763)

18 Disease Progression/ (145175)

19 "Predictive Value of Tests"/ (187429)

20 exp PROGNOSIS/ (1478133)

21 prognostic.tw. (257061)

22 18 or 19 or 20 or 21 (1793342)

23 17 and 22 (25000)

24 outcome$.mp. (2175375)

25 end point$.mp. (57259)

26 (core adj3 set).mp. (3768)

27 treatment emergent problem$.mp. (1)

28 exp outcome Assessment Health Care/ (977764)

29 Treatment Outcome/ (881567)

30 Quality of Life/ (170981)

31 24 or 25 or 26 or 27 or 28 or 29 or 30 (2333265)

32 23 and 31 (18550)

33 "Analysis of Variance"/ [includes Multivariate Analysis] (212627)

34 outcome variable.mp. (6336)

35 Multivariate.tw. (292269)

36 variable*.ti,ab. (724132)

37 variance*.ti,ab. (168955)

38 Data Interpretation, Statistical/ (54498)

39 statistic*.tw. (970581)

40 sn.fs. [statistics and numerical data as a floating subheading] (721852)

41 33 or 34 or 35 or 36 or 37 or 38 or 39 or 40 (2664928)

42 32 and 41 (4720)

43 exp cohort studies/ [includes: follow-up studies/, longitudinal studies/, prospective studies/, retrospective studies/, controlled before-after studies/, cross-sectional studies/, or historically controlled study/] (1816093)

44 (Follow-up or longitudinal or prospective or retrospective or before-after or cross-sectional or controlled).tw. (2584892)

45 (comparative study or evaluation study or meta analysis).pt. (1907014)

46 43 or 44 or 45 (4825456)

47 42 and 46 (3669)

48 pediatrics/ (50537)

49 animals/ not (humans/ and animals/) (4505965)

50 48 or 49 (4556432)

51 47 not 50 (3647)

52 limit 51 to english language (3526)

***************************

**Supplementary Appendix 3**

**Hyperglycemia and Poor Cerebral Collateral Circulation Status as predictors of Functional Outcome**

PICO Question #8: "When counseling critically-ill adults with AIS or their surrogates, should admission hyperglycemia alone be considered a reliable predictor of poor functional outcome assessed at 3 months or later?"

Description of the predictor: In ischemic stroke research, hyperglycemia is typically identified by glucose levels ranging from > 6.1 to 8.0 mmol/L (approximately 110 to 144 mg/dL), with variations in thresholds across studies. The timing of glucose measurement is also not consistent, ranging from a single admission measurement to multiple assessments within the hospital stay.

Recommendation: When counseling critically-ill patients with AIS or their surrogates, we suggest admission hyperglycemia alone not be considered a reliable predictor of poor functional outcome assessed at 3 months or later *(weak recommendation; very low quality evidence).*

Rationale:

The body of evidence was downgraded once each for inconsistency, indirectness, imprecision and for risk of bias in the domains of study participation, attrition, prognostic factor measurement, outcome measurement, confounding, statistical analysis, and self-fulfilling prophecy. Given the many factors influencing blood glucose, variability in measurement and reporting, and incompleteness in patient and variable description, blood glucose alone cannot serve as the basis for prognostication.

PICO Question #9: "When counseling critically-ill patients with AIS or their surrogates, should poor cerebral collateral circulation status alone be considered a reliable predictor of poor functional outcome assessed at 3 months or later?"

Description of the predictor**:** A robust cerebral collateral circulation will minimize ischemic injury and improve outcomes by sustaining perfusion to at-risk territories. Several collateral scoring systems have been developed to evaluate cerebral collateral status in ischemic stroke. The ASITN/SIR system uses digital subtraction angiography (DSA) to gauge collateral flow.[28] The Tan and Miteff scores employ CT and MR angiograms, respectively.[29, 30] Maas et al. described a score evaluating collaterals through single-phase CT angiograms,[31] while the multiphase CTA score provides an analysis of dynamic contrast enhancement across multiple imaging phases.[32]

Recommendation: When counseling critically-ill patients with AIS or their surrogates, we suggest poor cerebral collateral circulation status alone not be considered a reliable predictor of poor functional outcome assessed at 3 months or later *(weak recommendation; low quality evidence).*

Rationale: The body of evidence was downgraded once each for imprecision and for risk of bias in the domains of study participation, attrition, prognostic factor measurement, outcome measurement, confounding, statistical analysis, and self-fulfilling prophecy. Patients with poor collateral status may nevertheless achieve an excellent outcome following successful revascularization, and/ or in the presence of other favorable factors.[33, 34] While collateral status is an important factor in minimizing injury and improving outcomes following AIS,[35-38] it cannot serve as the basis for prognostication while counseling patients and families.

**REFERENCES:**

1. Hemingway, H., Croft, P., Perel, P., et al. Prognosis research strategy (PROGRESS) 1: a framework for researching clinical outcomes*.* BMJ 2013;346:e5595.

2. Riley, R.D., Hayden, J.A., Steyerberg, E.W., et al. Prognosis Research Strategy (PROGRESS) 2: prognostic factor research*.* PLoS Med 2013;10(2):e1001380.

3. Riley, R.D., Ensor, J., Snell, K.I.E., et al. Calculating the sample size required for developing a clinical prediction model*.* BMJ 2020;368:m441.

4. Riley, R.D., Snell, K.I., Ensor, J., et al. Minimum sample size for developing a multivariable prediction model: PART II - binary and time-to-event outcomes*.* Stat Med 2019;38(7):1276-96.

5. Chalos, V., Venema, E., Mulder, M., et al. Development and Validation of a Postprocedural Model to Predict Outcome After Endovascular Treatment for Ischemic Stroke*.* JAMA Neurol 2023;80(9):940-8.

6. Teixeira, F.J.P., Ahmad, B., Gibatova, V., et al. Do Neuroprognostic Studies Account for Self-Fulfilling Prophecy Bias in Their Methodology? The SPIN Protocol for a Systematic Review*.* Crit Care Explor 2023;5(7):e0943.

7. Rankin, J. Cerebral vascular accidents in patients over the age of 60. II. Prognosis*.* Scott Med J 1957;2(5):200-15.

8. Wilson, J.T., Hareendran, A., Hendry, A., et al. Reliability of the modified Rankin Scale across multiple raters: benefits of a structured interview*.* Stroke 2005;36(4):777-81.

9. Goyal, M., Menon, B.K., van Zwam, W.H., et al. Endovascular thrombectomy after large-vessel ischaemic stroke: a meta-analysis of individual patient data from five randomised trials*.* Lancet 2016;387(10029):1723-31.

10. Reinink, H., Juttler, E., Hacke, W., et al. Surgical Decompression for Space-Occupying Hemispheric Infarction: A Systematic Review and Individual Patient Meta-analysis of Randomized Clinical Trials*.* JAMA Neurol 2021;78(2):208-16.

11. Bosel, J., Niesen, W.D., Salih, F., et al. Effect of Early vs Standard Approach to Tracheostomy on Functional Outcome at 6 Months Among Patients With Severe Stroke Receiving Mechanical Ventilation: The SETPOINT2 Randomized Clinical Trial*.* JAMA 2022;327(19):1899-909.

12. Bruno, A., Akinwuntan, A.E., Lin, C., et al. Simplified modified rankin scale questionnaire: reproducibility over the telephone and validation with quality of life*.* Stroke 2011;42(8):2276-9.

13. Ganesh, A., Luengo-Fernandez, R., Wharton, R.M., Rothwell, P.M.Oxford Vascular, S. Ordinal vs dichotomous analyses of modified Rankin Scale, 5-year outcome, and cost of stroke*.* Neurology 2018;91(21):e1951-e60.

14. Saver, J.L. Novel end point analytic techniques and interpreting shifts across the entire range of outcome scales in acute stroke trials*.* Stroke 2007;38(11):3055-62.

15. Chaisinanunkul, N., Adeoye, O., Lewis, R.J., et al. Adopting a Patient-Centered Approach to Primary Outcome Analysis of Acute Stroke Trials Using a Utility-Weighted Modified Rankin Scale*.* Stroke 2015;46(8):2238-43.

16. Hayden, J.A., van der Windt, D.A., Cartwright, J.L., Cote, P.Bombardier, C. Assessing bias in studies of prognostic factors*.* Ann Intern Med 2013;158(4):280-6.

17. Moons, K.G.M., Wolff, R.F., Riley, R.D., et al. PROBAST: A Tool to Assess Risk of Bias and Applicability of Prediction Model Studies: Explanation and Elaboration*.* Ann Intern Med 2019;170(1):W1-W33.

18. Wolff, R.F., Moons, K.G.M., Riley, R.D., et al. PROBAST: A Tool to Assess the Risk of Bias and Applicability of Prediction Model Studies*.* Ann Intern Med 2019;170(1):51-8.

19. Gattellari, M., Goumas, C., Garden, F.Worthington, J.M. Relative survival after transient ischaemic attack: results from the Program of Research Informing Stroke Management (PRISM) study*.* Stroke 2012;43(1):79-85.

20. Van Calster, B., McLernon, D.J., van Smeden, M., et al. Calibration: the Achilles heel of predictive analytics*.* BMC Med 2019;17(1):230.

21. Wang, Y.H., Yang, Y.R., Pan, P.J.Wang, R.Y. Modeling factors predictive of functional improvement following acute stroke*.* J Chin Med Assoc 2014;77(9):469-76.

22. Neugebauer, H., Creutzfeldt, C.J., Hemphill, J.C., 3rd, Heuschmann, P.U.Juttler, E. DESTINY-S: attitudes of physicians toward disability and treatment in malignant MCA infarction*.* Neurocrit Care 2014;21(1):27-34.

23. Neugebauer, H., Schnabl, M., Lule, D., et al. Attitudes of Patients and Relatives Toward Disability and Treatment in Malignant MCA Infarction*.* Neurocrit Care 2017;26(2):311-8.

24. Mayer, S.A., Copeland, D., Bernardini, G.L., et al. Cost and outcome of mechanical ventilation for life-threatening stroke*.* Stroke 2000;31(10):2346-53.

25. Raj, R., Bendel, S., Reinikainen, M., et al. Costs, outcome and cost-effectiveness of neurocritical care: a multi-center observational study*.* Crit Care 2018;22(1):225.

26. Rochmah, T.N., Rahmawati, I.T., Dahlui, M., Budiarto, W.Bilqis, N. Economic Burden of Stroke Disease: A Systematic Review*.* Int J Environ Res Public Health 2021;18(14).

27. Dewidar, O., Lotfi, T., Langendam, M.W., et al. Good or best practice statements: proposal for the operationalisation and implementation of GRADE guidance*.* BMJ Evid Based Med 2022.

28. Higashida, R.T., Furlan, A.J., Roberts, H., et al. Trial design and reporting standards for intra-arterial cerebral thrombolysis for acute ischemic stroke*.* Stroke 2003;34(8):e109-37.

29. Miteff, F., Levi, C.R., Bateman, G.A., et al. The independent predictive utility of computed tomography angiographic collateral status in acute ischaemic stroke*.* Brain 2009;132(Pt 8):2231-8.

30. Tan, I.Y., Demchuk, A.M., Hopyan, J., et al. CT angiography clot burden score and collateral score: correlation with clinical and radiologic outcomes in acute middle cerebral artery infarct*.* AJNR Am J Neuroradiol 2009;30(3):525-31.

31. Maas, M.B., Lev, M.H., Ay, H., et al. Collateral vessels on CT angiography predict outcome in acute ischemic stroke*.* Stroke 2009;40(9):3001-5.

32. Menon, B.K., d'Esterre, C.D., Qazi, E.M., et al. Multiphase CT Angiography: A New Tool for the Imaging Triage of Patients with Acute Ischemic Stroke*.* Radiology 2015;275(2):510-20.

33. Al-Dasuqi, K., Payabvash, S., Torres-Flores, G.A., et al. Effects of Collateral Status on Infarct Distribution Following Endovascular Therapy in Large Vessel Occlusion Stroke*.* Stroke 2020;51(9):e193-e202.

34. Yabalak, A., Ogun, M.N., Onalan, A., et al. Evaluation of the relationship between computed tomography angiography collateral scores and clinical outcome*.* Arq Neuropsiquiatr 2024;82(3):1-7.

35. Piedade, G.S., Schirmer, C.M., Goren, O., et al. Cerebral Collateral Circulation: A Review in the Context of Ischemic Stroke and Mechanical Thrombectomy*.* World Neurosurg 2019;122:33-42.

36. Jung, S., Gilgen, M., Slotboom, J., et al. Factors that determine penumbral tissue loss in acute ischaemic stroke*.* Brain 2013;136(Pt 12):3554-60.

37. Marks, M.P., Lansberg, M.G., Mlynash, M., et al. Effect of collateral blood flow on patients undergoing endovascular therapy for acute ischemic stroke*.* Stroke 2014;45(4):1035-9.

38. Puhr-Westerheide, D., Tiedt, S., Rotkopf, L.T., et al. Clinical and Imaging Parameters Associated With Hyperacute Infarction Growth in Large Vessel Occlusion Stroke*.* Stroke 2019;50(10):2799-804.

**Supplementary Table 1**

**GRADE Evidence Profile/ Summary of Findings table for Mortality: Neuroprognostication- Acute Ischemic Stroke**

| **Outcome** | **Predictor** | **Quality of Evidence** | | | | | **Summary of Findings (Narrative of effect size)** |
| --- | --- | --- | --- | --- | --- | --- | --- |
|  |  | **Risk of Bias** | **Inconsistency** | **Indirectness** | **Imprecision** | **Quality of Evidence- Summary** |  |
| Mortality | Age | ↓↓ | ↓ | ↓ |  | Very low | OR: 0.18 to 6.64  HR: 1.03 to 8.01  Beta: -1.75 to 0.185 |
| Mortality | NIHSS | ↓↓ | ↓ | ↓ | ↓ | Very Low |  |
| Mortality | Blood glucose | ↓↓ | ↓ | ↓ | ↓ | Very Low | OR: 0.36 to 7.61  HR: 1.036 to 2.0  RR: 0.69 to 6.05 |
| Mortality | Cerebral collateral circulation status | ↓↓ | ↓ |  | ↓ | Very Low | OR: 0.21 to 14.13 |
| Mortality | Hemorrhagic Transformation | ↓↓ | ↓ | ↓ | ↓ | Very low | OR/HR: 1.34 to 26.48 |
| Mortality | Infarct size | ↓↓ | ↓ | ↓ | ↓ | Very low | OR: 1.96 to 2.23 |
| Mortality | Previous stroke | ↓↓ | ↓ | ↓ | ↓ | Very Low | OR: 1.23 to 3.78 |
| Mortality | ASTRAL | ↓↓ |  | ↓ |  | Very low | C-stat: 0.76 to 0.85 |
| Mortality | IScore | ↓↓ |  | ↓ |  | Very low | C-stat: 0.68 to 0.86 |

**Supplementary Table 2**

**Recommendations: Predictors of mortality in Acute Ischemic Stroke**

| **Recommendations: Clinical variables as predictors of functional outcome** |
| --- |
| 1. When counseling critically-ill adults with AIS or their surrogates, we suggest age alone not be considered a reliable predictor of mortality *(weak recommendation; very low quality evidence).* |
| 1. When counseling critically-ill adults with AIS or their surrogates, we suggest the admission NIHSS alone not be considered a reliable predictor of mortality *(weak recommendation; very low quality evidence).* |
| 1. When counseling critically-ill patients with AIS or their surrogates, we suggest admission hyperglycemia alone not be considered a reliable predictor of mortality *(weak recommendation; very low quality evidence).* |
| 1. When counseling critically-ill patients with AIS or their surrogates, we suggest collateral status alone not be considered a reliable predictor of mortality *(weak recommendation; very low quality evidence).* |
| 1. When counseling critically-ill patients with AIS or their surrogates, we suggest hemorrhagic transformation alone not be considered a reliable predictor of mortality *(weak recommendation; very low quality evidence).* |
| 1. When counseling critically-ill patients with AIS or their surrogates, we suggest infarct size alone not be considered a reliable predictor of mortality *(weak recommendation; very low quality evidence).* |
| 1. When counseling of critically-ill patients with AIS or their surrogates, we suggest a history of previous stroke alone not be considered a reliable predictor of mortality *(weak recommendation; very low quality evidence).* |
| **Recommendations: Clinical prediction models as predictors of functional outcome** |
| 1. When counseling critically-ill AIS patients or their surrogates, we suggest the ASTRAL score not be considered a reliable predictor of mortality *(weak recommendation; very low quality evidence).* |
| 1. When counseling critically-ill AIS patients or their surrogates, we suggest the IScore prediction model not be considered a reliable predictor of mortality *(weak recommendation; very low quality evidence).* |

**Supplementary Table 3:**

**INDIVIDUAL STUDIES: RISK OF BIAS AND SUMMARY OF FINDINGS**

**AGE**

| **First author last name** | **Year of publication** | **PMID** | **Timing of prognostic factor evaluation** | **Prevalence % of the predictor** | **Outcome(s)** | **Sample size for the outcome** | **Prevalence % of the outcome** | **Effect size with 95% CI as reported in the study (false positive rate, sensitivity, specificity, odds ratio, relative risk)** | **Overall risk of bias for the study** | **Overall risk of bias: comments** |
| --- | --- | --- | --- | --- | --- | --- | --- | --- | --- | --- |
| Ryu | 2017 | 28008000 | admission | Continuous variable | 90-d mRS | 5035 | 100% | OR: 1.03 (1.02-1.03); P<0.001 | Moderate | 5035 patients from 8005 initially evaluated from 2011-2012; many post-hoc analyses conducted |
| Demeestere | 2018 | 30355098 | admission | Continuous variable | 90-d mRS 0-2 | 156 | 58.9% | OR 0.97 (0.95-1); p=0.05 | High | 156 patients, retrospectively analyzed, but prospectively enrolled in the CRISP study evaluating LVO in the anterior circulation who underwent endovascular therapy and had successful reperfusion |
| Sico | 2018 | 30294472 | admission | Continuous variable | mortality, in-hospital | 3750 | 3.6% | OR 1.63 (1.26 to 2.11) | Moderate | 3750 patients from 131 Veterans Health Admin facilities with stroke in 2007; retrospectively collected and analyzed data |
| Sico | 2018 | 30294472 | admission | Continuous variable | 30-d mortality | 3750 | 7.8% | OR 1.870 (1.56 to 2.25) | Moderate | 3750 patients from 131 Veterans Health Admin facilities with stroke in 2007; retrospectively collected and analyzed data |
| Sico | 2018 | 30294472 | admission | Continuous variable | 180-d mortality | 3750 | 14.5% | OR 1.56 (1.36 to 1.78) | Moderate | 3750 patients from 131 Veterans Health Admin facilities with stroke in 2007; retrospectively collected and analyzed data |
| Sico | 2018 | 30294472 | admission | Continuous variable | 1-y mortality | 3750 | 18.8% | OR 1.47 (1.305 to 1.65) | Moderate | 3750 patients from 131 Veterans Health Admin facilities with stroke in 2007; retrospectively collected and analyzed data |
| Chen | 2018 | 30166435 | admission | Continuous variable | 90-d mRS 0-2 | 104 | 42.3% | OR 0.965 (0.926–1.004); p=0.08 | High | 104 consecutive patients with M1 occlusion ischemic stroke who could be evaluated for thrombus permeability based on conventional CTA |
| Maus | 2018 | 29946292 | admission | Continuous variable | 90-d mRS 3-6 | 392 | 60.2% | OR 1.8 (1.4-2.4) | Moderate | Retrospective analysis of 392 patients with LVO undergoing thrombectomy between 2014-2017 |
| Bentes | 2018 | 29935475 | admission | Continuous variable | mRS >2, at discharge | 151 | 65.5% | P<0.05 | Moderate | 151 prospectively identified consecutive patients with anterior circulation ischemic stroke; followed for 12 months; primary study was regarding EEG findings and association with outcomes |
| Bentes | 2018 | 29935475 | admission | Continuous variable | 1-y mRS >2 | 151 | 50.9% | P<0.05 | Moderate | 151 prospectively identified consecutive patients with anterior circulation ischemic stroke; followed for 12 months; primary study was regarding EEG findings and association with outcomes |
| Laredo | 2018 | 29934530 | admission | Continuous variable | 90-d mRS >2 | 195 | NR | P<0.05 | Moderate | 195 patients from two cohorts, primarily evaluating the association of insular involvement with outcomes; reporting of outcomes was sparce |
| Laredo | 2018 | 29934530 | admission | Continuous variable | 90-d mortality | 195 | NR | P<0.05 | Moderate | 195 patients from two cohorts, primarily evaluating the association of insular involvement with outcomes; reporting of outcomes was sparce |
| Al-Ajlan | 2018 | 29170266 | admission | Continuous variable | 90-d mRS 0-2 | 206 | 35.9% | p=0.01 | Moderate | 206 prospectively enrolled patients in the REVASCAT trial; 103 received mechanical thrombectomy for anterior circulation, LVO |
| Park | 2018 | 28624400 | admission | Continuous variable | 3-y mortality | 2069 | 18% | P<0.05 | Moderate | 2069 consecutive patients prospectively evaluated from 2002-2010 for LV mass and geometry |
| Bentes | 2017 | 29588974 | admission | Continuous variable | mRS >2, discharge | 151 | 65.6% | OR = 1.02 (0.99–1.06); p = 0.246 | Moderate | 151 prospectively identified consecutive patients with anterior circulation ischemic stroke; followed for 12 months; primary study was regarding EEG findings and association with outcomes |
| Bentes | 2017 | 29588974 | admission | Continuous variable | 1-y mRS >2 | 151 | 50.9% | OR = 1.07 (1.03–1.12); p = 0.001 | Moderate | 151 prospectively identified consecutive patients with anterior circulation ischemic stroke; followed for 12 months; primary study was regarding EEG findings and association with outcomes |
| Bentes | 2017 | 29588974 | admission | Continuous variable | mortality, discharge | 151 | 4.6% | p = 0.701 | Moderate | 151 prospectively identified consecutive patients with anterior circulation ischemic stroke; followed for 12 months; primary study was regarding EEG findings and association with outcomes |
| Bentes | 2017 | 29588974 | admission | Continuous variable | 1-y mortality | 151 | 15.2% | OR = 1.06 (1.01–1.12); p = 0.028 | Moderate | 151 prospectively identified consecutive patients with anterior circulation ischemic stroke; followed for 12 months; primary study was regarding EEG findings and association with outcomes |
| Liu | 2017 | 29201238 | admission | Continuous variable | 90-d mRS >2 | 116 | 34.5% | OR 1.085 (1.036-1.136); p=0.001 | High | Retrospective study of 116 patients, who all received a follow up MRI and none received thrombolysis for initial stroke treatment |
| Bhaskar | 2017 | 29133696 | admission | Continuous variable | 90-d mortality | 608 | 20.7% | OR 1.01 (0.99-1.03); P=0.303 | Moderate | Retrospective study of 608 patients from 2006-2013; registry-based study; including only patients with complete data |
| Bhaskar | 2017 | 29133696 | admission | Continuous variable | 90-d mRS >2 | 608 | 53.7% | OR 1.03 (1.0111.05); P=0.001 | Moderate | Retrospective study of 608 patients from 2006-2013; registry-based study; including only patients with complete data |
| Xue | 2017 | 28978089 | admission | Continuous variable | 90-d mRS >2 | 438 | 65.7% | OR 1.50 (1.21-1.76); p=0.008 | Moderate | 438 patients prospectively enrolled from 2015-2016 |
| Ahn | 2017 | 28877565 | admission | Continuous variable | 33-m mortality | 1692 | 19.6% | P<0.05 | Moderate | 1692 patients retrospectively included from a single hospital from 2007-2011; all with ECG findings and troponin |
| Kim | 2017 | 28176499 | admission | Continuous variable | 90-d mRS <3 | 104 | 44% | OR 3.67 (1.49–9.06); p=0.01-0.05 | Moderate | 104 patients from a prospective registry of 5558 patients, with endovascular recanalization treatment for LVO |
| Zhang | 2014 | 24994827 | admission | Continuous variable | 90-d mRS >2 | 129 | 57% | OR 1.03 (0.99–1.06); p=0.005 | Moderate | 129 consecutive, prospectively enrolled patients with strok undergoing endovascular therapy at a single hospital from 2006-2013 |
| Shi | 2014 | 24876082 | admission | Continuous variable | 90-d mRS >2 | 228 | 53.5% | OR 1.04 (1.02–1.06); p=0.0005 | Moderate | 2228 patients with successful LVO recanalization from the MERCI, TREVO, and TREVO2 trials |
| Ricciardi | 2014 | 24851928 | admission | Continuous variable | 90-d mRS <3 | 159 | 46.5% | OR 1.01 (0.97-1.05; p=0.591 | Moderate | 159 patients in a prospective registry at a single hospital with LVO, presenting within 12 hours of onset and evaluated using the IPAQ assessing pre-stroke physical activity |
| Wang | 2014 | 24789365 | admission | Continuous variable | mortality, discharge | 326 | 11.7% | P<0.05 | High | 326 patients, first-ever stroke, presenting to a single hospital, with vitamin D levels from 2012-2013 |
| Yoo | 2014 | 24747428 | admission | Continuous variable | 90-d mRS >2 | 207 | 62.8% | <0.001 | Moderate | 207 consecutive patients, retrospective, single stroke center, primarily evaluating glucose and associated stroke outcomes; all patients received IV alteplase within 3 hours |
| Yoo | 2014 | 24747428 | admission | Continuous variable | 90-d mortality | 207 | 15.9% | <0.001 | Moderate | 207 consecutive patients, retrospective, single stroke center, primarily evaluating glucose and associated stroke outcomes; all patients received IV alteplase within 3 hours |
| Cao | 2014 | 24742803 | admission | Continuous variable | 90-d mRS >2 | 55 | 58.2% | Beta 0.097, Standard error 0.043, p=0.024 | High | 217 consecutive stroke patients with alteplase or endovascular therapy, retrospectively evaluated from 2009-2011 |
| Cao | 2014 | 24742803 | admission | Continuous variable | 90-d mortality | 55 | 21.8% | Beta 0.185, Standard error 0.074, p=0.01 | High | 217 consecutive stroke patients with alteplase or endovascular therapy, retrospectively evaluated from 2009-2011 |
| Kim | 2014 | 24719133 | admission | Continuous variable | 30-d mortality | 2820 | 2.8% | P<0.05 | Moderate | 2820 patients, 344 with early neurologic deterioration, which was the primary analysis; 200-2008 single hospital, excluding patients with recurrent stroke, alteplase receipt, and late >3 day presentation |
| Kim | 2014 | 24719133 | admission | Continuous variable | 3-y mortality | 2820 | 17.7% | P<0.05 | Moderate | 2820 patients, 344 with early neurologic deterioration, which was the primary analysis; 200-2008 single hospital, excluding patients with recurrent stroke, alteplase receipt, and late >3 day presentation |
| Inoa | 2014 | 24686370 | admission | Continuous variable | 90-d modified Barthel Index (mBI) <19 | 1569 | 58% | AC: OR 0.97 (0.96–0.98); p<0.001 \| PC: OR 0.96 (0.94–0.99); p=0.004 | Moderate | 1569 patients from a prospective registry, retrospectively analyzed; primary analysis was between anterior and posterior circulation stroke and associated outcomes/prognosis |
| Hao | 2014 | 24685995 | admission | Continuous variable | 90-d mRS >2 | 215 | 36.3% | RR 1.02 (0.99-1.05); p=).241 | Moderate | 215 patients from a prospective registry from 2009-2011 with severe intracranial stenosis or occlusion; included within 30 days of symptom onset; primary evaluation was on high blood pressure upon admission association with outcomes |
| Lee | 2014 | 24534029 | admission | Continuous variable | 90-d mRS <3 | 110 | 83.6% | Non-significant | High | 118 stroke patients with posterior circulation stroke within 6 hours of symptom onset, evaluating DWI lesion characteristics associations on outcomes |
| Gao | 2016 | 27588095 | admission | Continuous variable | Mortality, median 10 months | 619 | 16.50% | OR: 1.06 (1.02-1.10) | Moderate | Prospective study, 619 patients |
| Madsen | 2019 | 30097482 | admission | Continuous variable | 90-d mRS ≤ 2 | 279 | 32.97% | OR: 0.24 (0.05 - 1.09) P=0.06 | Moderate | 279 participants, 2015-2017, prospective study |
| Akhtar | 2019 | 30545720 | admission | Continuous variable | 90-d mRS 3-6 | 2961 | 24.00% | OR: 1.029 (1.018 1.040), p=0.009 | Moderate | Prospective study, 619 patients |
| Gattringer | 2019 | 30580732 | admission | Continuous variable | 7-d mortality | 77653 | 2% | OR: 1.85 (1.36–2.50) | Moderate | 77653 patients, prospective multicenter sutdy |
| Gattringer | 2019 | 30580732 | admission | Continuous variable | 7-d mortality | 77653 | 2% | OR: 2.93 (2.23–3.85) | Moderate | 77653 patients, prospective multicenter sutdy |
| Gattringer | 2019 | 30580732 | admission | Continuous variable | 7-d mortality | 77653 | 2% | OR: 3.04 (2.31–4.0) | Moderate | 77653 patients, prospective multicenter sutdy |
| Gattringer | 2019 | 30580732 | admission | Continuous variable | 7-d mortality | 77653 | 2% | OR: 3.86 (2.85–5.23) | Moderate | 77653 patients, prospective multicenter sutdy |
| di Poggio | 2019 | 30430315 | admission | Continuous variable | 90-d mRS ≤2 | 459 | 40.52% | OR: 5.0 (1.4–64.1) p= 0.01 | High | 461 patients, January 2016 - December 2016, prospective study |
| Shi | 2019 | 30328586 | admission | Continuous variable | 90-d mortality | 391 | 9.50% | P =0.015 | Moderate | retrospective study, 391 participants, 2010-2017 |
| Gory | 2018 | 29393092 | admission | Continuous variable | 90-d mortality | 117 | 41.90% | OR: 5.25 (1.57–17.56) p=0.007 | Moderate | 149 patients, prospective, 2016-2017 |
| Laible | 2017 | 29084408 | admission | Continuous variable | 90-d mRS ≥ 3 | 505 | 66.52% | OR: 1.04 (1.02–1.06)p= 0.001 | Moderate | Prospective, 2010-2016, 505 patients |
| Guo | 2019 | 30530284 | admission | Continuous variable | 90-d mRS(death or major disability) | 3881 | 25.20% | OR: 1.185 (1.028-1.365), p=0.160 | Moderate | There is a possibility of selection bias, although baseline characteristics are similar to the China National Stroke Registry. |
| Li | 2019 | 30396839 | admission | Continuous variable | 90-d mRS 3-6 | 685 | 35.04% | OR: .90 (.53-1.55) p=.709 | Moderate | Retrospective study, 685 participants, 2013-2022 |
| Li | 2019 | 30396839 | admission | Continuous variable | 90-d mRS 3-6 | 685 | 35.04% | OR: 2.11 (1.30-3.44) p=.003 | Moderate | Retrospective study, 685 participants, 2013-2023 |
| Li | 2019 | 30396839 | admission | Continuous variable | AF | 685 | 29.80% | OR: 3.47 (2.06-5.85) p<.001 | Moderate | Retrospective study, 685 participants, 2013-2036 |
| Li | 2019 | 30396839 | admission | Continuous variable | AF | 685 | 29.80% | OR: 6.54 (2.06-10.54) p<.001 | Moderate | Retrospective study, 685 participants, 2013-2037 |
| Boers | 2018 | 29627794 | admission | Continuous variable | 90-d mRS 0-2 | 1665 | 39% | p<0.01 | Moderate | 1665 participants, review of trials |
| Urbanek | 2018 | 30390631 | admission | Continuous variable | 1-y mRS>2 | 1370 | 52.34% | OR: 0.4 (0.2–0.8) p= 0.01 | Moderate | 138 patients, retrospective, 2006-2010 |
| Urbanek | 2018 | 30390631 | admission | Continuous variable | 1-y mRS>2 | 1370 | 52.34% | OR: 1.7 (1.1–2.8) p=0.03 | Moderate | 139 patients, retrospective, 2006-2010 |
| Urbanek | 2018 | 30390631 | admission | Continuous variable | 1-y mRS>2 | 1370 | 52.34% | OR: 3.3 (2.1–5.3) p< 0.01 | Moderate | 140 patients, retrospective, 2006-2010 |
| Urbanek | 2018 | 30390631 | admission | Continuous variable | 1-y mRS>2 | 1370 | 52.34% | OR: 14.5 (7.4–28.5) p< 0.01 | Moderate | 141 patients, retrospective, 2006-2010 |
| Muscari | 2016 | 27186444 | admission | Continuous variable | 9-m mRS | 309 | 51.10% | P<0.0001 | Moderate | 152 patients, retrospective, 2006-2010 |
| Bhatia | 2011 | 21566239 | admission | Continuous variable | 90-d mRS≤ 2 | 251 | 46.61% | OR: 0.137 (0.055–0.341) P<0.0001 | Moderate | NA |
| Kruetzelmann | 2011 | 21415399 | admission | Continuous variable | 90-d mRS 0-1 | 174 | 47.70% | OR: 0.962 (0.932–0.993), p=0.017 | Moderate | prospective, multicenter, 174 patients |
| Kruetzelmann | 2011 | 21415399 | admission | Continuous variable | 90-d mortality | 174 | NR | OR: 1.090 (1.019–1.166) p=0.012 | Moderate | prospective, multicenter, 174 patients |
| Natarajan | 2011 | 21351835 | admission | Continuous variable | 90-d mRS 3-6 | 614 | 61.10% | OR: 3.87 (2.303–5.504) p<0.001 | Moderate | prospective, multicenter, 174 patients |
| Natarajan | 2011 | 21351835 | admission | Continuous variable | 90-d mortality | 614 | 30.80% | OR: 4.507 (2.671–7.605) p<0.001 | Moderate | prospective, multicenter, 174 patients |
| Mustanoja | 2011 | 21106955 | admission | Continuous variable | 90-d mRS 0-2 | 957 | 58% | OR: 1.06 (1.04 - 1.07) | Moderate | Retrospective, 957 participants, 1995-2012 |
| Naganuma | 2011 | 21088392 | admission | Continuous variable | 90-d mRS 0-1 | 578 | NA | OR: 0.97 (0.96–0.99) p=0.005 | Moderate | Retrospective, mulicenter, 578 patients, 2005-2009 |
| Naganuma | 2011 | 21088392 | admission | Continuous variable | 90-d mRS 4-6 | 578 | NA | OR: 1.04 (1.02–1.06) p<0.001 | Moderate | Retrospective, mulicenter, 578 patients, 2005-2013 |
| Putaala | 2011 | 21079397 | admission | Continuous variable | 90-d mRS 3-6 | 851 | 40.50% | OR: 1.06 (1.04–1.07) p<0.05 | Moderate | Retrospective, 1998-2008, 851 patients |
| Putaala | 2011 | 21079397 | admission | Continuous variable | 90-d mortality | 851 | 9.30% | OR: 1.07 (1.04–1.10) p<0.05 | Moderate | Retrospective, 1998-2008, 851 patients |
| Tei | 2011 | 20957383 | admission | Continuous variable | 90-d mRS 3-6 | 350 | 37.70% | OR:1.037 (0.999–1.076) p=0.055 | Moderate | 354 participants, 1994-2009 |
| Alvarez-Perez | 2011 | 20954836 | admission | Continuous variable | mRS >2 at discharge | 200 | 59.50% | OR: 1.052, (1.012–1.093), p=0.010 | High | 204 participants, prospective study |
| Soares | 2011 | 20688404 | admission | Continuous variable | 180-d mRS | 115 | 47.00% | OR 2.389(1.127–5.063), p 0.022 | High | A study conducted from 2003 to 2007 with 115 participants has certain limitations. These include the fact that the study was conducted in a hospital-based setting, there were only a small number of patients with the specific outcome being studied, and composite variables were used instead of examining individual comorbidities. |
| Sanak | 2011 | 20597864 | admission | Continuous variable | 90-d mRS 0-2 | 125 | 66.40% | OR: 0.990 (0.950–1.031), p=0.625 | Moderate | 125 participants, retrospective study, 2004-2008 |
| Ford | 2010 | 20930163 | admission | Continuous variable | 90-d mRS 0-2 | 20348 | 56% | OR: 0.73 (0.68–0.78) p<0.005 | Moderate | 20348 participants, prospective, multicentered, data collected in SITS-ISTR study |
| Ford | 2010 | 20930163 | admission | Continuous variable | 90-d mRS 0-1 | 20348 | 82% | OR: 0.81 (0.75–0.87) p<0.005 | Moderate | 20349 participants, prospective, multicentered, data collected in SITS-ISTR study |
| Ford | 2010 | 20930163 | admission | Continuous variable | 90-d mortality | 20348 | 30% | OR: 1.53 (1.43–1.65)p<0.005 | Moderate | 20350 participants, prospective, multicentered, data collected in SITS-ISTR study |
| Ford | 2010 | 20930163 | admission | Continuous variable | sICH per SITS-MOST | 20348 | 1.80% | OR: 0.90 (0.73–1.09) p=0.28 | Moderate | 20351 participants, prospective, multicentered, data collected in SITS-ISTR study |
| Lima | 2010 | 20829514 | admission | Continuous variable | 180-d mRS ≤ 2 | 196 | 41.84% | OR: 0.95 (0.93 to 0.98) P=0.001 | Moderate | Prospective study, 196 participants |
| Silva | 2010 | 20733301 | admission | Continuous variable | 180-d mRS ≥3 | 676 | 32.50% | OR 1.47 (1.30-1.67), P <0.01 | Moderate | Gold-standard tests like magnetic resonance imaging with diffusion-weighted imaging or follow-up CT scans were not used to confirm the size of ischemic lesions. |
| Tu | 2010 | 20693794 | admission | Continuous variable | 90-d mortality | 101 | 19.80% | OR: 1.02 (0.97–1.07) | High | Prospective study, 101 participants |
| Tu | 2010 | 20693794 | admission | Continuous variable | Parenchymal hematomas | 101 | 33.70% | OR: 1.02 (0.96–1.09) | High | Prospective study, 101 participants |
| Wei | 2010 | 20651267 | admission | Continuous variable | 1-y mRS, Ischemic Stroke subtype | 4782 | 29.97% | OR: 1.05 (1.04–1.05) | Moderate | Prospective, 4782 participants, 2008 |
| Ryu | 2010 | 20627296 | admission | Continuous variable | 1-y mortality | 1067 | 16.50% | HR: 1.05 (1.04–1.07) | Moderate | 1068 patients, prospective study, 2002-2007 |
| Ryu | 2010 | 20627296 | admission | Continuous variable | 1-y mortality, vascular death | 1067 | 10.60% | HR: 1.06 (1.04–1.08) | Moderate | 1074 patients, prospective study, 2002-2007 |
| Arboix | 2010 | 20565890 | admission | Continuous variable | Mortality, in-hospital | 2082 | 11.96% | OR: 1.05 (1.03-1.06) p=0.000 | Moderate | Prospective, 1986-2004, 2082 participants |
| Pan | 2010 | 20510983 | admission | Continuous variable | 180-d BI | 109 | 100% | R- 0.22 (- 0.49 to .047) p= 0.106 | High | 109 participants, prospective study |
| Hao | 2010 | 20332640 | admission | Continuous variable | Reduced eGFR | 1758 | 26.30% | OR: 1.039 (1.028–1.050) p=0.000 | Moderate | Prospective, 1758 participants, 2002-2008 |
| Knauft | 2010 | 20187750 | admission | Continuous variable | 1-y Modified Barthel Index | 122 | NA | B: -0.025, p =0.000 | High | 122 participants, retrospective study, 2001-2008 |
| Sanak | 2010 | 20127250 | admission | Continuous variable | 90-d mRS 3-6 | 157 | 36.94% | OR: 1.035 (0.975–1.099), p=0.256 | High | Retrospective,157 participants, 2004-2008 |
| Chang | 2010 | 20106589 | admission | Continuous variable | 3-y mortality | 356 | 25.60% | HR: 1.08 (1.05–1.11) p<0.001 | High | Prospective study, 360 participants, 1998-2001 |
| Nedeltchev | 2010 | 20104376 | admission | Continuous variable | 30-d mortality | 467 | 13% | HR: 1.12 (1.05–1.19); p<0.001 | Moderate | 467 participants, prospective study, 2003-2007 |
| Caso | 2010 | 20088729 | admission | Continuous variable | 90-d mRS 3-6 | 1136 | 40.05% | OR: 1.07 (1.05–1.09) | Moderate | 2006-2007,1136 participants, prospective study |
| koton | 2010 | 20016218 | admission | Continuous variable | 30-d mortality | 1079 | 9.90% | HR: 1.04 (1.02–1.07) p=0.0002 | Moderate | 1079 participants, prospective |
| Denti | 2010 | 20002511 | admission | Continuous variable | 30-d mortality, older age | 1549 | 19% | HR: 1.47 (1.00–2.16) | Moderate | 1555 participants, 2002-2007, prospective study |
| Denti | 2010 | 20002511 | admission | Continuous variable | 30-d poor outcome, older age | 1549 | 70% | OR: 1.83 (1.37–2.43) | Moderate | 1555 participants, 2002-2007, prospective study |
| Hannon | 2010 | 19893311 | admission | Continuous variable | 90-d mRS | 568 | NA | B: 0.05, p<0.001 | High | 568 participants, prospective study, 2005-2006 |
| Katan | 2009 | 20035506 | admission | Continuous variable | 90-d mRS 3-6 | 362 | 41.70% | OR: 1.06 (1.04–1.09) p<0.0001 | Moderate | Prospective study, 363 participants, 2006-2008 |
| Katan | 2009 | 20035506 | admission | Continuous variable | 90-d mortality | 362 | 12% | OR: 1.07 (1.03–1.12) p=0.002 | Moderate | Prospective study, 363 participants, 2006-2012 |
| Nogueira | 2009 | 19875740 | admission | Continuous variable | 90-d mRS ≤2 | 290 | 32.40% | OR: 0.96 (0.95–0.98) p=0.0004 | Moderate | Prospective study, 290 participants, |
| Saini | 2009 | 19644066 | admission | Continuous variable | 90-d mRS > 2 | 5305 | 58.36% | OR: 2.2 (1.9–2.5) p<0.001 | Moderate | Prospective study, 5305 participants |
| Hallevi | 2009 | 19628935 | admission | Continuous variable | 90-d mRS 0-2 | 1798 | 25.58% | OR: 2.6 (1.8–3.7), p<0.001 | Moderate | Retrospective study, 1798 participants, 1994-2006 |
| Putaala | 2009 | 19590052 | admission | Continuous variable | 5-y mortality | 731 | 10.67% | HR: 1.07 (1.01–1.12) p=0.021 | Moderate | 731 participants, 1994-2003, retrospective study |
| Paciaroni | 2009 | 19419717 | admission | Continuous variable | 90-d mortality | 1467 | 9.20% | OR: 1.0, (1.0–1.1) | High | Prospective study, 1467 patients, 2006-2008 |
| Arsava | 2009 | 19380699 | admission | Continuous variable | 180-d mRS 0-6 | 240 | 100% | OR: 1.29 (1.04–1.60 | Moderate | 240 participants, retrospective study, 2003-2006 |
| Johnston | 2009 | 19228853 | admission | Continuous variable | 90-d mRS 0-1 | 204 | 57% | 0.01 | High | 206 patients, prospective single cenered, |
| Johnston | 2009 | 19228853 | admission | Continuous variable | 90-d mRS 5,6 | 204 | 10.29% | 0.01 | High | 209 patients, prospective single cenered, |
| Johnston | 2009 | 19228853 | admission | Continuous variable | 90-d mRS 0-1 | 204 | 57% | 0.01 | High | 206 participants, observational |
| Kissela | 2009 | 19109548 | admission | Continuous variable | 90-d mRS | 451 | NA | B: 0.01 (0.00–0.02) 0.008 | High | Retrospective, 451 patients, 1991 |
| Fuentes | 2009 | 19095970 | admission | Continuous variable | 90-d mRS >2 | 476 | 63.30% | OR: 1.060 (1.023–1.098), p=0.001 | Moderate | Prospective study, multicenter, 476 participants, 2002-2004 |
| Khartionova | 2009 | 19018138 | admission | Continuous variable | Hyperdense middle cerebral artery sign(HMCA) | 10023 | 19% | B: -0.012, p<0.001 | High | 1023 participants, prospective, 2002-2006 |
| Reggiani | 2009 | 19018135 | admission | Continuous variable | 180-d survival | 361 | 84% | HR: 4.7, p<0.05 | Moderate | prospective study, 361 participants, 1999 |
| Reggiani | 2009 | 19018135 | admission | Continuous variable | 5-y survival | 361 | 64% | HR: 5.4, p<0.0001 | Moderate | prospective study, 361 participants, 2002 |
| Stead | 2009 | 18357419 | admission | Continuous variable | 90-d mortality | 447 | 17.67% | P<0.05 | High | 447 participants, 2001-2004 |
| Tartaglia | 2008 | 19235441 | admission | Continuous variable | 90-d mRS 3-6 | 130 | 58.50% | P<0.05 | High | 131 participants, retrospective study, 1998-2005 |
| Millan | 2008 | 19049558 | admission | Continuous variable | 90-d mRS 3-6 | 254 | 44.10% | OR: 1.02 (0.99 to 1.06)p= 0.124 | Moderate | 254 participants, retrospective study |
| Hong | 2008 | 19049549 | admission | Continuous variable | 90-d mRS 3-6 | 1254 | 34.90% | OR: 2.32 (1.58–3.42) | Moderate | 1261 participants, prospective study, 2004-2005 |
| Wahlgren | 2008 | 18927461 | admission | Continuous variable | 90-d mRS 0-2 | 6947 | 50% | OR: 0.74 (0.69-0.79) | Moderate | 6947 participants, review of RCT, 2002-2006 |
| Arboix | 2008 | 18817678 | admission | Continuous variable | Mortality, in-hospital | 2704 | 12.90% | OR: 1.05 (1.03-1.06) p<.001 | Moderate | 1267 participants, prospective study, 2004-2005 |
| Arboix | 2008 | 18817678 | admission | Continuous variable | Mortality, in-hospital | 2704 | 12.90% | OR: 1.06 (1.04-1.08) p<.001 | Moderate | 1269 participants, prospective study, 2004-2005 |
| Arboix | 2008 | 18817678 | admission | Continuous variable | Mortality, in-hospital | 2704 | 12.90% | OR: 1.05 (1.01-1.09) p=.005 | Moderate | 1271 participants, prospective study, 2004-2005 |
| Puetz | 2008 | 18811738 | admission | Continuous variable | 90-d mRS 0-2 | 263 | 45.20% | OR: 0.96 (0.94–0.98) p<0.001 | Moderate | Retrospective study, 263 participants, 2002-2010 |
| Puetz | 2008 | 18811738 | admission | Continuous variable | 90-d mortality | 263 | 14.10% | OR: 1.04 (1.01–1.08) p=0.012 | Moderate | Retrospective study, 263 participants, 2002-2012 |
| Yong | 2008 | 18703813 | admission | Continuous variable | 90-d mortality | 748 | 9.80% | P < 0.01 | Moderate | 753 participants, patient sample from randomized controlled trial |
| Arnold | 2008 | 18677634 | admission | Continuous variable | 90-d mRS 6 | 1004 | 10.86% | p=0.051 | Moderate | prospective study, 1004 participants, 2000-2006 |
| Ovbiagele | 2008 | 18583560 | admission | Continuous variable | 90-d mRS ≥ 2 | 659 | 16.55% | P<0.01 | High | 659 participants, prospective study of RCT |
| Saposnik | 2008 | 1856630 | admission | Continuous variable | 30-d mortality | 3631 | 12.60% | OR: 1.04 (1.03–1.06) | Moderate | Retrospective, 3361 participants, 2003-2009 |
| Saposnik | 2008 | 1856630 | admission | Continuous variable | 1-y mortality | 3631 | 23.60% | OR: 1.05 (1.04–1.06) | Moderate | Retrospective, 3361 participants, 2003-2016 |
| Uyttenboogaart | 2008 | 18338194 | admission | Continuous variable | 90-d mRS≤ 2 | 252 | 49% | OR: 0.93 (0.90–0.96) p< 0.001 | Moderate | 254 participants, prospective study, 2002-2006 |
| Roquer | 2007 | 18004645 | admission | Continuous variable | Mortality, in-hospital | 1527 | 12.90% | HR: 1.033 (1.016–1.051), p=0.001 | Moderate | 1997-2005, 1527 participants, prospective study |
| Liu | 2007 | 17600616 | admission | Continuous variable | 2-y mRS 3-6 | 489 | 43.80% | OR: 3.505 (2.100 - 5.849), p=0.000 | High | Retrospective, 489 patients, 2002-2005 |
| Ois | 2007 | 17525388 | admission | Continuous variable | 90-d mortality, (with NIHSS ≤7) | 1220 | 15.70% | OR: 1.05 (1.02–1.09), p<0.05 | Moderate | 1222 participants, prospective study, 2003 to 2006 |
| Ois |  | 17525388 | admission | Continuous variable | 90-d mortality, (with NIHSS >7) | 1220 | 15.70% | OR: 1.05 (1.03–1.08), p<0.05 | Moderate | 1223 participants, prospective study, 2003 to 2006 |
| Roquer | 2007 | 17502469 | admission | Continuous variable | 30-d mortality | 1527 | 13.80% | OR: 1.05 (1.03-1.08)p<.001 | Moderate | 1532 participants, 1997-2005, retrospective study |
| Nybo | 2007 | 17437604 | admission | Continuous variable | 180-d mortality | 250 | 9.20% | OR: 3.3 (1.0–11.0) | Moderate | Risk of death or dependence at 3 months is 6.77% in young patients with moderate deficits and no prior stroke, compared to 75% in older patients with previous stroke and severe deficits. Validation in different populations is needed. |
| Elkind | 2007 | 17353472 | admission | Continuous variable | BI ≥ 95 | 333 | 39.60% | OR: 0.96 (0.94–0.99) | Moderate | 338 participants, review of randomized controlled trial |
| Elkind | 2007 | 17353472 | admission | Continuous variable | 90-d mortality (Alive) | 333 | 80.91% | OR: 0.97 (0.94–1.00) | Moderate | 340 participants, review of randomized controlled trial |
| Montaner | 2006 | 1660121 | admission | Continuous variable | 90-d mortality | 143 | 13.29% | OR: 4.45 (1.14 to 17.37) P=0.032 | High | 144 participants, prospective study |
| Wu | 2017 | 28579505 | admission | Continuous variable | 90-d mRS dichotomized 0-2 vs 3-6 | 383 | 66% | AOR 1.022, 95% CI 1.005-1.174 (p=0.038) | High | Thrombolysis only included; no loss to follow-up description; unknown missing data and no imputation; presumed high risk of bias due to no mention of WLST. |
| Shi | 2017 | 28356003 | admission | Continuous variable | 90-d mRS | 373 | 77% | Young:  AOR 2.09, 95% CI 1.09-3.99; p=0.026 | High | Thrombolysis only included; mRS obtained by care provider without standardization, increasing outcome assessment bias; no definition of favorable vs unfavorable, unclear handling of missing data. |
| Shi | 2017 | 28356003 | admission | Continuous variable | 90-d mortality | 373 | NA | AOR 0.57, 95% CI 0.16-20.8; p=0.094 | High | Thrombolysis only included; mRS obtained by care provider without standardization, increasing outcome assessment bias; no definition of favorable vs unfavorable, unclear handling of missing data. |
| Munsch | 2016 | 26585396 | admission | Continuous variable | 90-d mRS score ≤1 | 428 | 43.2% | Not significant in linear regression | Moderate | Highly selected patients: only those with MRI wihtin 24-72h of stroke and assessed for functional and cognitive outcomes 3 months later; great statisticcs but no imputation of missing data; no word on WLST |
| Munsch | 2016 | 26585396 | admission | Continuous variable | 90-d MoCA | 428 | 46.4% with a good cognitive outcome (MoCA>25) | Significant in linear regression | Moderate | Highly selected patients: only those with MRI wihtin 24-72h of stroke and assessed for functional and cognitive outcomes 3 months later; great statisticcs but no imputation of missing data; no word on WLST |
| Shi | 2016 | 26409718 | admission | Continuous variable | 1-y death/dependency = mRS 3-6 | 2168 | NA | AOR 1.04 (955 CI 1.03-1.05) | Moderate | Single Chinese hospital registry, included patients with AIS within 14 days of symptom onset who had a complete 12-month follow-up; no data on loss to follow up or imputation; adequate analysis, no mention at all about WLST and how patients died |
| Dogan | 2016 | 25228671 | admission | Continuous variable | in-hospital mortality | 692 | 247 (35.7%) | AOR 1.038 95% CI [0.982-1.097] | High | Restricted to patients >=65; excluded all who had incomplete data (no imputation), no comparison of those with or without complete data, unclear how many had missing data, retrospective only, no data on WLST |
| van Seeters | 2015 | 26358136 | admission | Continuous variable | 90-d poor mRS (3-6) | 1374 | 36% | AOR 1.45 (95% CI 1.26-1.67) | Moderate | Data from multicenter prospective study at 14 centers in Netherlands; split cohort for derivation/validation 60/40%; excellent analysis with discrimination AND 2 different calibration measures reported; 6% incomplete data and 1% missing 90d mRS excluded; no imputation of missing data; no word on WLST |
| Haeusler | 2015 | 26349854 | admission | Continuous variable | 8-y Long-term mortality | 1013 | 42% | AOR 1.7 (95% CI 1.53-1.87), p<0.001 | High | Retrospective study at 3 Berlin hospitals, ICD-10 dx, mRS outcome, 8-year survival from German civil registration; median follow-up 80 months; excluded pts without survival status; no comparisons between missing data groups; no mention of mode of death. |
| Ntaios | 2017 | 28687720 | admission | Continuous variable | Ischemic stroke/TIA recurrence | 1,095 | 16.7% | Age 60-80:  HR 1.90, 95% CI 1.21–2.98, p = 0.005  Over 80:  HR 2.71, 95% CI 1.57–4.70, p < 0.001 | Low | Global study, 1000+ patients (11 centers), ~3-year follow-up; high attrition, moderate confounding; acceptable with some loss to follow-up; main limitation: unaddressed confounding factors. |
| Ntaios | 2017 | 28687720 | admission | Continuous variable | mortality | 1,095 | 15.2% | Age 60-80:  HR 4.43, 95% CI 2.32–8.44, p < 0.001  Over 80:  HR 8.01, 95% CI 3.98–16.10, p < 0.001 | Low | Global study, 1000+ patients (11 centers), ~3-year follow-up; high attrition, moderate confounding; acceptable with some loss to follow-up; main limitation: unaddressed confounding factors. |
| Kammersgaard | 2005 | 21852614 | admission | Continuous variable | 5-y mortality | 899 | 58.4% | Increasing age HR 1.7, 95% CI 1.5-1.8 | Low | Study attrition- only 6 patients lost due to moving out of the country; outcome measurement- all causes of death attainable through Danish Central Registry; |
| Nedeltchev | 2004 | 15654030 | admission | Continuous variable | 90-d mRS favorable = 0-1 unfavorable = 2-6 | 203 | NA | Not significant | High | Study confounding- moderate; statistical analysis and reporting- high; self-fulfilling prophecy- moderate |
| Cucchiara | 2004 | 14707586 | admission | Continuous variable | 30-d mortality | 564 | NA | OR 2.86, 95% CI 1.58-5.19, p<0.001 | Low | Study attrition- moderate; study confounding- moderate |
| Cucchiara | 2004 | 14707586 | admission | Continuous variable | 1-y mortality | 998 | NA | OR 6.64, 95% CI 3.84-11.50, p<0.001 |  | Outcome measurement- high |
| Sumer MM | 2003 | 12675701 | admission | Continuous variable | 180-d independent (mRS 1-2) dependent (mRS 3 -6) | 266 | NA | p<0.001 | Moderate | Study attrition- moderate; outcome measurement- moderate |
| Demchuk | 2001 | 11502916 | admission | Continuous variable | good outcome = mRS <2 and poor outcome mRS ≥2 | 616 | 33% | OR 0.99 95% CI 0.978-0.996, p=0.006 | High | Large proportion of eligible patients were excluded from study. NIHSS and mRS evaluation didn't seem super valid/reliable. |
| Szczudlik | 2000 | 11208287 | admission | Continuous variable | 90-d mortality Oxfordshire Scale | 329 | NA | p≤0.05 | Low | Well-designed study |
| Arboix | 2000 | 10642019 | admission | Continuous variable | In-hospital mortalitY | NA | NA | p<0.001 | Low | Study confounding- low; statistical analysis and reporting- low; self-fulfilling prophecy- moderate |
| Arboix | 2000 | 10642019 | admission | Continuous variable | mortality at discharge | 262 | NA | p<0.001 | Low | Study confounding- low; statistical analysis and reporting- low; self-fulfilling prophecy- moderate |
| Jorgenson | 2020 | 10512899 | admission | Continuous variable | good functional outcome = BI ≥ 50 points | 233 |  | Decreasing age  OR 0.50, 95% CI 0.25-0.99, p=0.04 | Low | Study bias exists due to the retrospective design and post-hoc identification of factors associated with good outcomes in severe strokes. |
| Baptista | 1998 | 10475103 | admission | Continuous variable | Vital status at hospital discharge (alive/dead) | 3362 | Under 55 years, 7.8% 55-64 years, 18.4% 65-74 years. 16.1% >75 years, 15.9% | Age only significant if stroke subtype was LAD  OR 1.61 CI 1.04-2.5, p=0.049 | Low | Registry based study, other factors, influenced by age, played a role in predicting outcome. |
| Adams | 1999 | 10408548 | admission | Continuous variable | 7-d excellent outcome Glasgow Outcome Scale (GOS) =1 BI =19-20 | 1268 | NA | OR 0.96, 95% CI 0.95-0.97 | Low | Self-fulfilling prophecy- moderate |
| Adams | 1999 | 10408548 | admission | Continuous variable | 90-d excellent outcome Glasgow Outcome Scale (GOS) =1 BI =19-20 | 1268 | NA | OR 0.98, 95% CI 0.96-0.99 | Low | Self-fulfilling prophecy- moderate |
| Pohjasvaara | 1998 | 9445332 | admission | Continuous variable | 90-d post stroke dementia after ischemic stroke (as according to DSM-III) | 337 | 31.8% | P<0.001 | Low | Well conducted study |
| Toni | 1997 | 8996480 | admission | Continuous variable | Improving, stable, deteriorating as defined by CNS scale | 152 | NA | p=0.003 | Low | Self-fulfilling prophecy- moderate |
| Finocchi C. | 1996 | 8933227 | admission | Continuous variable | Mortality and disability as defined by Oxford Disability scale (ODS) evaluated on day 30  deceased ODS 3-5 = disabled ODS 0-2 = not disabled  good = 190/351 (54%) poor = 1118 (34%) | 351 | NA | Significant | Low | Self-fulfilling prophecy- moderate |
| Censori | 1996 | 8685929 | admission | Continuous variable | 90-d dementia after first ischemic stroke (defined by NINS-AIREN criteria) | 146 | NA | OR 45.8, 95% CI 2.9-726.0 p<0.003 | Moderate | Study attrition- moderate; study confounding- moderate; self-fulfilling prophecy- moderate |
| Henon | 1995 | 7886712 | admission | Continuous variable | 8-d mortality rate  90-d functional outcome (Glasgow Outcome Scale)  Independent = GOS score 1-2 dependent or dead = GOS score 3-5 | 152 | NA | P=0.0008 | Low | Risk of death or dependence at 3 months is 6.77% in young patients with moderate deficits and no prior stroke, compared to 75% in older patients with previous stroke and severe deficits. Validation in different populations is needed. |
| Sacco | 1994 | 8164815 | admission | Continuous variable | 30-d mortality   1-y mortality  5-y mortality | 323 | NA | p<0.01 | Low | Well conducted study |
| Tatemichi | 1993 | 8498836 | admission | Continuous variable | Prevalence of dementia at 3mo visit post stroke  defined by DSM-III-R criteria. | 251 | NA | Significant | Low | Lesion characteristics alone may not fully cause dementia. Age, education, and race also influence susceptibility to cognitive decline. |
| Skafida | 2018 | 31008348 | admission | Continuous variable | 90-d mortality | 1271 | 18.9% | p=0.001 | Low | Single-center study, lack of standardized therapy, inability to control for drug use, and limitations in causality determination. |
| Timsit | 2016 | 31008289 | admission | Continuous variable | 28-d late mortality | 3024 | 38.49% | HR 1.84, 1.66-2.02; p<0.0001 | Low | Study participation- early mortality occurred in 313 (9.3%) of patients during the first 28d |
| Zhang | 2017 | 24994827 | admission | Continuous variable | vital status at discharge (alive v dead) | 880 | NA | HR 1.03 (1.24-197), p=0.067 | Low | 129 consecutive, prospectively enrolled patients with stroke undergoing endovascular therapy at a single hospital from 2006-2013 |
| Toon | 2017 | 28178407 | admission | Continuous variable | 90-d good functional outcome = mRS 0-2 | 335 | 45.1% | AOR 0.965, 95% CI 0.944-0.986  p=0.001 | Low | Study attrition: 2 participants were lost to follow-up. Outcome measurement: The Modified Rankin Scale (mRS) was assessed by a stroke neurologist during the 3-month outpatient visit. If an in-person assessment was not possible, it was conducted through a telephone interview. |
| Pikija | 2018 | 30353493 | admission | Continuous variable | good outcome = mRS 0-2 |  | 50% | AOR 0.944, 95% CI 0.90-0.99 p=0.012 | Low | The study had limitations, including its retrospective design, LDL-C was major predictor evaluated. The demographics, stroke severity, and outcomes were comparable to large EVT trials, with similar rates of favorable outcomes and mortality. |
| Potreck | 2019 | 30887195 | admission | Continuous variable | 90-d good outcome = mRS ≤2 | 131 | n=49 | OR 0.96, 0.92-1.00, p=0.036 | Low | Study attrition- retrospective study, n=156. 25 excluded due to image quality. These patients could have had more severe/complicated cases or just due to other reasons. ; outcome measurement- image quality was evaluated blindly, excluding 25 patients |
| Wang | 2020 | 32484415 | admission | Continuous variable | 90-d good outcome = mRS 0-2 | 119 | 47.9% | P<0.001 | Low | Well conducted study |
| Henden | 2018 | 30117146 | admission | Continuous variable | 90-d good outcome = mRS 0-2 | 198 | 39% | OR 1.045, 95% CI 1.011-1.080, p=0.010 | Low | This retrospective study had limitations in terms of hospital admission times and classification of on-hour vs off-hour management. However, it captured extensive data on stroke characteristics and peri-procedural variables. Notably, off-hour management did not independently predict poor outcomes. |
| Madelung | 2017 | 28905995 | admission | Continuous variable | 90-d good outcome = mRS ≤2 | 187 | 47% | OR 0.97, 0.95-1.00, p=0.051 | Moderate | Did not say protocol for how mRS outcome was evaluated. |
| Broocks | 2019 | 31667625 | admission | Continuous variable | 90-d mRS 0-2 3-6   n=69 (39%) mRS 0-2 at 90days  n=109 (61%) mRS 3-6) at 90days | 178 | NA | AOR 0.95, 0.91-0.99 p<0.01 | High | The study had missing information and protocol details, but other aspects were well-documented. |
| Oliveira | 2019 | 31351827 | admission | Continuous variable | in hospital mortality in very elderly patients (age 80 or older)  overall mortality 26.1% | 195 | NA | OR 1.097, 95% CI 1.00-1.20 p=0.047 | Moderate | Possible selection bias in the study group and a significant impact of infection on mortality (43.1% infection rate) were observed. |
| Wollenweber | 2019 | 31337298 | admission | Continuous variable | 90-d good outcome = mRS score 0-2 | 2637 | 37% | Younger age  OR 1.06; 1.05-1.07 |  | Study participation- moderate; study attrition- moderate ROB |
| Kufner | 2013 | 23287785 | admission | Continuous variable | 90-d mRS (favorable equal or less than 2, unfavorable >2) | 148 | 44% | P<0.001 | Low | Study participation- moderate ROM |
| Forti | 2013 | 23243075 | admission | Continuous variable | mortality in hospital | 769 | 11.7% | OR 1.02 95% CI (0.93-1.12), p = 0.641 | Low | No post-hospital discharge information, small sizes. In-hospital therapy confounders not evaluated. Moderate accuracy of multivariate models suggests important predictors may be missing. Study population may not fully represent age-related stroke unit admissions due to thrombolytic treatment exclusion. |
| Biteker | 2012 | 22527238 | admission | Continuous variable | mortality | 408 | NA | Significant | Low | The study was conducted at a single center and excluded patients with prior stroke and those under 50 years old, limiting generalizability. |
| Yoo | 2020 | 22426317 | admission | Continuous variable | 90-d good functional outcome (mRS 0-2) | 107 | NA | OR 0.934, 95% CI 0.897-0.972, p=0.0007 |  | Outcome measurement- moderate |
| Redfors | 2012 | 22339042 | admission | Continuous variable | Recurrent vascular event (stroke, TIA, coronary event) or death | 594 | NA | HR (95% CI) 1.03 (1.01-1.06); p<0.05 | Low | The study relies on hospitalized cases, potentially excluding milder strokes. Retrospective ascertainment of vascular events through telephone interviews may underestimate event rates, despite using multiple sources for comprehensive data collection. The low mortality and event rates limit the power of multivariable analyses, potentially missing some associations. |
| Redfors | 2012 | 22339042 | admission | Continuous variable | poor functional outcome (mRS 3-6) | 594 | NA | HR (95% CI), 1.04 (1.00-1.07) ; p<0.05 | Low | The study relies on hospitalized cases, potentially excluding milder strokes. Retrospective ascertainment of vascular events through telephone interviews may underestimate event rates, despite using multiple sources for comprehensive data collection. The low mortality and event rates limit the power of multivariable analyses, potentially missing some associations. |
| Scheitz | 2012 | 22326514 | admission | Continuous variable | unfavorable outcome (mRS >2 at discharge) | 715 | NA | p<0.001 | Low | The retrospective design limits result interpretation |
| Knoflach | 2012 | 22238419 | admission | Continuous variable | 90-d mRS score (less than or equal to 2) | 14256 | 92.1% | OR 0.18 (0.14 - 0.22) For age > 85 compared to 56-65 | Low | The registry is stroke unit-based, not population-based, potentially excluding certain patient groups. Data availability is limited due to anonymity protection and lack of reassessment. Follow-up data was available in a subgroup only. |
| Rost | 2012 | 22116811 | admission | Continuous variable | 180-d good outcome (mRS 0-2) | 569 | NA | OR 0.97, 95% CI, 0.4-0.99 | Low | Retrospective analysis hampers assessment of residual confounding and timing of stroke symptom onset interaction. |
| Kuwashiro | 2011 | 21992812 | admission | Continuous variable | 90-d poor functional outcome (defined as NIHSS score greater or equal to 3) | 241 | NA | AOR 1.07, 95% (CI) 1.01 to 1.13, p=0.017, per 1-year increase | Low | This study only included patients with first time ischemic stroke and who also had DM |
| Boers | 2018 | 29627794 | admission | Continuous variable | 90-d independence = mRS 0-2 | 651 | 39.50% | AOR = 0.62 per 10 years (95% CI 0.57-0.67; P < .001) for every mRS 1-point increase | Low | Lack of follow-up NIHSS scores in most trials, limiting assessment of its predictive value. Simplified infarct location analysis may underestimate the relation between infarct volume and outcome. Variations in MRI usage and inclusion criteria across trials may introduce bias. Confounding effects from specific trials and variations in reperfusion rates should be considered. Moderate influence of self-fulfilling prophecy. |
| Sennfalt | 2018 | 30580719 | admission | Continuous variable | 5-y mortality | 20065 | 50.60% | HR (95% CI) 1.06 (1.06–1.07) | Low | Incomplete data on survival and functional outcome for both cohorts at various time points. Reliance on patient-reported information for mRS scores reduces reliability. Inconsistency in criteria for functional dependency before stroke and during follow-up. Use of level of consciousness as a proxy for stroke severity due to unavailable data. Selection bias and reliance on multiple imputation introduce uncertainty in estimates. |
| Ong | 2016 | 28934363 | admission | Continuous variable | In-hopsital mortality | NA | 3.10% | p=0.31 for age dichotomized at 75 years | Low | One-hospital based study with potential limited generalizability to urban areas. Missing information on pre-stroke conditions and timing of stroke onset to hospital admission. Approximately 25% loss to follow-up at one year, but no gender-based differences in follow-up rate. |
| Corso | 2014 | 27437502 | admission | Continuous variable | Long-term mortality (mean 3.3; over up to 8 years) | NA | 51.50% | HR 2.02 (1.65–2.47), p <0.0001 for dichotomized age at 85+ vs. 84- | Low | The predominantly Caucasian population may limit generalizability to other ethnic groups. Use of prestroke mRS score is not standardized. The study did not investigate the impact of hospital treatment practices. |
| Rangaraju | 2016 | 27586683 | admission | Continuous variable | 30-d poor outcome = mRS 4-6 |  | 65.40% | HR 1.04 (0.99-1.04), p = 0.14 | Low | Lack of information on early withdrawal of care and its potential influence on outcome assessment. Use of a 1-month instead of a 3-month follow-up for the primary outcome measure (mRS) may underestimate long-term recovery. Exclusion of patients without a 24-48 hour NIHSS and limited data on post-discharge variables and outcomes beyond 1 month. Lack of inclusion of patients over the age of 85 and no assessment of the relationship between 24-48 hour NIHSS and long-term quality of life. |
| Rangaraju | 2016 | 27586683 | admission | Continuous variable | 90-d good outcome (mRS 0-2) |  | 54.00% | OR, 0.97 (0.93–0.97) P<0.01 | Low | Lack of information on early withdrawal of care and its potential influence on outcome assessment. Use of a 1-month instead of a 3-month follow-up for the primary outcome measure (mRS) may underestimate long-term recovery. Exclusion of patients without a 24-48 hour NIHSS and limited data on post-discharge variables and outcomes beyond 1 month. Lack of inclusion of patients over the age of 85 and no assessment of the relationship between 24-48 hour NIHSS and long-term quality of life. |
| Kim | 2016 | 30355207 | admission | Continuous variable | 90-d mRS 3-6 (="poor outcome") |  |  | OR 1.03 (1.02-1.05) | Low | 308 patients from three interventional studies using the Solitaire stent retriever; LVO only, undergoing endovascular therapy; prospectively collected data, retrospectively analyzed |
| Chang | 2016 | 26835227 | admission | Continuous variable | 180-d functional independence (per FIM) |  | 38% | OR 0.945 ().935 - 0.956), p <0.001 | Moderate | Study participation- moderate; study attrition- moderate; prognostic factor measurement- moderate; statistical analysis and reporting- moderate |
| Chang | 2016 | 26835227 | admission | Continuous variable | Satisfaction' (per Euro Quality of life 5D) |  | 52% | 0.966 (0.955-0.977), p<0.001 | Moderate | Study participation- moderate; study attrition- moderate; prognostic factor measurement- moderate; statistical analysis and reporting- moderate |
| Phan | 2016 | 26796056 | admission | Continuous variable | poor outcome = mRS 2-6 | 98 | 46% | OR 1.07 per year (1.0-1.1, p <0.001) | Low | Study participation- moderate; study attrition- moderate; statistical analysis and reporting- moderate |
| Phan | 2016 | 26796056 | admission | Continuous variable | 90-d mortality | 16 | 7.60% | nonsignificant | Low | Study participation- moderate; study attrition- moderate; statistical analysis and reporting- moderate |
| Tziomalos | 2016 | 25512800 | admission | Continuous variable | mRS 2-5 at discharge | 322 | 58.30% | RR 1.17 (1.09-1.25, p <0.001) | Low | Lack of available magnetic resonance imaging and arterial imaging, hindering determination of stroke subtypes and assessment of stroke location's impact on functional outcome. |
| Tziomalos | 2016 | 25512800 | admission | Continuous variable | mortality at discharge | 56 | 9.20% | nonsignificant | Low | Lack of available magnetic resonance imaging and arterial imaging, hindering determination of stroke subtypes and assessment of stroke location's impact on functional outcome. |
| de Rueda | 2015 | 26219650 | admission | Continuous variable | mortality at discharge | 85 | 57% | OR 1.04 (1.01-1.07) p = 0.019 | Low | Moderate study attrition and self-fulfilling prophecy. Small sample size, residual confounding, reader bias, limited collateral assessment, imperfect correlation with MRI concepts, and absence of clot measurements. |
| Skagen | 2015 | 25922156 | admission | Continuous variable | 90-d good clinical outcome = mRS <2 | 28 | 60.9%% | OR 1.0 (.97 - 1.1), p =0.287 | Low | Study attrition- moderate; prognostic factor measurement- moderate; outcome measurement- moderate; self fulfilling prophecy- moderate |
| Skagen | 2015 | 25922156 | admission | Continuous variable | 90-d good clinical outcome = mRS <2 | 40 | 37.70% | OR 1.0 (0.97-1.1), p =0.56 | Low | Study attrition- moderate; prognostic factor measurement- moderate; outcome measurement- moderate; self fulfilling prophecy- moderate |
| Lee | 2017 | 29067622 | admission | Continuous variable | 180-d mortality | NA | NA | nonsignificant | High | Prognostic factor measurement- high; outcome measurement- high, study confounding- moderate; statistical analysis and reporting- high, self fulfilling prophecy- moderate |
| Lee | 2017 | 29067622 | admission | Continuous variable | 180-d good functional outcome = mRS 2 or less | NA | NA | nonsignificant | High | Prognostic factor measurement- high; outcome measurement- high, study confounding- moderate; statistical analysis and reporting- high, self fulfilling prophecy- moderate |
| Rusanen | 2015 | 26352696 | admission | Continuous variable | 90-d good outcome = mRS 2 or less | NA | NA | OR 0.95 per year (0.90–1.01), p = 0.09 | High | Study attrition-moderate rob, no info on loss to f/u; prognostic factor measurement- unknown prevalence of outcome; outcome measurement- moderate ROB, study confounding- moderate ROB; statistical analysis and reporting- moderate ROB, unknown prevalence of outcome; self-fulfilling prophecy- moderate, unknown prevalence of mRS including death, no mention of WDLST |
| Chen | 2018 | 30166435 | admission | Continuous variable | 90-d Lawton ADL scale | NA | NA | Beta 0·08 (0·01–0·14), p = 0.01 | High | Retrospective design with potential selection bias. Variation within a thrombus may affect representativeness of mean attenuation measurements. Influence of overlapping vessels, calcified clots, and vascular calcification on attenuation measurements. Need for further research on DSA blood flow status and confirmation in larger, multicenter cohorts. |
| Chen | 2018 | 30166435 | admission | Continuous variable | 90-d SSQOL | NA | NA | Beta -1.75 (-0.43 -0.08), p <0.001 | High | Retrospective design with potential selection bias. Variation within a thrombus may affect representativeness of mean attenuation measurements. Influence of overlapping vessels, calcified clots, and vascular calcification on attenuation measurements. Need for further research on DSA blood flow status and confirmation in larger, multicenter cohorts. |
| Sillanppa | 2015 | 22566978 | admission | Continuous variable | 90-d good outcome = mRS 0-2 | 54 | 52% | OR 0.94 (0.89 - 0.98) p = 0.007 | Moderate | Study attrition- only one loss to f/u; prognostic factor measurement- moderate ROB; statistical analysis and reporting- moderate ROB; self-fulfilling prophecy- moderate ROB |
| Lima | 2014 | NA | admission | Continuous variable | 180-d good outcome = mRS -2 or more | 56 | 44.40% | OR 0.94 (0.91-0.98) , p =0.001 | Low | Outcome measurement- moderate ROB |
| Perez | 2014 | 24251821 | admission | Continuous variable | poor outcome (mRS 3-6) |  | 75% | OR 1.07 (1.01-1.13) | Low | Detection of occlusion using TCDx is less sensitive and precise compared to other angiographic methods. Some loss to follow-up, particularly in the proximal MCA occlusion group. Some patients may not meet current eligibility criteria for reperfusion therapies. However, similar results were obtained when analyzing the subgroup of patients who potentially met current treatment criteria. |
| Tong | 2014 | NA | admission | Continuous variable | in-hospital mortality | 516 | 7.20% | OR 2·92, 95% CI 1·99–4·29, P < 0·001 | Low | Overall low for age and NIHSS as predictors. Did not review papers main predictors |
| Tong | 2014 | NA | admission | Continuous variable | poor outcome mRS 3-6 | 199 | 51%% | OR 1·039; 95% CI 1·017–1·061), P = 0·001 | Low | Self-fulfilling prophecy- moderate |
| Yeo | 2013 | 23599933 | admission | Continuous variable | 90-d favorable outcome = mRS 0-1 | 130 | 49.40% | Nonsignificant | Low | Limited arterial recanalization data, taking wither within 2 or at 24 hours, excluding potential recanalization in between. Recanalization-reocclusion-recanalization phenomenon may impact outcomes. Missing data. |

**BLOOD GLUCOSE**

| First author last name | Year | PMID | Timing of prognostic factor evaluation | Prevalence % of the predictor | Outcome(s) | Sample size for the outcome | Prevalence % of the outcome | Effect size | Overall risk of bias | Overall risk of bias: comments |
| --- | --- | --- | --- | --- | --- | --- | --- | --- | --- | --- |
| Wu | 2017 | 28579505 | admission | Continuous | 90 day mRS dichotomized 0-2 vs. 3-6 | 383 | Favorable outcome: 253 (66%), unfavorable 130 (34%) | AOR 1.13, 95% CI 1.016-1.256 (p=0.024) | Moderate | Thrombolysis only included; no loss to follow-up description; unknown missing data and no imputation; presumed high risk of bias due to no mention of WLST. |
| Shi | 2016 | 26409718 | admission | 15.1% | 1-y mortality | 2168 | 839 (38.7%) | AOR 1.50 (95% CI 1.10-2.04) | Moderate | Single Chinese hospital registry; included AIS patients within 14 days of symptom onset with complete 12-month follow-up; no information on loss to follow-up or imputation; analysis deemed sufficient; no mention of WLST or details on patient mortality s. |
| Dogan | 2016 | 25228671 | admission | Continuous | In-hospital mortality | 692 | 247 (35.7%) | AOR 2.610 95% CI [1.023-6.660] | High | Restricted to patients aged 65 and above; excluded those with incomplete data (no imputation); no comparison between groups with complete and incomplete data; unclear number of patients with missing data; retrospective study design only; no information on WLST. |
| Zhu | 2017 | 28716236 | Within 24 hrs of admission | total of 3882 patients 53.1% normal FPG 10.6% IFG 36.3% DM | Unfavorable outcome = mRS 3-6 | 3882 | 25.14% | IFG OR 1.25 (95% CI 0.92 - 1.68)  DM OR 1.38 (1.13-1.67) | Moderate | Blood samples after 8 hrs of fasting, taken within 24 hrs of admission |
| Cucchiara | 2004 | 14707586 | admission | Continuous | mortality | 564 | NA | p=0.01 | Low | Well-designed study |
| Cucchiara | 2004 | 14707586 | Within 24 hrs of admission | Continuous | mortality | 564 | NA | bivariate analysis p<0.001 | Low | Well-designed study |
| Sumer | 2003 | 12675701 | Within 24h of symptom onset | Continuous | 180-d independent (mRS 1-2) dependent (mRS 3 -6) | 266 | NA | p=0.09 | Moderate | Study attrition: moderate risk of bias (ROB), 54 patients excluded due to incomplete follow-up. 34 patients died within six months; outcome measurement: moderate ROB, unclear evaluation method for mRS scores at six months. |
| Jorgenson | 2020 | 10512899 | admission | Continuous | good functional outcome = BI ≥ 50 points | 84 | NA | Univariate p=0.09 Multivariate p=0.12 | Low | Well-designed study |
| Sacco | 1994 | 8164815 | admission | ≤140 mg/dl, n=193 >140 mg/dl, n=123 | 30-d mortality 1-y mortality 5-y mortality | 323 | NA | p<0.01 | Low | Well-designed study |
| Skafida | 2018 | 31008348 | measured at admission, and thereafter three times per day before meals | Continuous | 90-d mortality | 1269 | NA | HR 1.010, 95% CI 1.007-1.013, p<0.001 | Low | Single-center study, lack of standardized therapy, inability to control for drug use, and limitations in causality determination. |
| Zietemann | 2016 | 31008267 | admission and the next morning | Continuous | 90 day mRS | 422 | NA | Nonlinear relationship | Moderate | Patients with and without diabetes showed significant differences; small group of diabetic patients with only n=81 (19%). |
| Henden | 2018 | 30117146 | admission | Continuous | 90-d good outcome = mRS 0-2 | 198 | n=78 (39%) | OR 1.203, 95% CI 0.992-1.459, p=0.060 | Low | All low |
| Broocks | 2019 | 31667625 | admission | Continuous | 90 day mRS dichotomized 0-2 vs. 3-6 | 178 | n=69 (39%) mRS 0-2 at 90d  n=109 (61%) mRS 3-6 at 90d | OR 0.97, 0.96-0.99, p<0.001 | High | Study had missing necessary information, particularly regarding data and protocol. However, other sections were well-documented and detailed. |
| Dong | 2019 | 31478125 | Early morning after overnight fasting | Continuous | 90 day mRS dichotomized 0-2 vs. 3-6 | 206 | mRS <3, n=128  mRS ≥3, n=78 | AOR 1.41, 95% 1.18-1.67, p<0.001 | Low | Well-designed study |
| Wnuk | 2020 | 32201103 | the day following admission | Continuous | 90-d neurological outcome mRS | Whole group: n=181  Diabetics: n=42  Non-diabetics: n=139 | FG < 5.5 mmol/L: n=96 (34 patients, 35.4%)  FG > 5.5 mmol/L: n=43 (26 patients, 61.9%) | p=0.004 | Low | Well-designed study |
| Yao | 2016 | 27494527 | admission | Continuous | 180-d mRS >2 vs 2 or less | 2862 | 9.80% | OR 1.21(1.07–1.37) , p = 0.002 | Low | Well-designed study |
| Kim | 2016 | 26658447 | Admission value | Continuous | 90-d excellent functional outcome = mRS 0-1 | 34 | 23.77% | OR 0.87 per 10mg/dl (0.75 -1.00), p = 0.054 | Low | The study was limited by its retrospective nature as a post hoc analysis of a randomized controlled trial. It could only establish associations, not causation. The use of the TIMI scale for reperfusion measurement introduced potential measurement discrepancies. Insulin treatment in hyperglycemic patients was not investigated, highlighting the need for further research. The study's statistical power was limited due to its moderate sample size |
| Kim | 2016 | 26658447 | Admission value | Continuous | 90-d good functional outcome = mRS 0-2 | 46 | 32.17% | OR 0.93 per 10mg/dL (0.831.04), p =0.19 | Low | The study was limited by its retrospective nature as a post hoc analysis of a randomized controlled trial. It could only establish associations, not causation. The use of the TIMI scale for reperfusion measurement introduced potential measurement discrepancies. Insulin treatment in hyperglycemic patients was not investigated, highlighting the need for further research. The study's statistical power was limited due to its moderate sample size |
| Kim | 2016 | 26658447 | Admission value | Continuous | 90-d mortality | 36 | 25.17% | OR 1.02 per 10mg/dl (0.93-1.10) p=0.71 | Low | The study was limited by its retrospective nature as a post hoc analysis of a randomized controlled trial. It could only establish associations, not causation. The use of the TIMI scale for reperfusion measurement introduced potential measurement discrepancies. Insulin treatment in hyperglycemic patients was not investigated, highlighting the need for further research. The study's statistical power was limited due to its moderate sample size |
| Kim | 2016 | 26658447 | Admission (hyperglycemia binary >140) | Continuous | 90-d excellent functional outcome = mRS 0-1 | 34 | 23.77% | OR 0.22 (0.06-0.82, p = 0.02) | Low | The study was limited by its retrospective nature as a post hoc analysis of a randomized controlled trial. It could only establish associations, not causation. The use of the TIMI scale for reperfusion measurement introduced potential measurement discrepancies. Insulin treatment in hyperglycemic patients was not investigated, highlighting the need for further research. The study's statistical power was limited due to its moderate sample size |
| Kim | 2016 | 26658447 | Admission (hyperglycemia binary >141) | Continuous | 90-d good outcome = mRS 0-2 | 46 | 32.17% | OR 0.46 (0.17 - 1.30), p 0.14 | Low | The study was limited by its retrospective nature as a post hoc analysis of a randomized controlled trial. It could only establish associations, not causation. The use of the TIMI scale for reperfusion measurement introduced potential measurement discrepancies. Insulin treatment in hyperglycemic patients was not investigated, highlighting the need for further research. The study's statistical power was limited due to its moderate sample size |
| Kim | 2016 | 26658447 | Admission (hyperglycemia binary >142) | Continuous | 90-d mortality | 36 | 25.17% | 0.81 (0.32 - 2.03) p=0.65 | Low | The study was limited by its retrospective nature as a post hoc analysis of a randomized controlled trial. It could only establish associations, not causation. The use of the TIMI scale for reperfusion measurement introduced potential measurement discrepancies. Insulin treatment in hyperglycemic patients was not investigated, highlighting the need for further research. The study's statistical power was limited due to its moderate sample size |
| Lima | 2014 | NA | admission | Continuous | 180-d good outcome = mRS 2 or more | 56 | 44.40% | 0.99 (0.98-1.00) , p=0.08 | Low | Well-designed study |
| Arnold | 2014 | 22973986 | admission | Continuous | poor outcome mRS 3-6 | 199 | 51% | OR 3.03, 95% CI 1.45–6.34, p = 0.003 | Low | Limitations of this study include its observational monocentre design, small sample size hindering sub-group analysis of diabetic patients, lack of routine HbA1C measurement for undiagnosed diabetes cases, heterogeneous protocol potentially affecting outcomes, and failure to assess glucose values at 24 and 48 hours, limiting understanding of persistent hyperglycemia effects. |
| Akhtar | 2019 | 30545720 | admission | 56.00% | 90-d mRS 3-6 | 2961 | 24.00% | OR: 1.374 (1.022 1.847), p=0.035 | Moderate | Prospective study, 619 patients |
| Gattringer | 2019 | 30580732 | admission | 24.57% | 7-d mortality | 77653 | 2% | OR: 1.22 (1.08–1.38) | Moderate | 77653 patients, prospective multicenter sutdy |
| Laible | 2017 | 29084408 | admission | 19.40% | 90-d mRS ≥ 3 | 505 | 66.52% | OR: 2.54 (1.32–4.89) p=0.005 | Moderate | Prospective, 2010-2016, 505 patients |
| Wang | 2019 | 30539756 | before and after mechanical thrombectomy | 100% | 90-d mortality | 321 | 19.63% | OR: 3.80 (1.31-11.06) p=.014 | Moderate | 321 participants, retrospective study |
| Li | 2019 | 30396839 | admission | 28.50% | 90-d mRS 3-6 | 685 | 35.04% | OR: 2.11 (1.37-3.26) p=.001 | Moderate | Retrospective study, 685 participants, 2013-2024 |
| Li | 2019 | 30396839 | admission | 28.50% | AF | 685 | 29.80% | NR | Moderate | Retrospective study, 685 participants, 2013-2038 |
| Urbanek | 2018 | 30390631 | admission | 31.30% | 1-y mRS>2 | 1370 | 52.34% | OR:1.8 (1.3–2.5) p< 0.01 | Moderate | 143 patients, retrospective, 2006-2010 |
| Kimura | 2011 | 21397255 | admission | 61.20% | Ischemic lesion volume < 40 | 110 | 44.55% | OR: 2.9 (1.1–7.6) p=0.0341 | Moderate | 110 participants, prospective study, 2006-2010 |
| Kimura | 2011 | 21397255 | admission | 61.20% | Ischemic lesion volume < 80 | 110 | 55.45% | OR: 3.2 (1.1–9.0) p=0.0262 | Moderate | 110 participants, prospective study, 2006-2010 |
| Natarajan | 2011 | 21351835 | admission | 32.90% | 90-d mRS 3-6 | 614 | 61.10% | OR: 5.232 (3.079–8.893) p<0.001 | Moderate | prospective, multicenter, 174 patients |
| Natarajan | 2011 | 21351835 | 48 hours after admission | 29% | 90-d mRS 3-6 | 614 | 61.10% | OR: 2.021 (1.239–3.297) p=0.005 | Moderate | prospective, multicenter, 174 patients |
| Natarajan | 2011 | 21351835 | admission | 32.90% | 90-d mortality | 614 | 30.80% | OR: 5.031 (2.859–8.854)p<0.001 | Moderate | prospective, multicenter, 174 patients |
| Natarajan | 2011 | 21351835 | 48 hours after admission | 29% | 90-d mortality | 614 | 30.80% | OR: 2.273 (1.336–3.869) p=0.002 | Moderate | prospective, multicenter, 174 patients |
| Mustanoja | 2011 | 21106955 | admission | 100% | 90-d mRS, 0-2 | 957 | 58% | OR: 1.14 (1.07 - 1.22) | Moderate | Retrospective, 957 participants, 1995-2011 |
| Naganuma | 2011 | 21088392 | admission | 100% | 90-d mRS 0-1 | 578 | NA | OR: 0.91 (0.84–0.99) p=0.024 | Moderate | Retrospective, multicenter, 578 patients, 2005-2011 |
| Naganuma | 2011 | 21088392 | admission | 100% | 90-d mRS 4-6 | 578 | NA | OR: 1.08 (1.01–1.17) p=0.033 | Moderate | Retrospective, mulicenter, 578 patients, 2005-2011 |
| Appel | 2011 | 21079398 | admission | 32.20% | 1-year mortality | 784 | 21% | NR | High | prospective study, 784 participants, 2001-2007 |
| Putaala | 2011 | 21079397 | 48 hours after admission | 20.60% | 90-d mRS 3-6 | 851 | 40.50% | OR: 2.17 (1.40–3.38)p<0.05 | Moderate | Retrospective, 1998-2008, 851 patients |
| Putaala | 2011 | 21079397 | baseline and 48 hours after admission | 16.10% | 90-d mRS 3-6 | NA | 40.50% | OR: 2.33 (1.41–3.86) p<0.05 | Moderate | Retrospective, 1998-2008, 851 patients |
| Putaala | 2011 | 21079397 | 48 hours after admission | 20.60% | 90-d mortality | 851 | 9.30% | OR: 3.13 (1.56–6.27) p<0.05 | Moderate | Retrospective, 1998-2008, 851 patients |
| Putaala | 2011 | 21079397 | baseline and 48 hours after admission + baseline | 16.10% | 90-d mortality | 851 | 9.30% | OR: 6.63 (3.25–13.54) p<0.05 | Moderate | Retrospective, 1998-2008, 851 patients |
| Putaala | 2011 | 21079397 | 48 hours after admission | 20.60% | sICH | 851 | NA | OR: 1.89 (1.04–3.43) p<0.05 | Moderate | Retrospective, 1998-2008, 851 patients |
| Putaala | 2011 | 21079397 | baseline and 48 hours after admission | 16.10% | sICH | 851 | NA | OR: 3.02 (1.68–5.43) p<0.05 | Moderate | Retrospective, 1998-2008, 851 patients |
| Lima | 2010 | 20829514 | admission | 16.33% | 180-d mRS ≤ 2 | 196 | 41.84% | OR: 0.31 (0.01 to 0.98) P=0.046 | Moderate | Prospective study, 196 participants |
| Fuentes | 2010 | 20724713 | 48 hours within stroke onset | 24.58% | 90-d mRS>2 | 476 | 56.20% | OR: 4.76 (2.20 –10.29) | High | 476 participants, prospective study |
| Wei | 2010 | 20651267 | admission | 25% | 1-y mRS | 4782 | 29.97% | OR: 1.42 (1.20–1.68) | Moderate | Prospective, 4782 participants, 2006 |
| Ryu | 2010 | 20627296 | admission - 48 hours after | 100% | 1-year mortality | 1067 | 16.50% | HR: 1.10 (1.03–1.18) | Moderate | 1072 patients, prospective study, 2002-2007 |
| Ryu | 2010 | 20627296 | admission and 48 hours after admission | 100% | 1-year mortality | 1067 | 10.60% | HR: 1.13 (1.03–1.24) | Moderate | 1072 patients, prospective study, 2002-2007 |
| Spengos | 2010 | 20482604 | admission | 7.90% | 10-year composite cardiovascular event | 253 | 30.40% | HR: 3.20 (1.39–7.37), p<0.01 | Moderate | 253 participants, 1999-2008, prospective study |
| Hao | 2010 | 20332640 | admission | 11.90% | Reduced eGFR | 1758 | 26.30% | OR: 1.411 (1.012–1.967) p=0.043 | Moderate | Prospective, 1758 participants, 2002-2008 |
| Caso | 2010 | 20088729 | admission | 20% | 90-d mRS 3-6 | 1136 | 40.05% | OR: 2.10; (1.38–3.19) | Moderate | 2006-2007,1136 participants, prospective study |
| Kong | 2010 | 20029199 | admission | 21.70% | 1-year disability | 2774 | 34.50% | OR: 1.56 (1.01–2.39) | Moderate | 2002-2007, prospectie study, 2774 participants |
| Koton | 2010 | 20016218 | admission | 100% | 30-d mortality | 1079 | 9.90% | HR: 1.003 (1.001–1.006) p=0.03 | Moderate | 1079 participants, prospective |
| Hannon | 2010 | 19893311 | admission | 10.21% | 90-d mRS | 568 | NA | NA |  | 568 participants, prospective study, 2005-2006 |
| Jovanovic | 2009 | 19647928 | admission | 100% | 90-d mRS 0-1 | 156 | 35.08% | NA | High | Prospective study, 2006-2008, 156 participants |
| Stroud | 2009 | 19602474 | admission | 25.40% | 90-d OHS 0-1 | 673 | 40.56% | NA | Moderate | Retrospective analysis, 673 participants, 2002-2014 |
| Kissela | 2009 | 19109548 | admission | 40.60% | 90-d mRS | 451 | 2.6 average | B: 0.27 (0.02–0.51) p=0.031 | High | Retrospective, 451 patients, 1990 |
| Fuentes | 2009 | 19095970 | Admission and 48 hours after admission | 100% | 90-d mRS >2 | 476 | 63.30% | OR: 2.734 (1.425–5.244), p=0.002 | Moderate | 476 participants, prospective study |
| Fuentes | 2009 | 19095970 | admission | 24.80% | 90-d mRS >2 | 476 | 63.30% | NA | Moderate | 476 participants, prospective study |
| Khartionova | 2009 | 19018138 | admission | 17.19% | admission Hyperdense middle cerebral artery sign | 10023 | 19% | B: -0.222, p=0.011 | High | 1023 participants, prospective, 2002-2006 |
| Stead | 2009 | 18357419 | admission | 16.78% | 90-d mortality | 447 | 17.67% | HR: 1.8 (1.1–2.9), P = 0.012 | High | 447 participants, 2001-2004 |
| Stead | 2009 | 18357419 | admission | 17.44% | 90-d mortality | 447 | 17.67% | HR: 2.0 (1.1–3.3) P = 0.004) | High | 447 participants, 2001-2004 |
| Wahlgren | 2008 | 18927461 | admission | 12% | 90-d mRS 0-2 | 6947 | 50% | OR: 0.75 (0.62 - 0.91) | Moderate | 6947 participants, review of RCT, 2002-2006 |
| Yong | 2008 | 18703813 | 24 hours after admission | 9% | 90-d mRS, 0-2 | 587 | 58% | OR: 0.40 (0.20–0.78) | Moderate | 748 participants, patient sample from randomized controlled trial |
| Yong | 2008 | 18703813 | admission and 24 hours after admission | 8% | 90-d mRS 0-2 | 587 | 58% | OR: 0.36 (0.17–0.73) | Moderate | 748 participants, patient sample from randomized controlled trial |
| Yong | 2008 | 18703813 | 24 hours after admission | 9% | 90-d mortality | 587 | 9.54% | OR: 5.99 (2.51–14.2) | Moderate | 748 participants, patient sample from randomized controlled trial |
| Yong | 2008 | 18703813 | admission and 24 hours after admission | 8% | 90-d mortality | 587 | 9.54% | OR: 7.61 (3.23–17.90) | Moderate | 748 participants, patient sample from randomized controlled trial |
| Yong | 2008 | 18703813 | admission and 24 hours after admission | 8% | 30-d BI | 587 | 43.40% | OR: 0.27 (0.12–0.62) | Moderate | 748 participants, patient sample from randomized controlled trial |
| Ovbiagele | 2008 | 18583560 | admission | 100% | 90-d mRS ≥ 2 | 659 | 16.55% | NA | High | 659 participants, prospective study of RCT |
| Ovbiagele | 2008 | 18583560 | admission | 100% | 90-d mRS ≥ 2 | 659 | 16.55% | NA | High | 659 participants, prospective study of RCT |
| Rallidis | 2008 | 18093263 | admission | 29.40% | In-hospital mortality | 231 | 6.50% | RR: = 6.05, (1.7–21.5) P = 0.001 | Moderate | 234 participants, 2002-2006, prospective study |
| Basile | 2008 | 17825846 | maximum impairment | 20.05% | 90-d mRS 2-5 | 4190 | 78.40% | OR: 1.40 (1.07–1.83) p=0.012 | Moderate | 4191 participants, 1993 |
| Roquer | 2007 | 18004645 | admission | 100% | In-hospital mortality | 1527 | 12.90% | HR: 1.036 (1.008–1.065), p=0.012 | Moderate | 1997-2005, 1527 participants, prospective study |
| Roquer | 2007 | 17502469 | admission | 100% | 30-d mortality | 1527 | 13.80% | OR: 1.07 (1.02-1.12) p=.007 | Moderate | 1997-2005, 1527 participants, prospective study |
| Ryu | 2017 | 28008000 | admission | 26.3% | 90-d mRS | 5035 | 100% | OR 1.22 (1.08-1.38); P=0.002 | Moderate | 5035 patients from 8005 initially evaluated from 2011-2012; many post-hoc analyses conducted |
| Ryu | 2017 | 28008000 | admission | 100% | 90-d mRS | 5035 | 100% | OR 1.07 (1.05-1.09); p<0.001 | Moderate | 5035 patients from 8005 initially evaluated from 2011-2012; many post-hoc analyses conducted |
| Kim | 2018 | 30355207 | admission | 100% | 90-d mRS 0-2 | 309 | 49.2% | OR 0.95 (0.89-1.00); p=0.06 | Moderate | 308 patients from three interventional studies using the Solitaire stent retriever; LVO only, undergoing endovascular therapy; prospectively collected data, retrospectively analyzed |
| Kim | 2018 | 30355207 | admission | 100% | 90-d mortality | 309 | 14.2% | OR 0.99 (0.93-1.06); p=0.59 | Moderate | 308 patients from three interventional studies using the Solitaire stent retriever; LVO only, undergoing endovascular therapy; prospectively collected data, retrospectively analyzed |
| Demeestere | 2018 | 30355098 | admission | 22.40% | 90-d mRS 0-2 | 156 | 58.9% | OR 0.54 (0.21-1.41); p=0.21 | High | 156 patients, retrospectively analyzed, but prospectively enrolled in the CRISP study evaluating LVO in the anterior circulation who underwent endovascular therapy and had successful reperfusion |
| Nagel | 2018 | 29777016 | admission | 100% | 90-d mRS 2-6 | 1480 | 52.9% | NA | Moderate | 1480 patients from the alteplase arm of the ENCHANTED trial |
| Nagel | 2018 | 29777016 | admission | 100% | 90-d mRS 3-6 | 1480 | 37.8% | NA | Moderate | 1480 patients from the alteplase arm of the ENCHANTED trial |
| Nagel | 2018 | 29777016 | admission | 100% | 90-d mortality | 1480 | 9.8% | NA | Moderate | 1480 patients from the alteplase arm of the ENCHANTED trial |
| Dargazanli | 2018 | 29626134 | admission | 19.7% | 90-d mRS <3 | 290 | 50.3% | NA | Moderate | 290 patients with successful reperfusion (TICI 2b, 2C, 3 scores) from the ASTER study; anterior circulation stroke, underoing mechanical thrombectomy |
| Dargazanli | 2018 | 29626134 | admission | 19.7% | 90-d mortality | 290 | 14.8% | NA | Moderate | 290 patients with successful reperfusion (TICI 2b, 2C, 3 scores) from the ASTER study; anterior circulation stroke, underoing mechanical thrombectomy |
| Xue | 2017 | 28978089 | admission | 28.80% | 90-d mRS >2 | 438 | 65.7% | OR 1.29 (0.94-2.03); p=0.208 | Moderate | 438 patients prospectively enrolled from 2015-2016 |
| Xue | 2017 | 28978089 | admission | 100.00% | 90-d mRS >2 | 438 | 65.7% | OR 1.21 (1.07-1.38); p=0.006 | Moderate | 438 patients prospectively enrolled from 2015-2016 |
| Ahn | 2017 | 28877565 | admission | 24% | 33-m mortality | 1692 | 19.6% | NR | Moderate | 1692 patients retrospectively included from a single hospital from 2007-2011; all with ECG findings and troponin |
| Ricciardi | 2014 | 24851928 | admission | 100% | 90-d mRS <3 | 159 | 46.5% | OR 0.99 (0.98-1.01); p=0.216 | Moderate | 159 patients in a prospective registry at a single hospital with LVO, presenting within 12 hours of onset and evaluated using the IPAQ assessing pre-stroke physical activity |
| Wang | 2014 | 24789365 | admission | 39.9% | mortality, discharge | 326 | 11.7% | NR | High | 326 patients, first-ever stroke, presenting to a single hospital, with vitamin D levels from 2012-2013 |
| Yoo | 2014 | 24747428 | admission | 100.00% | 90-d mRS >2 | 207 | 62.8% | OR 1.021 (1.010–1.032); p<0.001 | Moderate | 207 consecutive patients, retrospective, single stroke center, primarily evaluating glucose and associated stroke outcomes; all patients received IV alteplase within 3 hours |
| Yoo | 2014 | 24747428 | admission | 100.00% | 90-d mRS >2 | 207 | 62.8% | OR 1.007 (1.000–1.014); p=0.045 | Moderate | 207 consecutive patients, retrospective, single stroke center, primarily evaluating glucose and associated stroke outcomes; all patients received IV alteplase within 3 hours |
| Yoo | 2014 | 24747428 | admission | 100.00% | 90-d mortality | 207 | 15.9% | OR 1.015 (1.005–1.025); p=0.002 | Moderate | 207 consecutive patients, retrospective, single stroke center, primarily evaluating glucose and associated stroke outcomes; all patients received IV alteplase within 3 hours |
| Yoo | 2014 | 24747428 | hospitalization | 100.00% | 90-d mortality | 207 | 15.9% | OR 1.012 (1.003–1.020); p=0.008 | Moderate | 207 consecutive patients, retrospective, single stroke center, primarily evaluating glucose and associated stroke outcomes; all patients received IV alteplase within 3 hours |
| Kim | 2014 | 24719133 | admission | 32.7% | 3-y mortality | 2820 | 17.7% | NA | Moderate | 2820 patients, 344 with early neurologic deterioration, which was the primary analysis; 200-2008 single hospital, excluding patients with recurrent stroke, alteplase receipt, and late >3 day presentation |
| Inoa | 2014 | 24686370 | admission | 25.00% | 90-d modified Barthel Index (mBI) <19 | 1569 | 58% | AC: OR 0.36 (0.25–0.52); p<0.001 \| PC: OR 0.35 (0.19–0.65); p=0.001 | Moderate | 1569 patients from a prospective registry, retrospectively analyzed; primary analysis was between anterior and posterior circulation stroke and associated outcomes/prognosis |
| Hao | 2014 | 24685995 | admission | 15.8% | 90-d mRS >2 | 215 | 36.3% | RR 0.69 (0.23-2.03); p=0.498 | Moderate | 215 patients from a prospective registry from 2009-2011 with severe intracranial stenosis or occlusion; included within 30 days of symptom onset; primary evaluation was on high blood pressure upon admission association with outcomes |
| Bettger | 2014 | 24666657 | admission | 31.1% | 90-d mRS 3-5 | 1965 | 36.0% | NA | High | 1965 patients with stroke who all survived at least 3 months; retrospective analysis of a prospective cohort; primary evaluation was socioeconomic status association with outcomes |
| Wu | 2014 | 24649851 | admission | 100% | 90-d mRS >1 | 1766 | NA | NA | High | 1766 ischemic stroke patients with A1c data and 1-year follow up data from the ACROSS (abnormal glucose regulation in patients with acute stroke across China Study) study in China |
| Wu | 2014 | 24649851 | admission | 100% | 90-d mRS >1 | 1766 | NA | NA | High | 1766 ischemic stroke patients with A1c data and 1-year follow up data from the ACROSS (abnormal glucose regulation in patients with acute stroke across China Study) study in China |
| Wu | 2014 | 24649851 | admission | 100% | 1-y mRS >1 | 1766 | NA | NA | High | 1766 ischemic stroke patients with A1c data and 1-year follow up data from the ACROSS (abnormal glucose regulation in patients with acute stroke across China Study) study in China |
| Wu | 2014 | 24649851 | admission | 100% | 1-y mRS >1 | 1766 | NA | NA | High | 1766 ischemic stroke patients with A1c data and 1-year follow up data from the ACROSS (abnormal glucose regulation in patients with acute stroke across China Study) study in China |
| Wu | 2014 | 24649851 | admission | 100.00% | 1-y mortality | 1766 | NA | NA | High | 1766 ischemic stroke patients with A1c data and 1-year follow up data from the ACROSS (abnormal glucose regulation in patients with acute stroke across China Study) study in China |
| Wu | 2014 | 24649851 | admission | 100% | 1-y mortality | 1766 | NA | NA | High | 1766 ischemic stroke patients with A1c data and 1-year follow up data from the ACROSS (abnormal glucose regulation in patients with acute stroke across China Study) study in China |
| Lee | 2014 | 24534029 | admission | 24.5% | 90-d mRS <3 | 110 | 83.6% | NA | High | 118 stroke patients with posterior circulation stroke within 6 hours of symptom onset, evaluating DWI lesion characteristics associations on outcomes |

**COLLATERAL STATUS**

| First author last name | Year | PMID | Timing of prognostic factor evaluation | Prevalence % of the predictor | Outcome(s) | Sample size for the outcome | Prevalence % of the outcome | Effect size | Overall risk of bias | Overall risk of bias: comments |
| --- | --- | --- | --- | --- | --- | --- | --- | --- | --- | --- |
| Kim | 2018 | 30355207 | admission | 100% | 90-d mRS 0-2 | 309 | 49.2% | NR | Moderate | 308 patients from three interventional studies using the Solitaire stent retriever; LVO only, undergoing endovascular therapy; prospectively collected data, retrospectively analyzed |
| Kim | 2017 | 28176499 | admission | 47.1% | 90-d mRS 0-2 | 104 | 44% | OR 2.74 (1.23–6.09); p=0.01 | Moderate | 104 patients from a prospective registry of 5558 patients, with endovascular recanalization treatment for LVO |
| Kim | 2017 | 28176499 | admission | 47.1% | 90-d mortality | 104 | 15% | OR 0.21 (0.06–0.79); p=0.02 | Moderate | 104 patients from a prospective registry of 5558 patients, with endovascular recanalization treatment for LVO |
| Lima | 2010 | 20829514 | admission | 100% | 180-d mRS ≤ 2 | 196 | 41.84% | OR: 1.93 (1.06 to 3.50) P=0.03 | Moderate | Prospective study with 196 participants, showing moderate influence of self-fulfilling prophecy on risk of bias. |
| Menon | 2015 | 25791716 | admission | NA | 90-d mRS 0-2 | NA | NA | p <0.05 | High | Study participation- high ROB, subgroup of RCT, not well described’; study attrition- moderate ROB, subgroup of RCT. RCT is well-known and assumptions can be made that f/u was good; prognostic factor measurement- moderate ROB, not enough information provided for 'collateral score'; outcome measurement- moderate ROB; study confounding- moderate rob; self-fulfilling prophecy- moderate |
| Rusanen | 2015 | 26352696 | admission | 44.2% | 90-d mRS 0-2 | NA | NA | OR 4.3, 95% CI 2.5-7.5, p <0.001 | High | Study attrition-moderate rob, no info on loss to f/u; prognostic factor measurement- unknown prevalence of outcome; outcome measurement- moderate ROB, study confounding- moderate ROB; statistical analysis and reporting- moderate ROB, unknown prevalence of outcome; self-fulfilling prophecy- moderate, unknown prevalence of mRS including death, no mention of WDLST |
| Sillanppa | 2015 | 25454401 | admission | NA | 90-d mRS 0-2 | 54 | 52% | OR 3.9 (1.1-13.9) p = 0.03 | Moderate | Study attrition- only one loss to f/u; prognostic factor measurement- moderate ROB; statistical analysis and reporting- moderate ROB; self-fulfilling prophecy- moderate ROB |
| Liebeskind | 2014 | 24876081 | admission | Grade n: 0 = 19, 1 = 53, 2 = 108, 3 = 88, 4 = 8. | median NIHSS at day 7/discharge | 104 | NA | P<0.001 | Moderate | Study attrition had a high risk of bias due to the lack of information about loss to follow-up and the unidentified individuals without available imaging (123/434). The outcome measurement had a moderate risk of bias as the method for assessing the 90-day modified Rankin Scale (mRS) was unclear. The presence of a self-fulfilling prophecy also posed a moderate risk. |
| Liebeskind | 2014 | 24876081 | admission | Grade n: 0 = 19, 1 = 53, 2 = 108, 3 = 88, 4 = 8. | 90-d mortality | 51 | NA | P<0.001 | Moderate | Study attrition had a high risk of bias due to the lack of information about loss to follow-up and the unidentified individuals without available imaging (123/434). The outcome measurement had a moderate risk of bias as the method for assessing the 90-day modified Rankin Scale (mRS) was unclear. The presence of a self-fulfilling prophecy also posed a moderate risk. |
| van Seeters | 2015 | 26358136 | at presentation | NA | 90-d poor outcome mRS 3-6 | 1374 | 501 (36%) | AOR 1.65 (95% CI 0.95-2.88) | Moderate | data from multicenter prospective study at 14 centers in Netherlands; split cohort for derivation/validation 60/40%; excellent analysis with discrimination AND 2 different calibration measures reported; 6% incomplete data and 1% missing 90d mRS excluded; no imputation of missing data; no word on WLST |
| Tong | 2017 | 28864854 | within 4.5 hours of onset | NA | 90-d mRS 0-2 | 135 | 48% | Recanalized patients: OR = 2.86 (95% CI), AOR = 3.67 (95% CI). | Moderate | New collateral scoring system shows potential. Some medical aspects are challenging to fully comprehend. |
| Kucinski | 2003 | 12525948 | mean time from symptom onset to initial CT was 2.1h | Good collateralization rates: Carotid: 46.4%, Internal carotid 46.2% Distal Middle Cerebral: 30.8% Middle Cerebral Trunk: 78.0% | 90-d good outcome = Barthel index > 90 | 111 | Favorable outcome: 40% good collateralization vs. 15.3% without. | OR 5.8, 95% CI 1.3-26.7, p=0.02 | Moderate | Outcome measurement: moderate risk of bias, unclear evaluation method for BI after 3 months. |
| Toni | 1997 | 8996480 | following initial CT scan one hour after hospitalization | NA | Improving, stable, and deteriorating defined by CNS scale. Increase or decrease of 1 point within first 48 hours of admission on Canadian Neurological Scale (CNS) indicates early improvement or deterioration. | 152 | Collateral blood supply present in:  7 out of 9 patients who improved (78%)  18 out of 36 patients who remained stable (50%)  7 out of 16 deteriorating patients (44%) | p<0.05 | Low | Well-designed study with minimal risk of bias |
| Park | 2020 | 32323503 | admission | NA | 90-d mRS 0-2 | 136 | Good outcome: n=35 (25.7%)  Poor outcome: n=101 (74.3%) | OR 14.130, 95% CI 2.264-88.212 p=0.005 | Low | Well-designed study with minimal risk of bias |
| Pikija | 2018 | 30353493 | pre-procedural CT angiography | 29.3% | 90-d mRS 0-2 | 174 | Good outcome  n=83 (50%) | OR 5.29, 95% CI 1.48-18.9, p=0.011 | Low | Well-designed study with minimal risk of bias |
| Wang | 2020 | 32484415 | Within 24 hrs of onset | NA | Menon score | 119 | Multiphase Menon score in good outcome: 4.  Multiphase Menon score in poor outcome: 3. | AUC = 0.72, 95%  Sensitivity: 68.4%  Specificity: 67.7%  OR: 3.04, 95% CI 1.61-5.73, p=0.001. | Low | Well-designed study with minimal risk of bias |
| Madelung | 2017 | 28905995 | admission | 29% | 90-d mRS 0-2 | 187 | poor collateral status mRS = 4 (3-6)  good collateral status mRS = 2 (1-4)  p<0.0001 | Poor collateral status OR 0.27, 95% CI 0.09-0.86 p=0.026 | Moderate | Protocol for evaluating mRS outcome not specified. |
| Madelung | 2017 | 28905995 | admission | 29% | 1-y mortality | 187 | 40.9% of patients with poor collateral  18.2% of the remaining population | Poor collateral  HR 4.3, 95% CI 1.52-12.4 | Moderate | Protocol for evaluating mRS outcome not specified. |
| Broocks | 2019 | 31667625 | Within 6 hrs of symptoms onset | NA | 90-d mRS 0-2 | 178 | N=69 (39%) mRS 0-2 at 90d  N=109 (61%) mRS 3-6 at 90d | Point increase in collateral  AOR 1.71, 1.10-2.67 p=0.020 | High | Study participation: moderate risk. Outcome measurement: moderate risk, lacks details. Functional outcome obtained from registry using mRS scores after 90 days, but registry and centre not specified. Statistical analysis and reporting: moderate, data not explicitly given. Supporting data available upon request. |
| Tang | 2019 | 30188358 | Within 6 hrs of symptoms onset | NA | 90-d poor outcome mRS 3-6 | 144 | favorable (n=81)  poor outcome (n=63) | p<0.001 | Low | Self-fulfilling prophecy- moderate, some patients received more conservative treatment, some patients treated with thrombolysis |

**EARLY NEUROLOGICAL IMPROVEMENT**

| **FIRST AUTHOR LAST NAME** | **YEAR OF PUBLICATION** | **PMID** | **Single/ Multicenter** | **Nature of revascularization** | **PROGNOSTIC FACTOR(S) EVALUATED** | **Timing of prognostic factor evaluation** | **Prevalence of the predictor** | **OUTCOME(S)** | **Sample size for the outcome** | **Prevalence of the outcome** | **EFFECT SIZE WITH 95% CI as reported in the study (False Positive Rate, Sensitivity, Specificity, Odds Ratio, Relative Risk)** | **OVERALL RISK OF BIAS FOR THE STUDY** | **Overall risk of bias: Comments** |
| --- | --- | --- | --- | --- | --- | --- | --- | --- | --- | --- | --- | --- | --- |
| Guenego | 2021 | 32812674 | Multicenter | Basilar thrombectomy +/- IV thrombolysis | NIHSS improvement >=8 or improvement to 0-1 | 24 hours after thrombectomy | 0.30 | At 3 months: mRS <=3, mRS<=2 | 237 | mRS<=3: 0.46; mRS<=2: 0.38 | OR 18.12 (95% CI 3.95–83.10). Among patients with ENI mRS<=3 in 84% and mRS<=2 in 73%. | Moderate | Participants moderate, Self-fulfilling prophecy moderate. |
| Wirtz | 2019 | 31860816 | Single center | All thrombectomy +/- IV thrombolysis | NIHSS improvement >=8 or absolute NIHSS <=10 | 24 hours after thrombectomy | NIHSS <=10: 0.33; NIHSS improvement by >=8: 0.45 | mRS<=2 at 3 months | 156 | 0.24 | For NIHSS <=10 at 24 hours: 22.53 (7.83–64.76). Among patients with NIHSS<=10 at 24 hours, mRS<=2 in 89%. Among patients with NIHSS <=8 at 24 hours, mRS<=2 in 62%. | Moderate | Participants moderate, Self-fulfilling prophecy moderate |
| Cai | 2022 | 34515554 | Multicenter | From clinical trial of Captor vs Solitaire stentrievers, all thrombectomy +/- IV thrombolysis | NIHSS improvement >=8 or improvement to 0-1 | 24 hours after thrombectomy | 0.23 | mRS<=2 at 3 months | 209 | 0.44 | OR=5.19 (2.16, 12.46). Among patients with ENI, mRS<=2 in 76%. | Moderate | Participants moderate, Self-fulfilling prophecy moderate |
| Agarwal | 2020 | 31836356 | Single center | IV tPA +/- thrombectomy | Delta NIHSS; Percent change in NIHSS | 24 hours after alteplase | Continuous variable | mRS<=1 at 3 months | 586 | 0.33 | Delta NIHSS: OR 1.27 (1.19-1.36) p<0.001. Percent change in NIHSS: OR 1.17 (1.12-1.22), p<0.001 | Moderate | Participants moderate, Self-fulfilling prophecy moderate |
| de Campos | 2020 | 32088468 | Single center | Anterior circulation thrombectomy +/- IV thrombolysis | Ultra-early ENI: NIHSS improvement >=4 or absolute NIHSS 0 or 1 | Immediately following thrombectomy in angio suite | 0.52 | mRS<=2 at 3 months | 296 | 0.59 | OR 4.61 (2.12–10.02). mRS <=1 in 70% of patients with ultra-early ENI. | Moderate | Participants moderate, prognostic factor assssment moderate, Self-fulfilling prophecy moderate |
| Bang | 2005 | 20396462 | Single center | MCA territory nonlacunar subacute stroke | NIHSS <=3 | 7 days following admission | 0.14 | mRS <=1 and Barthel Index >=95 at 6 months later. | 437 | 0.55 | OR 17.43 (5.29-57.40). Sensitivity 91% (87-94), specificity of 75% (68-81). | High | Participants moderate, prognostic factor assssment high, Self-fulfilling prophecy moderate |
| Sun | 2021 | 32488830 | Single center | Basilar thrombectomy +/- IV thrombolysis | NIHSS improvement >=8 or improvement to 0-1 | 24 hours after thrombectomy | 0.33 | At 3 months: mRS <=3, mRS<=2 | 187 | mRS<=3: 0.49; mRS<=2: 0.36 | mRS <=2: OR 19.45 (5.11–74.02). mRS <=3: OR 22.60 (6.82–74.89). Among patients with ENI mRS<=3 in 74% and mRS<=2 in 55%. | Moderate | Participants moderate, Self-fulfilling prophecy moderate |
| Soize | 2019 | 30472672 | Single center | All stentriever thrombectomy +/- IV thrombolysis | NIHSS improvement >=4 at 24 hours from thrombectomy; NIHSS improvement >=1 at 2 hours following thrombectomy | 2 hours and 24 hours after thrombectomy | NIHSS improvement >=4 at 24 hours from thrombectomy: 0.44; NIHSS improvement >=1 at 2 hours following thrombectomy: 0.53 | mRS<=2 at 3 months | 246 |  | mRS<=2 at 3 months in 80% of patients with NIHSS improvement >=1 at 2 hours (AUC 0.83), and 83% of patients with NIHSS improvement >=4 at 24 hours (AUC 0.93). | Moderate | Participants moderate, Self-fulfilling prophecy moderate |
| Weyland | 2021 | 33626903 | Single center | Anterior circulation thrombectomy +/- IV thrombolysis- successful revascularization only | Any improvement in NIHSS at the time of discharge | Discharge | 0.79 | At 3 months: mRS<=2, mRS<=1 | 549 with successful revascularization among 1146 attempted thrombectomies total | mRS <=2: 0.44; mRS<=1: 0.24 | Among patients with any improvement in NIHSS at discharge: mRS <=2 in 53% and mRS <=1 in 28% at 3 months | High | Participants high, prognostic factor evaluation high, Self-fulfilling prophecy moderate |
| Pu | 2018 | 29305274 | Multicenter | ACTUAL registry of all anterior circulation thrombectom +/- IV thrombolysisy in China | (Baseline NIHSS score − 24-hour/7-day NIHSS score)/ Baseline NIHSS score × 100% | Following thrombectomy: 24 +/- 6 hours and 7 +/- 2 days | ENI >12% at 24 hours: 0.5. ENI >41% at 7 days: 0.5. | mRS<=2 at 3 months | 568 | 0.51 | mRS<=2 at 3 months in: 74% of patients with ENI>12% at 24 hours (AUC 0.80); 81% of patients with ENI>41% at 7 days (AUC 0.88). | Moderate | Participants moderate, Self-fulfilling prophecy moderate |
| Cao | 2017 | 28117261 | Two-center | All thrombectomy +/- IV thrombolysis | 1. Percentage improvement: [(Baseline NIHSS score - 24h NIHSS score)/baseline NIHSS score] X 100%)  2. NIHSS improvement >=8 or improvement to 0-1 | 24 hours after thrombectomy | NIHSS improvement >=30% (Youden index) in 0.43 | mRS<=2 at 3 months | 129 | 0.41 | mRS>=2 at 3 months in 71% of patients with NIHSS improvement >=30% at 24 hours (AUC 0.77). AUC of NIHSS improvement >=8 or improvement to 0-1 was 0.68. | Moderate | Participants moderate, Self-fulfilling prophecy moderate |
| Lai | 2023 | 37780716 | Multicenter | All thrombectomy for onset <=6h +/- IV thrombolysis | NIHSS improvement >4 or improvement to 0-1 | 24 hours after thrombectomy | 0.31 | mRS<=2 at 3 months | 183 | 0.4 | mRS<=2 at 3 months in 81% of patients with ENI vs 22% without. Multivariate analysis- OR 15.56 (6.38–37.90), p<0.001 | Moderate | Participants moderate, Self-fulfilling prophecy moderate |

**HEMORRHAGIC TRANSFORMATION**

| First author last name | Year | PMID | Timing of prognostic factor evaluation | Prevalence % of the predictor | Outcome(s) | Sample size for the outcome | Prevalence % of the outcome | Effect size | Overall risk of bias | Overall risk of bias: comments |
| --- | --- | --- | --- | --- | --- | --- | --- | --- | --- | --- |
| di Poggio | 2019 | 30430315 | <4.5 hours after symptom onset | 0.44% | 90-d mRS ≤2 | 459 | 40.52% | OR: 22.9 (2.0–256.2) p=0.01 | High | 462 patients, January 2016 - December 2016, prospective study |
| England | 2010 | 21030711 | Subjects were included within 48 hours of stroke onset | NA | 90-d mRS >2 | 1297 | NA | 0.96 (0.62–1.49) P=0.868 | High | The study's limitations include potential bias in determining asymptomatic patients, selection bias in the trial's randomization process, and the lack of measurement of frailty despite its relevance in stroke outcomes. These factors may affect the accuracy of results and the generalizability of findings. |
| England | 2010 | 21030711 | Subjects were included within 48 hours of stroke onset | NA | 180-d mRS >2 | 1297 | NA | 1.06 (0.70–1.61) P=0.783 | Moderate | The study's limitations include potential bias in determining asymptomatic patients, selection bias in the trial's randomization process, and the lack of measurement of frailty despite its relevance in stroke outcomes. These factors may affect the accuracy of results and the generalizability of findings. |
| England | 2010 | 21030711 | Subjects were included within 48 hours of stroke onset | NA | 90-d Barthel Index <60 | 1297 | NA | 0.96 (0.62–1.48) P=0.843 | Moderate | The study's limitations include potential bias in determining asymptomatic patients, selection bias in the trial's randomization process, and the lack of measurement of frailty despite its relevance in stroke outcomes. These factors may affect the accuracy of results and the generalizability of findings. |
| England | 2010 | 21030711 | Subjects were included within 48 hours of stroke onset | NA | 180-d Barthel Index <60 | 1297 | NA | 1.31 (0.86–2.0) P=0.209 | Moderate | The study's limitations include potential bias in determining asymptomatic patients, selection bias in the trial's randomization process, and the lack of measurement of frailty despite its relevance in stroke outcomes. These factors may affect the accuracy of results and the generalizability of findings. |
| Zhang | 2014 | 24994827 | admission | 29.00% | 90-d mRS >2 | 129 | 57% | OR 3.23 (1.01–10.37); p=0.049 | Moderate | 129 consecutive, prospectively enrolled patients with stroke undergoing endovascular therapy at a single hospital from 2006-2013 |
| Kim | 2014 | 24719133 | 24-hour | 9.4% | 3-y mortality | 2820 | 17.7% | HR 1.341 (0.617-2.913); p=0.458 | Moderate | 2820 patients, 344 with early neurologic deterioration, which was the primary analysis; 200-2008 single hospital, excluding patients with recurrent stroke, alteplase receipt, and late >3 day presentation |
| Kim | 2014 | 24719133 | 24-hour | 9.4% | 30-d mortality | 2820 | 2.8% | OR 26.483 (10.806-64.902); p<0.001 | Moderate | 2820 patients, 344 with early neurologic deterioration, which was the primary analysis; 200-2008 single hospital, excluding patients with recurrent stroke, alteplase receipt, and late >3 day presentation |
| Bentes | 2017 | 29588974 | 24-hour | NA | mRS ≤2 at discharge | 129 | NA | p = 0.171 | Moderate | Prospective study, high missing data percentage. |
| Bentes | 2017 | 29588974 | 24-hour | NA | Discharge mortality | 129 | NA | P=1.000 | Moderate | Prospective study, high missing data percentage. |
| Bentes | 2017 | 29588974 | 24-hour | NA | 1-y mRS ≤2 | 129 | NA | p = 0.166 | Moderate | Prospective study, high missing data percentage. |
| Bentes | 2017 | 29588974 | 24-hour | NA | 1-y mortality | 129 | NA | p = 0.362 | Moderate | Prospective study, high missing data percentage. |

**INFARCT SIZE**

| First author last name | Year | PMID | Timing of prognostic factor evaluation | Prevalence % of the predictor | Outcome(s) | Sample size for the outcome | Prevalence % of the outcome | Effect size | Overall risk of bias | Overall risk of bias: comments |
| --- | --- | --- | --- | --- | --- | --- | --- | --- | --- | --- |
| Lin | 2017 | 28536175 | admission | Continuous variable | 90-d mRS >3 | 490 | Good- 79%, Poor- 21% | AOR 6.73, 95% 2-22.59, p=0.002 | Moderate | No mention of self-fulfilling or WLST, no mention of missing data approach |
| Lin | 2017 | 28536175 | admission | Continuous variable | 90-d NeuroQoL | 490 | NA | - Upper extremity (beta -0.95; P=0.38) - Lower extremity (beta -0.35; P=0.73) - Executive function (beta -0.97; P=0.35) - General concerns (beta -1.86; P=0.84) | Moderate | No mention of self-fulfilling or WLST, no mention of missing data approach |
| van Seeters | 2015 | 26358136 | at presentation | Continuous variable | 90-d poor outcome mRS 3-6 | 1374 | 36% | AOR 1.12 (95% CI 0.87-1.44) | Moderate | Data from multicenter prospective study at 14 centers in Netherlands; split cohort for derivation/validation 60/40%; excellent analysis with discrimination AND 2 different calibration measures reported; 6% incomplete data and 1% missing 90d mRS excluded; no imputation of missing data; no word on WLST |
| Henninger | 2014 | 24523039 | admission | Continuous variable | 90-d poor outcome mRS 3-6 | 34 | 29% | OR, 9.156; 95% CI, 3.191–26.270; P<0.001 | Moderate | Study attrition- moderate ROB, No mention of loss to f/u; outcome measurement- moderate ROB, not sure how 90 day mRS was measured (phone, in person etc.); self-fulfilling prophecy- moderate |
| Boers | 2018 | 29627794 | admission | Continuous variable | 90-d mRS 0-2 | 1665 | 39% | OR: 0.71, P=0.043 | Moderate | 1665 participants, review of trials |
| Sanak | 2011 | 20597864 | admission | Continuous variable | 90-d mRS 0-2 | 125 | 66.40% | OR: 0.981 (0.957–1.005), p=0.121 | Moderate | 125 participants, retrospective study, 2004-2008 |
| Silva | 2010 | 20733301 | admission, within 24 hours | Continuous variable | 180-d mRS ≥3 | 676 | 32.50% | OR 1.22 (1.15-1.31) P<0.01 | Moderate | The study acknowledges limitations, such as the absence of gold-standard tests to confirm ischemic lesion volumes and the exclusion of factors like post stroke depression, social support, biological effects of gender, and baseline frequency of silent brain injury, which may contribute to gender disparity in acute ischemic stroke outcomes. |
| Pan | 2010 | 20510983 | admission | Continuous variable | 180-d BI | 109 | 100% | OR 9.09 (2.03 to 16.16) p=0.012 | High | 109 participants, prospective study |
| Sanak | 2010 | 20127250 | 24 hours of admission | Continuous variable | 90-d mRS 3-6 | 157 | 36.94% | OR: 1.035 (0.984–1.089), p=0.180 | High | Retrospective,157 participants, 2004-2008 |
| Stroud | 2009 | 19602474 | admission | Continuous variable | 90-d mRS 0-2 | 673 | 40.56% | P<0.01 | Moderate | Retrospective analysis, 673 participants, 2002-2024 |
| Barrett | 2009 | 19443798 | 24 hours within symptom onset and day 5 | Continuous variable | 90-d mRS 0-2 | 169 | 54% | OR: 0.57 (0.37 to 0.88) | High | 169 participants, prospective study |
| Barrett | 2009 | 19443798 | 24 hours within symptom onset and day 5 | Continuous variable | 90-d Barthel Index | 169 | 54% | OR: 0.75 (0.56 to 1.01) | High | 169 participants, prospective study |
| Borsody | 2009 | 19390182 | 2 days within admission | Continuous variable | 1-y survival | 111 | 90.10% | HR: 3.9 (1.0–15.0), p<0.05 | Moderate | 111 participants, prospective study, 2002 |
| Fuentes | 2009 | 19095970 | admission | Continuous variable | 90-d mRS >2 | 476 | 63.30% | OR: 6.948 (3.369–14.331) p=0.000 | Moderate | Prospective study, multicenter, 476 participants, 2002-2006 |
| Khartionova | 2009 | 19018138 | admission | Continuous variable | admission Hyperdense middle cerebral artery sign(HMCA) | 10023 | 19% | B: 0.340, p<0.001 | High | 1023 participants, prospective, 2002-2006 |
| Arboix | 2008 | 18817678 | admission | Continuous variable | mortality, in-hospital | 2704 | 12.90% | OR: 2.33 (1.84-2.96) p<.001 | Moderate | 1265 participants, prospective study, 2004-2005 |
| Arboix | 2008 | 18817678 | admission | Continuous variable | mortality, in-hospital | 2704 | 12.90% | OR: 1.96 (1.33-2.89) p=0.001 | Moderate | 1266 participants, prospective study, 2004-2005 |
| Ryu | 2017 | 28008000 | admission | Continuous variable | 90-d mRS 0-2 | 5035 | 100% | OR 1.24 (1.19-1.29); p<0.001 | Moderate | 5035 patients from 8005 initially evaluated from 2011-2012; many post-hoc analyses conducted |
| Chen | 2018 | 30166435 | admission | Continuous variable | 90-d mRS 0-2 | 104 | 42.3% | OR 0.986 (0.972-1.001); p=0.061 | High | 104 consecutive patients with M1 occlusion ischemic stroke who could be evaluated for thrombus permeability based on conventional CTA |
| Laredo | 2018 | 29934530 | admission | Continuous variable | 90-d mRS >2 | 195 | NA | Significant relationship with particular area of brain | Moderate | Retrospective design, broad definition of poor functional outcome using modified Rankin Scale; potential bias towards specific brain areas. Difficulty in recruiting patients with strategic infarcts, but larger studies may enable lesion mapping analysis even in cases of small distributed infarcts. |
| Laredo | 2018 | 29934530 | admission | Continuous variable | 90-d mortality | 195 | NA | Significant relationship with particular area of brain | Moderate | Retrospective design, broad definition of poor functional outcome using modified Rankin Scale; potential bias towards specific brain areas. Difficulty in recruiting patients with strategic infarcts, but larger studies may enable lesion mapping analysis even in cases of small distributed infarcts. |
| Al-Ajlan | 2018 | 29170266 | admission | Continuous variable | 90-d mRS 0-2 | 206 | 35.9% | p=0.02 | Moderate | 206 prospectively enrolled patients in the REVASCAT trial; 103 received mechanical thrombectomy for anterior circulation, LVO |
| Liu | 2017 | 29201238 | admission | Continuous variable | 90-d mRS >2 | 116 | 34.5% | <0.001 | High | Retrospective study of 116 patients, who all received a follow up MRI and none received thrombolysis for initial stroke treatment |
| Kim | 2017 | 28176499 | admission | Continuous variable | 90-d mRS <3 | 104 | 44% | p=0.01 | Moderate | 104 patients from a prospective registry of 5558 patients, with endovascular recanalization treatment for LVO |
| Kim | 2017 | 28176499 | admission | Continuous variable | 90-d mortality | 104 | 15% | p=0.01 | Moderate | 104 patients from a prospective registry of 5558 patients, with endovascular recanalization treatment for LVO |
| Zhang | 2014 | 24994827 | admission | Continuous variable | 90-d mRS >2 | 129 | 57% | p=0.006 | Moderate | 129 consecutive, prospectively enrolled patients with stroke undergoing endovascular therapy at a single hospital from 2006-2013 |
| Kleine | 2017 | 28855390 | Admission | Continuous variable | 90-d mRS >2 | 322 | NA | AOR 8.618, 95% CI (2.409-30.828), p=0.001 | Moderate | moderate ROB due to implications of imaging selection, some patients lost to follow-up, and credibility/collection of mRS data at 90days |
| Dankbaar | 2017 | 28716981 | 48 h after admission | Continuous variable | 90-d mRS 0-2 | 242 | 56.1% | AOR1.19, 95% CI (1.10-1.30) | Low | Well-designed study |
| Sumer | 2003 | 12675701 | evaluation within 24h of symptom onset | Continuous variable | 180-d independence (mRS 1-2) | 250 | 10% | p<0.001 | Moderate | Study attrition- moderate ROB, 54 patients not included because follow-up was incomplete. 34 patients died by six months; outcome measurement- moderate ROB, does not say how the mRS scores were evaluated at six months; |
| Fukuda | 1999 | 10589798 | admission | Continuous variable | 90-d mRS after onset of stroke | 183 | NA | Spearman ρ = 0.557, p<0.0001 | Low | Well-designed study |
| Saver | 2020 | 9933262 | CT scans btwn days 6 to 11 | Continuous variable | 76-106-d favorable outcome = NIHSS score ≤ 1 | 191 | NA | r=0.54 | Low | Study evaluates infarct size but unclear why some patients' infarcts weren't visualized at 6-11 days. Data divided into "all infarcts" vs. "only visible infarcts." |
| Saver | 2020 | 9933262 | CT scans btwn days 6 to 11 | Continuous variable | 76-106-d favorable outcome = BI score of 60-100 | 191 | NA | r=0.43 | Low | Study evaluates infarct size but unclear why some patients' infarcts weren't visualized at 6-11 days. Data divided into "all infarcts" vs. "only visible infarcts." |
| Saver | 2020 | 9933262 | CT scans btwn days 6 to 11 | Continuous variable | 76-106-d favorable outcome GOS score ≤ 2 | 191 | NA | r=0.53 | Low | Study evaluates infarct size but unclear why some patients' infarcts weren't visualized at 6-11 days. Data divided into "all infarcts" vs. "only visible infarcts." |
| Saver | 2020 | 9933262 | CT scans btwn days 6 to 11 | Continuous variable | 76-106-d mortality | 191 | NA | r=0.31 | Low | Study evaluates infarct size but unclear why some patients' infarcts weren't visualized at 6-11 days. Data divided into "all infarcts" vs. "only visible infarcts." |
| Toni | 1997 | 8996480 | repeat CT scan in the first week after stroke | Continuous variable | CNS scale defines improvement, stability, or deterioration within 48 hours based on 1-point increase or decrease upon admission | 152 | For SMALL infarct:   - Improvement: 65% (22/34) - Stability: 39% (33/84) - Deterioration: 0% (4/34)   For MEDIUM infarct:   - Improvement: 26% (9/34) - Stability: 35% (28/84) - Deterioration: 31% (12/34)   For LARGE infarct:   - Improvement: 9% (3/34) - Stability: 27% (23/84) - Deterioration: 56% (19/34) | p=0.0000 | Low | Well designed study |
| Finocchi | 1996 | 8933227 | CT scan out at least 72 hours after onset of neurological deficit to confirm size and location of ischemic lesion | Continuous variable | mortality and disability as defined by Oxford Disability scale (ODS) evaluated on day 30  ODS 3-5 = disabled | 351 | 34% | P<0.001 | Low | Well-designed study |
| Pikija | 2018 | 30353493 | 24h and 7 days follow up scan | Continuous variable | 90-d mRS 0-2 | 174 | 50% | p<0.001 | Low | Well-designed study |
| Potreck | 2019 | 30887195 | admission | Continuous variable | 90-d mRS 0-2 | 131 | NA | OR 0.68, 0.49-0.94, p=0.021 | Low | Well-designed study |
| Wang | 2020 | 32484415 | within 24 hrs of onset | Continuous variable | 90-d mRS 0-2 | 119 | NA | p<0.001 | Low | Well-designed study |
| Dong | 2019 | 31478125 | within 48h of admission | Continuous variable | 90-d mRS 0-2 | 206 | NA | OR 1.41, 95% 1.18-1.67 p<0.001 | Low | Well-designed study |
| Yoo | 2020 | 22426317 | admission | Continuous variable | 90-d mRS 0-2 | 107 | 27 (25.2%) | - FIV of 40-50cm3 had the best accuracy (sensitivity: 74.1%-81.5%, specificity: 77.5%-85.0%) for identifying a good outcome. - FIV of 50 cm3 led to marked improvement in mRS scores. - FIV of 80-90cm3 showed high specificity (approximately 85%-90%) for predicting a poor outcome. |  | Excluded 25% of patients due to inadequate imaging. No significant differences in major variables and outcomes between included and excluded patients, except for increased congestive heart failure in excluded group. Reliability of lesion measurement and validation using pretreatment infarct volumes not assessed. |
| Yoo | 2020 | 22426317 | admission | Continuous variable | mortality | 107 | 30 (28.0%) | Larger FIV predicted mortality (P<0.0001) |  | Excluded 25% of patients due to inadequate imaging. No significant differences in major variables and outcomes between included and excluded patients, except for increased congestive heart failure in excluded group. Reliability of lesion measurement and validation using pretreatment infarct volumes not assessed. |
| Bucker | 2017 | 28351963 | 1 week after stroke | Continuous variable | 90-d mRS ordinal | 228 | NA | AOR 0.99, 95% CI 0.98-0.99, p<0.001 | Low | Smaller patient population compared to the full MR CLEAN population, potential bias due to the exclusion of patients who died before the 1-week follow-up imaging, difficulty in distinguishing true infarct progression from edema formation, use of CT instead of MRI imaging influencing lesion appearance, variability in the time window for follow-up scans, omission of contralateral hemisphere infarcts impacting lesion growth assessment, and strong collinearity between 24-hour NIHSS score and lesion volume affecting their independent associations with functional outcome. |
| Bucker | 2017 | 28351963 | 1 week after stroke | Continuous variable | 90-d mRS 0-2 | 228 | NA | AOR 0.97, 95% CI 0.96-0.99, p<0.001 | Low | Smaller patient population compared to the full MR CLEAN population, potential bias due to the exclusion of patients who died before the 1-week follow-up imaging, difficulty in distinguishing true infarct progression from edema formation, use of CT instead of MRI imaging influencing lesion appearance, variability in the time window for follow-up scans, omission of contralateral hemisphere infarcts impacting lesion growth assessment, and strong collinearity between 24-hour NIHSS score and lesion volume affecting their independent associations with functional outcome. |
| Bucker | 2017 | 28351963 | 24h after stroke | Continuous variable | 90-d mRS ordinal | 228 | NA | AOR 0.99, 95% CI 0.98-0.99, p<0.001 | Low | Smaller patient population compared to the full MR CLEAN population, potential bias due to the exclusion of patients who died before the 1-week follow-up imaging, difficulty in distinguishing true infarct progression from edema formation, use of CT instead of MRI imaging influencing lesion appearance, variability in the time window for follow-up scans, omission of contralateral hemisphere infarcts impacting lesion growth assessment, and strong collinearity between 24-hour NIHSS score and lesion volume affecting their independent associations with functional outcome. |
| Bucker | 2017 | 28351963 | 24h after stroke | Continuous variable | 90-d mRS 0-2 | 228 | NA | AOR 0.98, 95% CI 0.96-0.99, p<0.001 | Low | Smaller patient population compared to the full MR CLEAN population, potential bias due to the exclusion of patients who died before the 1-week follow-up imaging, difficulty in distinguishing true infarct progression from edema formation, use of CT instead of MRI imaging influencing lesion appearance, variability in the time window for follow-up scans, omission of contralateral hemisphere infarcts impacting lesion growth assessment, and strong collinearity between 24-hour NIHSS score and lesion volume affecting their independent associations with functional outcome. |
| Munsch | 2016 | 26585396 | admission | Continuous variable | 90-d mRS 0-2 | 428 | 43.2% | Beta −0.094, 95% CI −0.260 to 0.071 | Moderate | Highly selected patients were included, with only those who had an MRI within 24-72 hours of stroke and were assessed for functional and cognitive outcomes three months later. The study had great statistics but did not perform imputation for missing data. There was no mention of WLST. |
| Munsch | 2016 | 26585396 | admission | Continuous variable | 90-d MoCA (Good >25) | 428 | 46.4% | Beta −0.293, 95% CI −0.421 to 0.165 | Moderate | Highly selected patients were included, with only those who had an MRI within 24-72 hours of stroke and were assessed for functional and cognitive outcomes three months later. The study had great statistics but did not perform imputation for missing data. There was no mention of WLST. |
| Shi | 2016 | 26409718 | admission | Continuous variable | 1-y mRS 3-6 | 2168 | NA | AOR 2.08 (95% CI 1.51-2.87) | Moderate | A single Chinese hospital registry included AIS patients within 14 days of symptom onset, with a complete 12-month follow-up. There was no data on loss to follow up or imputation. The analysis was adequate, but there was no mention of WLST or how patients died. |

**NATIONAL INSTITUTE OF HEALTH STROKE SCALE (NIHSS)**

| First author last name | Year | PMID | Timing of prognostic factor evaluation | Prevalence % of the predictor | Outcome(s) | Sample size for the outcome | Prevalence % of the outcome | Effect size | Overall risk of bias | Overall risk of bias: comments |
| --- | --- | --- | --- | --- | --- | --- | --- | --- | --- | --- |
| Gao | 2016 | 27588095 | admission | Continuous variable | mortality | 619 | 16.50% | OR: 1.15 (1.04-1.27) | Moderate | Prospective study, 619 patients |
| Akhtar | 2019 | 30545720 | admission | Continuous variable | 90-d mRS 3-6 | 2961 | 24.00% | OR: 1.203 (1.171 1.236), p=0.001 | Moderate | Prospective study, 619 patients |
| Gattringer | 2019 | 30580732 | admission | Continuous variable | 7-d mortality | 77653 | 2% | OR: 4.97 (3.91–6.32) | Moderate | 77653 patients, prospective multicenter study |
| Gattringer | 2019 | 30580732 | admission | Continuous variable | 7-d mortality | 77653 | 2% | OR: 27.92 (22.45–34.71) | Moderate | 77653 patients, prospective multicenter study |
| Gattringer | 2019 | 30580732 | admission | Continuous variable | 7-d mortality | 77653 | 2% | OR: 92.33 (73.0–116.77) | Moderate | 77653 patients, prospective multicenter study |
| di Poggio | 2019 | 30430315 | admission | Continuous variable | 90-d mRS ≤2 | 459 | 40.52% | OR: 20.1 (1.1–387.4)p= 0.047 | High | 459 patients, January 2016 - December 2016, prospective study |
| di Poggio | 2019 | 30430315 | admission | Continuous variable | 90-d mRS ≤2 | 459 | 40.52% | OR: 20.1 (1.1–387.4) p=0.047 | High | 460 patients, January 2016 - December 2016, prospective study |
| Gory | 2018 | 29393092 | admission | Continuous variable | 90-d mortality | 117 | 41.90% | OR: 4.43 (1.46–13.39) p=0.008 | Moderate | 150 patients, prospective, 2016-2017 |
| Laible | 2017 | 29084408 | admission | Continuous variable | 90-d mRS ≥3 | 505 | 66.52% | OR:1.09 (1.05–1.14)p<0.001 | Moderate | Prospective, 2010-2016, 505 patients |
| Wang | 2019 | 30539756 | admission | Continuous variable | 90-d mortality | 321 | 19.63% | P < 0.01 | Moderate | 321 participants, retrospective study |
| Li | 2019 | 30396839 | admission | Continuous variable | 90-d mRS 3-6 | 685 | 35.04% | OR: 1.23 (1.18-1.27) p<.001 | Moderate | Retrospective study, 685 participants, 2013-2027 |
| Li | 2019 | 30396839 | admission | Continuous variable | AF | 685 | 29.80% | P < 0.01 | Moderate | Retrospective study, 685 participants, 2013-2041 |
| Boers | 2018 | 29627794 | admission | Continuous variable | 90-d mRS 0-2 | 1665 | 39% | p<0.001 | Moderate | 1665 participants, review of trials |
| Urbanek | 2018 | 30390631 | admission | Continuous variable | 1-y mRS>2 | 1370 | 52.34% | OR: 1.2 (1.1–1.2)p < 0.01 | Moderate | 144 patients, retrospective, 2006-2010 |
| Zhang | 2018 | 29053905 | admission | Continuous variable | 90-d mRS 0-2 | 158 | 29.70% | OR: 0.824 (0.750–0.904) p<0.001 | Moderate | 158 participants, retrospective analysis, 2009-2016 |
| Zhang | 2018 | 29053905 | admission | Continuous variable | 90-d mRS 0-2 | 158 | 29.70% | OR: 0.838 (0.726–0.968) p=0.016 | Moderate | 158 participants, retrospective analysis, 2009-2016 |
| Zhang | 2018 | 29053905 | admission | Continuous variable | 90-d mRS 0-2 | 158 | 29.70% | OR: 0.816 (0.669–0.994) p=0.044 | Moderate | 158 participants, retrospective analysis, 2009-2016 |
| Zhang | 2018 | 29053905 | admission | Continuous variable | 90-d mRS 0-2 | 158 | 29.70% | OR: 0.808 (0.674–0.968) p=0.021 | Moderate | 158 participants, retrospective analysis, 2009-2016 |
| Muscari | 2016 | 27186444 | admission | Continuous variable | 9-months mRS | 309 | 51.10% | P<0.0001 | Moderate | 151 patients, retrospective, 2006-2010 |
| Bhatia | 2011 | 21566239 | admission | Continuous variable | 90-d mRS≤ 2 | 251 | 46.61% | OR: 0.945 (0.90–0.989) p=0.04 | Moderate | NA |
| Kruetzelmann | 2011 | 21415399 | admission | Continuous variable | 90-d mRS 0-1 | 174 | 47.70% | OR: 0.861 (0.792–0.935) p=0.001 | Moderate | prospective, multicenter, 174 patients |
| Kruetzelmann | 2011 | 21415399 | admission | Continuous variable | 90-d mortality | 174 | NR | OR: 1.099 (0.980–1.231)p=0.105 | Moderate | prospective, multicenter, 174 patients |
| Kruetzelmann | 2011 | 21415399 | admission | Continuous variable | 90-d mRS 4-6 | 174 | 53.30% | OR: 1.234 (1.108–1.375),p=0.001 | Moderate | prospective, multicenter, 174 patients |
| Natarajan | 2011 | 21351835 | admission | Continuous variable | 90-d mRS 3-6 | 614 | 61.10% | OR: 6.146 (3.339–11.313) p<0.001 | Moderate | prospective, multicenter, 174 patients |
| Natarajan | 2011 | 21351835 | admission | Continuous variable | 90-d mortality | 614 | 30.80% | OR: 2.342 (1.463–3.751) p<0.001 | Moderate | prospective, multicenter, 174 patients |
| Mustanoja | 2011 | 21106955 | admission | Continuous variable | 90-d mRS 0-3 | 957 | 58% | OR: 1.21 (1.17 to 1.25) | Moderate | Retrospective, 957 participants, 1995-2013 |
| Naganuma | 2011 | 21088392 | admission | Continuous variable | 90-d mRS 0-1 | 578 | NR | OR: 0.91 (0.88–0.94) p<0.001 | Moderate | Retrospective, mulicenter, 578 patients, 2005-2012 |
| Naganuma | 2011 | 21088392 | admission | Continuous variable | 90-d mRS 4-6 | 578 | NR | OR: 1.11 (1.08–1.15) p<0.001 | Moderate | Retrospective, mulicenter, 578 patients, 2005-2017 |
| Naganuma | 2011 | 21088392 | admission | Continuous variable | 90-d mortality | 578 | NR | OR: 1.09 (1.04–1.15) p<0.001 | Moderate | Retrospective, mulicenter, 578 patients, 2005-2021 |
| Appel | 2011 | 21079398 | admission | Continuous variable | 90-d mortality | 784 | 21% | P < 0.01 | High | prospective study, 784 participants, 2001-2009 |
| Putaala | 2011 | 21079397 | admission | Continuous variable | 90-d mRS 3-6 | 851 | 40.50% | OR: 1.21 (1.17–1.25) p<0.05 | Moderate | Retrospective, 1998-2008, 851 patients |
| Putaala | 2011 | 21079397 | admission | Continuous variable | 90-d mortality | 851 | 9.30% | OR: 1.18 (1.12–1.24) p<0.05 | Moderate | Retrospective, 1998-2008, 851 patients |
| Putaala | 2011 | 21079397 | admission | Continuous variable | sICH | 851 | NR | OR: 1.06 (1.01–1.10)p<0.05 | Moderate | Retrospective, 1998-2008, 851 patients |
| Tei | 2011 | 20957383 | admission | Continuous variable | 90-d mRS 3-6 | 350 | 37.70% | OR: 1.368 (1.223–1.531) p<0.001 | Moderate | 351 participants, 1994-2009 |
| Alvarez-Perez | 2011 | 20954836 | admission | Continuous variable | mRS at discharge, >2 | 200 | 59.50% | OR: 1.560, (1.323–1.838) p < .0001 | High | 201 participants, prospective study |
| Soares | 2011 | 20688404 | admission | Continuous variable | 180-d mRS | 115 | 47.00% | OR: 1.522 (1.292–1.792) | High | Prospective, 115 participants, 2003-2006 |
| Sanak | 2011 | 20597864 | admission, within 24 hours | Continuous variable | 90-d mRS, 0-2 | 125 | 66.40% | OR: 0.764 (0.670–0.871), p=0.0001 | High | 125 participants, retrospective, 2004-2008 |
| Sanak | 2011 | 20597864 | admission | Continuous variable | 90-d mRS 0-2 | 125 | 66.40% | OR: 0.764 (0.670–0.871), p=0.0001 | Moderate | 125 participants, retrospective study, 2004-2008 |
| Sanak | 2011 | 20597864 | 24 hours after stroke onset | Continuous variable | 90-d mRS 0-2 | 125 | 66.40% | OR: 0.531 (0.413–0.683), p<0.0001 | Moderate | 125 participants, retrospective study, 2004-2008 |
| Muresan | 2010 | 21060010 | admission | Continuous variable | 90-d mRS | 120 | 36.67% | OR: 0.83 (0.76-0.91) P<0.001 | High | Retrospective analysis of prospective data, 2002-2007, 120 participants |
| Lima | 2010 | 20829514 | admission | Continuous variable | 180-d mRS ≤ 2 | 196 | 41.84% | OR: 0.75 (0.69 to 0.83) P<0.001 | Moderate | Prospective study, 196 participants |
| Silva | 2010 | 20733301 | admission | Continuous variable | 180-d mRS ≥3 | 676 | 32.50% | NA | Moderate | 679 participants, Prospective, 2003-2005 |
| Ryu | 2010 | 20627296 | day 2 after hospitalization | Continuous variable | 1-year mortality, all-cause | 1067 | 16.50% | HR: 1.06 (1.04–1.09) | Moderate | 1071 patients, prospective study, 2002-2007 |
| Ryu | 2010 | 20627296 | day 2 after hospitalization | Continuous variable | 1-year mortality, vascular death | 1067 | 10.60% | HR: 1.08 (1.05–1.10) | Moderate | 1077 patients, prospective study, 2002-2007 |
| Tafreshi | 2010 | 20538695 | admission | Continuous variable | 90-d mRS, 2-6 | 848 | 28.30% | P < 0.01 | High | Prospective study, 848 participants, 2001-2009 |
| Spengos | 2010 | 20482604 | admission | Continuous variable | mortality | 253 | 13.70% | HR: 1.08 (1.02–1.15), p<0.05 | Moderate | 253 participants, 1999-2008, prospective study |
| Kwakkel | 2010 | 20439108 | 2 days post stroke | Continuous variable | 180-d BI ≥ 19 | 188 | 60.20% | OR: 0.143 (0.069–0.295) | High | 188 participants, prospective study |
| Kwakkel | 2010 | 20439108 | 5 days post stroke | Continuous variable | 180-d BI ≥ 19 | 188 | 60.20% | OR: 0.148 (0.073–0.301) | High | 188 participants, prospective study |
| Kwakkel | 2010 | 20439108 | 9 days post stroke | Continuous variable | 180-d BI ≥ 19 | 188 | 60.20% | OR: 0.144 (0.070–0.297) | High | 188 participants, prospective study |
| Puetz | 2010 | 20389067 | symptom onset | Continuous variable | 90-d mRS 0-2 | 114 | 46% | AUC: 0.8, p<0.001 | High | 114 participants, prospective study, 2002-2007 |
| Hao | 2010 | 20332640 | admission | Continuous variable | Reduced eGFR | 1758 | 26.30% | OR: 1.497 (1.286–1.743) p=0.000 | Moderate | Prospective, 1758 participants, 2002-2008 |
| Knauft | 2010 | 20187750 | admission | Continuous variable | 12-month Modified Barthel Index | 122 | NR | B: -0.256 , p =0.000 | High | 122 participants, retrospective study, 2001-2008 |
| Sanak | 2010 | 20127250 | day 1 of hospitalization | Continuous variable | 90-d mRS 3-6 | 157 | 36.94% | OR: 0.862 (0.705–1.054), p=0.149 | High | Retrospective,157 participants, 2004-2008 |
| Chang | 2010 | 20106589 | admission | Continuous variable | 3-year mortality | 356 | 25.60% | HR: 335.90 (20.72–5446.23)p<0.001 | High | Prospective study, 360 participants, 1998-1999 |
| Johnston | 2009 | 20106589 | admission | Continuous variable | mortality | NA | 25.60% | HR: 2.48 (1.39–4.42) p=0.002 | High | Prospective study, 360 participants, 1998-2000 |
| Nedeltchev | 2010 | 20104376 | admission | Continuous variable | 30-d mortality | 467 | 13% | HR: 1.15 (1.05– 1.25), p = 0.002 | Moderate | 467 participants, prospective study, 2003-2007 |
| Caso | 2010 | 20088729 | admission | Continuous variable | 90-d mRS 3-6 | 1136 | 40.05% | OR: 1.35; (1.30–1.41) | Moderate | 2006-2007,1136 participants, prospective study |
| Kong | 2010 | 20029199 | admission | Continuous variable | 1-year disability | 2774 | 34.50% | OR: 8.31 (5.73–12.06) | Moderate | 2002-2007, prospectie study, 2774 participants |
| Kong | 2010 | 20029199 | admission | Continuous variable | 1-year disability | 2774 | 34.50% | OR: 31.22 (16.16–42.28) | Moderate | 2002-2007, prospectie study, 2774 participants |
| Kong | 2010 | 20029199 | admission | Continuous variable | 1-year disability | 2774 | 28.90% | OR: 4.75 (3.40–6.62) | Moderate | 2002-2007, prospectie study, 2774 participants |
| Kong | 2010 | 20029199 | admission | Continuous variable | 1-year disability | 2774 | 28.90% | OR: 41.73 (16.38–48.00) | Moderate | 2002-2007, prospectie study, 2774 participants |
| koton | 2010 | 20016218 | admission | Continuous variable | 30-d mortality | 1079 | 9.90% | HR: 2.4 (1.1–5.0)p= 0.03 | Moderate | 1079 participants, prospective |
| koton | 2010 | 20016218 | admission | Continuous variable | 30-d mortality | 1079 | 9.90% | HR: 1.5 (0.6–3.7) p=0.4 | Moderate | 1079 participants, prospective |
| koton | 2010 | 20016218 | admission | Continuous variable | 30-d mortality | 1079 | 9.90% | HR: 6.0 (2.5–14.5) p<0.0001 | Moderate | 1079 participants, prospective |
| koton | 2010 | 20016218 | admission | Continuous variable | 30-d mortality | 1079 | 9.90% | HR: 6.1 (2.3–15.8) p=0.0002 | Moderate | 1079 participants, prospective |
| Tei | 2010 | 19943167 | admission | Continuous variable | 90-d mRS 3-6 | 132 | 25.80% | OR: 1.42 (1.16–1.76) p< 0.001 | Moderate | 1994-2008, 132 participants |
| Ovbiagele | 2010 | 19893312 | after 1 week | Continuous variable | 90d-mRS | 581 | 100% | OR: 1.07 (1.04–1.10)p <0.0001 | High | 581 participants, retrospective study |
| Hannon | 2010 | 19893311 | within 72 hours | Continuous variable | 90-d mRS | 568 | NR | B: 0.18, p<0.001 |  | 568 participants, prospective study, 2005-2006 |
| Katan | 2009 | 20035506 | admission | Continuous variable | 90-d mRS, 36 | 362 | 41.70% | OR: 1.17 (1.10–1.23) p<0.0001 | Moderate | Prospective study, 363 participants, 2006-2009 |
| Lee | 2009 | 20005422 | admission | Continuous variable | 180-d mRS 0-1 | 533 | 51.59% | B: 0.362 (1.210–1.705), p<0.01 | Moderate | 533 participants, prospective study, 2004-2006 |
| Nogueira | 2009 | 19875740 | admission | Continuous variable | 90-d mRS ≤2 | 290 | 32.40% | OR: 0.86 (0.81–0.92) p<0.0001 | Moderate | Prospective study, 290 participants, |
| Jovanovic | 2009 | 19647928 | admission | Continuous variable | 90-d mRS 0-1 | 156 | 35.08% | P < 0.01 | High | Prospective study, 2006-2008, 156 participants |
| Saini | 2009 | 19644066 | admission | Continuous variable | 90-d mRS > 2 | 5305 | 58.36% | OR: 1.3 (1.2–1.3) p< 0.001 | Moderate | Prospective study, 5305 participants |
| Hallevi | 2009 | 19628935 | admission | Continuous variable | 90-d mRS 0-2 | 1798 | 25.58% | OR: 6.3 (4.2–9.5), p<0.001 | Moderate | Retrospective study, 1798 participants, 1994-2006 |
| Stroud | 2009 | 19602474 | admission | Continuous variable | 90-d, OHS, 0-1 | 673 | 40.56% | P < 0.01 | Moderate | Retrospective analysis, 673 participants, 2002-2023 |
| Paciaroni | 2009 | 19419717 | admission | Continuous variable | 90-d mortality | 1467 | 9.20% | OR: 1.2, (1.1–1.3) | High | Prospective study, 1467 patients, 2006-2010 |
| Arsava | 2009 | 19380699 | admission | Continuous variable | 180-d mRS 0-6 | 240 | 100% | OR: 1.13 (1.07–1.19 | Moderate | 240 participants, retrospective study, 2003-2006 |
| Johnston | 2009 | 19228853 | Day 5 | Continuous variable | 90-d mRS, 0-1 | 204 | 57% | AUC: 0.87 | High | 204 participants, observational |
| Kissela | 2009 | 19109548 | admission | Continuous variable | 90-d mRS | 451 | 2.6 average | B: 0.03 (0.00–0.05) p=0.016 | High | Retrospective, 451 patients, 1993 |
| Khartionova | 2009 | 19018138 | admission | Continuous variable | admission Hyperdense middle cerebral artery sign(HMCA) | 10023 | 19% | B: 0.119, p<0.001 | High | 1023 participants, prospective, 2002-2006 |
| Stead | 2009 | 18357419 | admission | Continuous variable | 90-d mortality | 447 | 17.67% | P < 0.01 | High | 447 participants, 2001-2004 |
| Tartaglia | 2008 | 19235441 | pre-treatment | Continuous variable | 90-d mRS 3-6 | 130 | 58.50% | p<0.001 | High | 130 participants, retrospective study, 1998-2005 |
| Millan | 2008 | 19049558 | admission | Continuous variable | 90-d mRS 3-6 | 254 | 44.10% | OR: 1.21 (1.12 to 1.31) p<0.001 | Moderate | 254 participants, retrospective study |
| Hong | 2008 | 19049549 | admission | Continuous variable | 90-d mRS, 3-6 | 1254 | 34.90% | OR: 2.96 (1.62–5.40) | Moderate | 1262 participants, prospective study, 2004-2005 |
| Hong | 2008 | 19049549 | admission | Continuous variable | 90-d mRS, 3-6 | 1254 | 34.90% | OR: 19.48 (10.18–37.27) | Moderate | 1263 participants, prospective study, 2004-2005 |
| Wahlgren | 2008 | 18927461 | admission | Continuous variable | 90-d mRS 0-2 | 6947 | 50% | OR: 0.34 (0.32-0.37) | Moderate | 6947 participants, review of RCT, 2002-2006 |
| Yong | 2008 | 18703813 | randomization | Continuous variable | 90-d mortality | 748 | 9.80% | P < 0.01 | Moderate | 756 participants, patient sample from randomized controlled trial |
| Ovbiagele | 2008 | 18583560 | admission | Continuous variable | 90-d mRS ≥ 2 | 659 | 16.55% | P < 0.01 | High | 659 participants, prospective study of RCT |
| Uyttenboogaart | 2008 | 18338194 | admission | Continuous variable | 90-d mRS≤ 2 | 252 | 49% | OR: 0.84 (0.78–0.91) p< 0.001 | Moderate | 252 participants, prospective study, 2002-2006 |
| Uyttenboogaart | 2008 | 18338194 | admission | Continuous variable | sICH | 252 | 5.20% | OR: 1.23 (1.02–1.48) p=0.03 | Moderate | 256 participants, prospective study, 2002-2006 |
| Jeng | 2008 | 18299138 | admission | Continuous variable | 90-d mortality or institutional care | 850 | 38% | OR: 1.10 (1.06–1.14), p<0.005 | Moderate | 2002-2006, 850 participants, prospective study |
| Jeng | 2008 | 18299138 | admission | Continuous variable | 90-d mortality or institutional care | 850 | 38% | OR: 1.19 (1.12–1.26), p<0.005 | Moderate | 2002-2006, 850 participants, prospective study |
| Jeng | 2008 | 18299138 | admission | Continuous variable | 90-d mortality | 850 | 17% | OR: 1.07 (1.04–1.10), p<0.005 | Moderate | 2002-2006, 850 participants, prospective study |
| Roquer | 2007 | 18004645 | admission | Continuous variable | in-hospital mortality | 1527 | 12.90% | HR: 1.142 (1.113–1.172), p=0.0001 | Moderate | 1997-2005, 1527 participants, prospective study |
| Liu | 2007 | 17600616 | admission | Continuous variable | 2-y mRS, 3-6 | 489 | 43.80% | OR: 2.619 (1.584 - 4.330), p=0.001 | High | Retrospective, 489 patients, 2002-2008 |
| Ois | 2007 | 17525388 | admission | Continuous variable | 90-d mortality | 1220 | 15.70% | OR: 1.17 (1.14–1.21), p<0.05 | Moderate | 1224 participants, prospective study, 2003 to 2006 |
| Roquer | 2007 | 17502469 | admission | Continuous variable | 30-d mortality | 1527 | 13.80% | OR: 1.22 (1.18-1.25) p<.001 | Moderate | 1528 participants, 1997-2005, retrospective study |
| Elkind | 2007 | 17353472 | admission | Continuous variable | 90-d mRS ≤ 1 | 333 | 27.60% | OR: 0.23 (0.13–0.41) | Moderate | 334 participants, review of randomized controlled trial |
| Elkind | 2007 | 17353472 | admission | Continuous variable | BI ≥ 95 | 333 | 39.60% | OR: 0.31 (0.18–0.53) | Moderate | 337 participants, review of randomized controlled trial |
| Elkind | 2007 | 17353472 | admission | Continuous variable | 90-d mortality (Alive) | 333 | 80.91% | OR: 0.39 (0.17–0.89) | Moderate | 341 participants, review of randomized controlled trial |
| Montaner | 2006 | 1660121 | admission | Continuous variable | 90-d mortality | 143 | 13.29% | OR: 3.01 (0.95 to 9.54) P=0.061 | High | 145 participants, prospective study |
| Wu | 2017 | 28579505 | admission | Continuous variable | 90-d mRS dichotomized 0-2 vs 3-6 | 383 | Favorable outcome: 253 (66%), unfavorable 130 (34%) | AOR 1.162, 95% CI 1.103-1.223 (p<0.001) | Moderate | Thrombolysis only included; no loss to follow-up description; unknown missing data and no imputation; presumed high risk of bias due to no mention of WLST. |
| Munsch | 2016 | 26585396 | admission | Continuous variable | mRS ≤1 3 months | 428 | 43.2% | Beta −0.320 , 95% CI −0.443 to −0.196 | Moderate | Selected patients, limited data imputation, no WLST mention. |
| Munsch | 2016 | 26585396 | admission | Continuous variable | MoCA at 3 months | 428 | MoCA>25 in 46.4%) | Beta −0.158 , 95% CI −0.264 to −0.051 | Moderate | Selective patients, missing data not imputed, no WLST mention. |
| Shi | 2016 | 26409718 | admission | Continuous variable | death/dependency = mRS 3-6 @ 12 months | 2168 | NA | AOR 23.06 (95% CI 14.24-37.34) | Moderate | Chinese hospital registry, AIS patients within 14 days, 12-month follow-up; no loss/imputation data, adequate analysis, no WLST/demise details. |
| Dogan | 2016 | 25228671 | admission | Continuous variable | in-hospital mortality | 692 | 35.7% | AOR 2.085 95% CI [1.835-2.370] | High | Elderly patient’s only, incomplete data excluded, no data comparison, retrospective design, no WLST info. |
| Haeusler | 2015 | 26349854 | admission | Continuous variable | Long-term mortality at 8-years | 1013 (only 892 had death data at 8 years) | 42% | AOR 1.08, 95% CI 1.06-1.1), p<0.001 | High | Berlin retrospective study, excluded patients without survival status, no data comparisons or death details. |
| Ryu | 2017 | 28008000 | admission | Continuous variable | 90-d mRS | 5035 | 100% | OR 1.22 (1.21-1.24); p<0.001 | Moderate | 5035 patients from 8005 initially evaluated from 2011-2012; many post-hoc analyses conducted |
| Kim | 2018 | 30355207 | admission | Continuous variable | 90-d mRS 0-2 | 309 | 49.2% | P < 0.01 | Moderate | 308 patients from three interventional studies using the Solitaire stent retriever; LVO only, undergoing endovascular therapy; prospectively collected data, retrospectively analyzed |
| Demeestere | 2018 | 30355098 | admission | Continuous variable | 90-d mRS 0-2 | 156 | 58.9% | OR 0.89 (0.82-0.96); p<0.01 | High | 156 patients, retrospectively analyzed, but prospectively enrolled in the CRISP study evaluating LVO in the anterior circulation who underwent endovascular therapy and had successful reperfusion |
| Sico | 2018 | 30294472 | admission | Continuous variable | mortality, in-hospital | 3750 | 3.6% | OR 1.13 (1.11 to 1.15) | Moderate | 3750 patients from 131 Veterans Health Admin facilities with stroke in 2007; retrospectively collected and analyzed data |
| Sico | 2018 | 30294472 | admission | Continuous variable | 30-d mortality | 3750 | 7.8% | OR 1.145 (1.12 to 1.16) | Moderate | 3750 patients from 131 Veterans Health Admin facilities with stroke in 2007; retrospectively collected and analyzed data |
| Sico | 2018 | 30294472 | admission | Continuous variable | 180-d mortality | 3750 | 14.5% | OR 1.12 (1.11 to 1.14) | Moderate | 3750 patients from 131 Veterans Health Admin facilities with stroke in 2007; retrospectively collected and analyzed data |
| Sico | 2018 | 30294472 | admission | Continuous variable | 1-y mortality | 3750 | 18.8% | OR 1.11 (1.09 to 1.13) | Moderate | 3750 patients from 131 Veterans Health Admin facilities with stroke in 2007; retrospectively collected and analyzed data |
| Chen | 2018 | 30166435 | admission | Continuous variable | 90-d mRS 0-2 | 104 | 42.3% | OR 0.912 (0.826-1.006); p=0.66 | High | 104 consecutive patients with M1 occlusion ischemic stroke who could be evaluated for thrombus permeability based on conventional CTA |
| Maus | 2018 | 29946292 | admission | Continuous variable | 90-d mRS 3-6 | 392 | 60.2% | OR 2.6 (2-3.3) | Moderate | Retrospective analysis of 392 patients with LVO undergoing thrombectomy between 2014-2017 |
| Bentes | 2018 | 29935475 | admission | Continuous variable | mRS >2, at discharge | 151 | 65.5% | p<0.01 | Moderate | 151 prospectively identified consecutive patients with anterior circulation ischemic stroke; followed for 12 months; primary study was regarding EEG findings and association with outcomes |
| Bentes | 2018 | 29935475 | admission | Continuous variable | 1-y mRS >2 | 151 | 50.9% | p<0.01 | Moderate | 151 prospectively identified consecutive patients with anterior circulation ischemic stroke; followed for 12 months; primary study was regarding EEG findings and association with outcomes |
| Laredo | 2018 | 29934530 | admission | Continuous variable | 90-d mRS >2 | 195 | NR | p<0.01 | Moderate | 195 patients from two cohorts, primarily evaluating the association of insular involvement with outcomes; reporting of outcomes was sparce |
| Laredo | 2018 | 29934530 | admission | Continuous variable | 90-d mortality | 195 | NR | p<0.01 | Moderate | 195 patients from two cohorts, primarily evaluating the association of insular involvement with outcomes; reporting of outcomes was sparce |
| Nagel | 2018 | 29777016 | admission | Continuous variable | 90-d mRS 2-6 | 1480 | 52.9% | p<0.01 | Moderate | 1480 patients from the alteplase arm of the ENCHANTED trial |
| Nagel | 2018 | 29777016 | admission | Continuous variable | 90-d mRS 3-6 | 1480 | 37.8% | p<0.01 | Moderate | 1480 patients from the alteplase arm of the ENCHANTED trial |
| Nagel | 2018 | 29777016 | admission | Continuous variable | 90-d mortality | 1480 | 9.8% | p<0.01 | Moderate | 1480 patients from the alteplase arm of the ENCHANTED trial |
| Dargazanli | 2018 | 29626134 | admission | Continuous variable | 90-d mRS <3 | 290 | 50.3% | p<0.01 | Moderate | 290 patients with successful reperfusion (TICI 2b, 2C, 3 scores) from the ASTER study; anterior circulation stroke, undergoing mechanical thrombectomy |
| Dargazanli | 2018 | 29626134 | admission | Continuous variable | 90-d mortality | 290 | 14.8% | p<0.01 | Moderate | 290 patients with successful reperfusion (TICI 2b, 2C, 3 scores) from the ASTER study; anterior circulation stroke, undergoing mechanical thrombectomy |
| Al-Ajlan | 2018 | 29170266 | day 1 of hospitalization | Continuous variable | 90-d mRS 0-2 | 206 | 35.9% | p<0.01 | Moderate | 206 prospectively enrolled patients in the REVASCAT trial; 103 received mechanical thrombectomy for anterior circulation, LVO |
| Park | 2018 | 28624400 | admission | Continuous variable | 3-y mortality | 2069 | 18% | NR | Moderate | 2069 consecutive patients prospectively evaluated from 2002-2010 for LV mass and geometry |
| Bentes | 2017 | 29588974 | admission | Continuous variable | mRS >2, discharge | 151 | 65.6% | OR = 1.18 (1.10–1.28); p < 0.001 | Moderate | 151 prospectively identified consecutive patients with anterior circulation ischemic stroke; followed for 12 months; primary study was regarding EEG findings and association with outcomes |
| Bentes | 2017 | 29588974 | admission | Continuous variable | 1-y mRS >2 | 151 | 50.9% | OR = 1.18 (1.1–1.28); p < 0.001 | Moderate | 151 prospectively identified consecutive patients with anterior circulation ischemic stroke; followed for 12 months; primary study was regarding EEG findings and association with outcomes |
| Bentes | 2017 | 29588974 | admission | Continuous variable | mortality, discharge | 151 | 4.6% | p = 0.032 | Moderate | 151 prospectively identified consecutive patients with anterior circulation ischemic stroke; followed for 12 months; primary study was regarding EEG findings and association with outcomes |
| Bentes | 2017 | 29588974 | admission | Continuous variable | 1-y mortality | 151 | 15.2% | OR = 1.18 (0.7–1.3); p = 0.001 | Moderate | 151 prospectively identified consecutive patients with anterior circulation ischemic stroke; followed for 12 months; primary study was regarding EEG findings and association with outcomes |
| Liu | 2017 | 29201238 | admission | Continuous variable | 90-d mRS >2 | 116 | 34.5% | OR 1.278 (1.145-1.426); p<0.001 | High | Retrospective study of 116 patients, who all received a follow up MRI and none received thrombolysis for initial stroke treatment |
| Bhaskar | 2017 | 29133696 | admission | Continuous variable | 90-d mortality | 608 | 20.7% | OR 1.16 (1.12-1.2); P<0.0001 | Moderate | Retrospective study of 608 patients from 2006-2013; registry-based study; including only patients with complete data |
| Bhaskar | 2017 | 29133696 | admission | Continuous variable | 90-d mRS >2 | 608 | 53.7% | OR 1.16 (1.13-1.2); P<0.0001 | Moderate | Retrospective study of 608 patients from 2006-2013; registry-based study; including only patients with complete data |
| Xue | 2017 | 28978089 | admission | Continuous variable | 90-d mRS >2 | 438 | 65.7% | OR 1.13 (1.08-1.22); p<0.001 | Moderate | 438 patients prospectively enrolled from 2015-2016 |
| Ahn | 2017 | 28877565 | admission | Continuous variable | mortality, 33 months | 1692 | 19.6% | NR | Moderate | 1692 patients retrospectively included from a single hospital from 2007-2011; all with ECG findings and troponin |
| Kim | 2017 | 28176499 | admission | Continuous variable | 90-d mRS <3 | 104 | 44% | OR 3.03 (1.19–7.73); p=0.02 | Moderate | 104 patients from a prospective registry of 5558 patients, with endovascular recanalization treatment for LVO |
| Kim | 2017 | 28176499 | admission | Continuous variable | 90-d mortality | 104 | 15% | OR 0.25 (0.07–0.98); p=0.046 | Moderate | 104 patients from a prospective registry of 5558 patients, with endovascular recanalization treatment for LVO |
| Zhang | 2014 | 24994827 | admission | Continuous variable | 90-d mRS >2 | 129 | 57% | OR 1.08 (1.00–1.16); p=0.041 | Moderate | 129 consecutive, prospectively enrolled patients with stroke undergoing endovascular therapy at a single hospital from 2006-2013 |
| Shi | 2014 | 24876082 | admission | Continuous variable | 90-d mRS >2 | 228 | 53.5% | OR 1.08 (1.02–1.15); p=0.0055 | Moderate | 2228 patients with successful LVO recanalization from the MERCI, TREVO, and TREVO2 trials |
| Ricciardi | 2014 | 24851928 | admission | Continuous variable | 90-d mRS <3 | 159 | 46.5% | OR 0.84 (0.76-0.93; p=0.001 | Moderate | 159 patients in a prospective registry at a single hospital with LVO, presenting within 12 hours of onset and evaluated using the IPAQ assessing pre-stroke physical activity |
| Cao | 2014 | 24742803 | admission | Continuous variable | 90-d mRS >2 | 55 | 58.2% | Beta 0.207, Standard error 0.091, p=0.023 | High | 217 consecutive stroke patients with alteplase or endovascular therapy, retrospectively evaluated from 2009-2011 |
| Cao | 2014 | 24742803 | admission | Continuous variable | 90-d mortality | 55 | 21.8% | Beta 0.239, Standard error 0.116, p=0.028 | High | 217 consecutive stroke patients with alteplase or endovascular therapy, retrospectively evaluated from 2009-2011 |
| Kim | 2014 | 24719133 | 7-day | Continuous variable | 30-d mortality | 2820 | 2.8% | OR 3.185 (1.522-6.664); p=0.002 | Moderate | 2820 patients, 344 with early neurologic deterioration, which was the primary analysis; 200-2008 single hospital, excluding patients with recurrent stroke, alteplase receipt, and late >3 day presentation |
| Kim | 2014 | 24719133 | admission | Continuous variable | 30-d mortality | 2820 | 2.8% | P < 0.01 | Moderate | 2820 patients, 344 with early neurologic deterioration, which was the primary analysis; 200-2008 single hospital, excluding patients with recurrent stroke, alteplase receipt, and late >3 day presentation |
| Kim | 2014 | 24719133 | 7-day | Continuous variable | 3-y mortality | 2820 | 17.7% | HR 1.571 (1.165-2.119); p=0.003 | Moderate | 2820 patients, 344 with early neurologic deterioration, which was the primary analysis; 200-2008 single hospital, excluding patients with recurrent stroke, alteplase receipt, and late >3 day presentation |
| Kim | 2014 | 24719133 | admission | Continuous variable | 3-y mortality | 2820 | 17.7% | P= 0.001 | Moderate | 2820 patients, 344 with early neurologic deterioration, which was the primary analysis; 200-2008 single hospital, excluding patients with recurrent stroke, alteplase receipt, and late >3 day presentation |
| Inoa | 2014 | 24686370 | admission | Continuous variable | 90-d modified Barthel Index (mBI) <19 | 1569 | 58% | AC: OR 0.86 (0.84–0.89); p<0.001 \| PC: OR 0.88 (0.83–0.94); p<0.001 | Moderate | 1569 patients from a prospective registry, retrospectively analyzed; primary analysis was between anterior and posterior circulation stroke and associated outcomes/prognosis |
| Hao | 2014 | 24685995 | admission | Continuous variable | 90-d mRS >2 | 215 | 36.3% | RR 1.27 (1.17-1.38); p<0.001 | Moderate | 215 patients from a prospective registry from 2009-2011 with severe intracranial stenosis or occlusion; included within 30 days of symptom onset; primary evaluation was on high blood pressure upadmission association with outcomes |
| Hoshino | 2013 | 23714421 | admission | Continuous variable | 90-d mRS 0-2 | 148 | 56.1% | OR 1.37 (1.22–1.55); p<0.001 | High | 148 stroke patients with a history of CAD, evaluating the association of CHADS2 score on functional outcome |
| Boers | 2019 | 29627794 | admission | Continuous variable | 90-d mRS 0-2 | 651 | 39.50% | cOR= 0.82 per 5 points (95% CI, 0.74-0.90; *P* = .001) | Low | Well-designed study |
| Ho | 2016 |  | admission | Continuous variable | In-hospital mortality | 611 | 15.90% | OR 1.08; 95 % CI 1.06–1.11; P < 0.01 | Low | Well-designed study |
| Ong | NA | 28934363 | admission | Continuous variable | In-hospital mortality |  | n=80; 3.1% - number with NIHSS 16-25 = 24 (8%); >25 = 41 (28%) | p <0.001 for NIHSS >15; NIHSS 16-26 HR for death 22.33 (9.03-55.20); NIHSS >25 OR = 91.00 (37.76 - 219.30) | Low | One-hospital based study with potential limited generalizability to urban areas. Missing information on pre-stroke conditions and timing of stroke onset to hospital admission. Approximately 25% loss to follow-up at one year, but no gender-based differences in follow-up rate. |
| Corso | NA | 27437502 | admission | Continuous variable | Long-term mortality (mean 3.3; over up to 8 years) |  | 16.70% | HR 3.54 (2.87–4.36), p <0.0001 | Low | The predominantly Caucasian population may limit generalizability to other ethnic groups. Use of prestroke mRS score is not standardized. The study did not investigate the impact of hospital treatment practices. |
| Rangaraju | NA | 27586683 | 24 -48 hours | Continuous variable | 30-d poor outcome = mRS 4-6 |  | 65.40% | HR 1.28 (1.21 - 1.35), p < 0.001 | Low | Lack of information on early withdrawal of care and its potential influence on outcome assessment. Use of a 1-month instead of a 3-month follow-up for the primary outcome measure (mRS) may underestimate long-term recovery. Exclusion of patients without a 24-48 hour NIHSS and limited data on post-discharge variables and outcomes beyond 1 month. Lack of inclusion of patients over the age of 85 and no assessment of the relationship between 24-48 hour NIHSS and long-term quality of life. |
| Haussen | 2016 | 27507858 | admission | Continuous variable | 90-d good outcome (mRS 0-2) |  | 54.00% | OR, 0.87 (0.83–0.92) P<0.01 | Low | Retrospective nature, potential bias towards CT perfusion selection and patients undergoing mechanical thrombectomy. The study's focus on patients with confirmed large vessel occlusion may introduce a selection bias towards those with larger ischemic cores. The absence of predictor and correlation analyses for poor outcomes and interobserver coefficients for NCCT ASPECTS readers further restricts the study's comprehensive evaluation. |
| Yao | 2016 | 23599933 | admission | Continuous variable | 180-d mRS >2 vs 2 or less |  | 9.80% | OR 2.90(2.52–3.33), p < 0.001 | Low | Study participation- moderate ROB, single center, retrospective, no info on race/ethnicity (though Singapore); study attrition- no info on loss to f/u; prognostic factor measurement- moderate ROB, study confounding- moderate ROB, |
| Kim | 2016 |  | admission | Continuous variable | 90-d mRS 3-6 (="poor outcome") |  | not reported | OR 1.30 (1.26-1.34) | Low | All low |
| Chang | 2016 | 26835227 | admission | Continuous variable | 180-d functional independence (per FIM) |  | 38% | 0.918 (0.889-0.948) p <0.001 | Moderate | Prospective study, 360 participants, 1998-1999  Study participation- high ROB; study attrition- moderate ROB; prognostic factor measurement- moderate ROB; outcome measurement- moderate ROB; study confounding- moderate ROB; statistical analysis and reporting- moderate ROB; |
| Chang | 2016 | 26835227 | admission | Continuous variable | Satisfaction' (per Euro Quality of life 5D) |  | 52% | 0.896 (0.8580.936), p <0.001 | Moderate | Prospective study, 360 participants, 1998-1999  Study participation- high ROB; study attrition- moderate ROB; prognostic factor measurement- moderate ROB; outcome measurement- moderate ROB; study confounding- moderate ROB; statistical analysis and reporting- moderate ROB; |
| Todo | 2016 | 26935116 | admission | Continuous variable | 90-d mRS 0-2 | 56 | 44% | 0.573 (0.277 - 1.185), p =0.13 | Moderate | Study participation- moderate ROB, single center, retrospective; study attrition- moderate ROB; outcome measurement- moderate ROB; statistical analysis and reporting- moderate ROB; self fulfilling prophecy- moderate ROB |
| Yeo | 2016 | 23599933 | admission | Continuous variable | poor outcome = mRS 2-6 | 98 | 46% | OR 1.17 per point (1.08 - 1.26, p < 0.001) | Low | Study participation- moderate ROB, single center, retrospective, no info on race/ethnicity (though Singapore); study attrition- no info on loss to f/u; prognostic factor measurement- moderate ROB, study confounding- moderate ROB, |
| Yeo | 2016 | 23599933 | admission | Continuous variable | 90-d mortality | 16 | 7.60% | OR 1.14 per point (1.06 - 1.235, p =0.001) | Low | Study participation- moderate ROB, single center, retrospective, no info on race/ethnicity (though Singapore); study attrition- no info on loss to f/u; prognostic factor measurement- moderate ROB, study confounding- moderate ROB, |
| Tziomalos | 2016 | 25512800 | admission | Continuous variable | mRS 2-5 at discharge | 322 | 58.30% | RR 1.64 (1.44-1.86, p <0.001) | Low | Lack of available magnetic resonance imaging and arterial imaging, hindering determination of stroke subtypes and assessment of stroke location's impact on functional outcome. |
| Tziomalos | 2016 | 25512800 | admission | Continuous variable | mortality at discharge | 56 | 9.20% | RR 1.18 (1.13-1.23, p < 0.001) | Low | Lack of available magnetic resonance imaging and arterial imaging, hindering determination of stroke subtypes and assessment of stroke location's impact on functional outcome. |
| de Rueda | 2015 | 26219650 | admission | Continuous variable |  | 85 | 57% | OR 1.2 (1.1-1.3, p = 0.001) | Low | Moderate study attrition and self-fulfilling prophecy. Small sample size, residual confounding, reader bias, limited collateral assessment, imperfect correlation with MRI concepts, and absence of clot measurements. |
| Skagen | 2015 | 25922156 | admission | Continuous variable | 90-d mRS 0-2 | 28 | 60.90% | OR 1.2 (1.0-1.4) p=0.014 | Low | Study attrition- moderate; prognostic factor measurement- moderate; outcome measurement- moderate; self fulfilling prophecy- moderate |
| Skagen | 2015 | 25922156 | admission | Continuous variable | 90-d mRS 0-2 | 40 | 37.70% | OR 1.3 (1.2-1.45) p = 0.001 | Low | Study attrition- moderate; prognostic factor measurement- moderate; outcome measurement- moderate; self fulfilling prophecy- moderate |
| Lee | 2017 | 29067622 | admission | Continuous variable | 180-d mortality | unknown | unknown | OR 1.135 (1.078-1.195) | High | Prognostic factor measurement- high ROB, without knowing the prevalence of the outcome, it's hard to evaluate prognostic factors; outcome measurement- high ROB; study confounding- moderate ROB; statistical analysis and reporting- high ROB; self fulfilling prophecy- high |
| Lee | 2017 | 29067622 | admission | Continuous variable | 180-d Good functional outcome = mRS 2 or less | unknown | unknown | OR 0.791 (0.742 - 0.844) | High | Prognostic factor measurement- high ROB, without knowing the prevalence of the outcome, it's hard to evaluate prognostic factors; outcome measurement- high ROB; study confounding- moderate ROB; statistical analysis and reporting- high ROB; self fulfilling prophecy- high |
| Menon | 2015 | 25791716 | admission | Continuous variable | 90-d mRS 0-2 | unknown | unknown | p <0.06 | High | Study participation- high ROB, subgroup of RCT, not well described; study attrition- moderate, subgroup of RCT. RCT is well-known and assumptions can be made that f/u was good; prognostic factor measurement- moderate, not enough information provided for 'collateral score'; outcome measurement- moderate ROB; study confounding- moderate ROB; self fulfilling prophecy- moderate |
| Rusanen | 2015 | 26352696 | admission | Continuous variable | 90-d mRS 0-2 | unknown | unknown | OR 0.78 per score (0.69–0.92), p = 0.002 | High | Study attrition- moderate ROB, no info on loss to f/u; prognostic factor measurement- moderate ROB, unknown prevalence of outcome; outcome measurement- moderate ROB; study confounding- moderate ROB; statistical analysis and reporting- moderate ROB; self fulfilling prophecy- moderate ROB, unknown prevalence of mRS including death, no mention of WDLST |
| Chen | 2018 | 30166435 | admission | Continuous variable | 90-d Lawton ADL scale | not provided | not provided | Beta 0·40 (0·15–0·64), p = 0.002 | High | Study participation- moderate ROB; study attrition- moderate ROB; prognostic factor measurement- moderate ROB; outcome measurement- moderate rob; statistical analysis and reporting- moderate ROB; self-fulfilling prophecy- moderate ROB |
| Chen | 2018 | 30166435 | admission | Continuous variable | 90-d SSQOL | not provided | not provided | Beta -0.60 (-1.57-0.37) p = 0.22 | High | Study participation- moderate ROB; study attrition- moderate ROB; prognostic factor measurement- moderate ROB; outcome measurement- moderate rob; statistical analysis and reporting- moderate ROB; self-fulfilling prophecy- moderate ROB |
| Sillanppa | 2015 | 22566978 | admission | Continuous variable | 90-d mRS 0-2 | 54 | 52% | OR 0.82 (0.73-0.93) p=0.001 | Moderate | Statistical analysis and measurement- moderate ROB; self-fulfilling prophecy- moderate ROB |
| Wolfahrt | 2015 | 25866318 | admission | Continuous variable | overall mortality | 95 | 18% | By quartile (1st = reference): 2nd = HR 1.15 (0.28-4.66, p=0.85); 3rd HR 3.5 (1.03-11.90, p = 0.045); 4th HR 13.49 (4.06 - 44.78, p <0.001) | Moderate | The study had high risk of bias (ROB) in terms of attrition, with a wide range of follow-up and no information on loss to follow-up. Outcome measurement had a moderate ROB, as mortality was assessed from a registry. Statistical analysis and reporting also had moderate ROB. Additionally, there was a moderate risk of a self-fulfilling prophecy effect. |
| Yoo | 2014 | 24503670 | admission | Continuous variable | 90-d mRS 0-2 |  | 36.90% | OR 0.93; *P*=0.01 | Low | No control group without intra-arterial treatment (IAT) limits definitive conclusions on treatment benefits and risks |
| Perez | 2011 | 20954836 | admission | Continuous variable | poor outcome (mRS 3-6) | NA | 75% | OR 1.41 (1.22-1.62) | Low | 204 participants, prospective study |
| Perez | 2011 | 20954836 | admission | Continuous variable | in-hospital mortality | 516 | 7.20% | OR 1·11 (95% CI 1·10–1·13, *P* < 0·001) | Low | 204 participants, prospective study |
| Ducci | 2018 | 29263821 | after 90-d | Continuous variable | mRS (functional outcome 3-5) | 144 | 47.20% | OR = 1.3, 95%CI = 1.16–1.45, p < 0.001)  Sensitivity 73.5%  Specificity 78.9% | High | Study participation- moderate ROB; study attrition- high ROB, 15% attrition due to death and few unclear; prognostic factor measurement- moderate ROB; outcome measurement- high ROB, telephone interview so high ROB for outcome measurement; study confounding- moderate ROB, small sample size although key variables are included; self-fulfilling prophecy- moderate ROB |
| Nedeltchev | 2004 | 15654030 | admission | Continuous variable | 90-d mRS 0-2 | 203 | NA | p=0.011 | High | Study confounding- high ROB; statistical analysis and reporting- high; self-fulfilling prophecy- moderate ROB |
| Cucchiara | 2004 | 14707586 | admission | Continuous variable | 90-d mRS 0-2 | 564 | NA | p<0.001 | Low | Well-designed study |
| Demchuk | 2001 | 11502916 | prior to tPA treatment | Continuous variable | 90-d mRS 0-2 | 616 | NA | In a multivariate analysis adjusted for days to final assessment and pre-treatment NIHSS, the following odds ratios (OR) were found: NIHSS 1-5: OR 1.00 (reference) NIHSS 6-10: OR 0.97, 95% CI 0.51-1.87, p=0.934 NIHSS 11-15: OR 0.40, 95% CI 0.20-0.79, p=0.008 NIHSS 16-20: OR 0.27, 95% CI 0.13-0.56, p<0.001 NIHSS >20: OR 0.05, 95% CI 0.02-0.16, p<0.001 | High | The study had high bias due to many excluded patients (539/1205) with missing information, primarily serum blood glucose (n=421). Comparing the entire cohort with included patients showed significant differences in race, time-to-treatment, and CT scan results. Outcome measurement had high bias as NIHSS and mRS categories were estimated from medical records, raising concerns about the reliability and validity of evaluating them solely through records. |
| M.R. Frankel | 2000 | 11061250 | admission | Continuous variable | 90-d poor outcome = mRS > 3 | n=310 | NIHSS >17: 86% poor  NIHSS <17, 32% poor | P<0.001 | Low | Well-designed study |
| Adams | 1999 | 10408548 | admission | Continuous variable | 7-d excellent outcome | 1268 | NA | OR 0.76, 95% CI (0.72-0.80) | Low | Well-designed study |
| Adams | 1999 | 10408548 | admission | Continuous variable | 90-d dementia | 337 | 31.8% | p=0.3998 | Low | Well-designed study |
| Pohjasvaara | 1998 | 9445332 | admission | Continuous variable | 90-d dementia | 146 | 24.6% | p<0.001 | Low | Well-designed study |
| Censori | 1996 | 8685929 | after 90-d | Continuous variable | 90-d dementia | NA | NA | p<0.001 | Moderate | The study included only 15 dementia patients, with 10 of them also having aphasia, and had moderate risks of bias due to attrition and confounding. Furthermore, the presence of older patients who died within 3 months after a stroke may have influenced the dementia rate, potentially introducing a self-fulfilling prophecy bias. |
| Skafida | 2018 | 31008348 | admission | Continuous variable | 90-d mortality | 1271 | NA | p=0.001 | Low | All low |
| Wouters | 2018 | 29867722 | admission | Continuous variable | 90-d mRS 0-2 | 369 | Good functional outcome present in 279/369 (76%) | AOR 0.81, 95% CI 0.76-0.96 AUC = 0.82 | Moderate | Study attrition, with a moderate risk of bias, involved 122 patients with missing data. It is possible that these patients were in a more severe condition. |
| Wouters | 2018 | 29867722 | day 1 after hospitalization | Continuous variable | 90-d mRS 0-2 | 369 | Good functional outcome present in 279/369 (76%) | AOR 1.34, 95% CI 1.21-1.49 AUC = 0.86 | Moderate | Study attrition, with a moderate risk of bias, involved 122 patients with missing data. It is possible that these patients were in a more severe condition. |
| Toon | 2017 | 28178407 | admission | Continuous variable | 90-d mRS 0-2 | 335 | good outcome achieved in 151/335 (45.1%) of patients | AOR 0.908, 95% CI 0.855-0.965 p=0.002 | Low | Well-designed study |
| Su | 2016 | 27076999 | admission | Continuous variable | mRS at discharge mRS > 2 = poor outcome | 871 | Poor outcome in 509/871 (58%) | AOR 19.52, 9.59-39.73), p<0.001 | Moderate | The study had a moderate risk of bias regarding confounding. |
| Su | 2016 | 27076999 | admission | Continuous variable | Mortality | 871 | Mortality in 31/871 (3.6%) | AOR 8.08, 2.36-13.27, p<0.001 | Moderate | The study had a moderate risk of bias regarding confounding. |
| Mansour | 2015 | NA | day 1 of hospitalization | Continuous variable | mortality | 127 | NA | OR 1.15, 95% CI 1.07-1.24 p<0.001 | Low | Well-designed study |
| Mansour | 2015 | NA | day 3 of hospitalization | Continuous variable | mortality | 127 | NA | OR 1.73, 95% CI 1.35-2.21 p<0.001 | Low | Well-designed study |
| Mansour | 2015 | NA | day 1 of hospitalization | Continuous variable | unfavorable outcome mRS score 3-6 | 127 | NA | OR 1.28, 95% CI 1.18-1.39 p<0.001 | Low | Well-designed study |
| Mansour | 2015 | NA | day 3 of hospitalization | Continuous variable | unfavorable outcome mRS score 3-6 | 127 | NA | OR 1.36, 95% CI 1.22-1.51 p<0.001 | Low | Well-designed study |
| Park | 2020 | 32323503 | admission | Continuous variable | 90-d mRS 0-2 | 136 | Good outcome, n=35 (25.7%) poor outcome, n=101 (74.3%) | OR 0.760, 95% CI 0.638-0.905 p=0.002 | Low | Recanalization status was assessed by two blinded investigators. The interrater agreement, measured by kappa values, was high at 0.875, 0.813, and 0.905. |
| Pikija | 2018 | 30353493 | admission | Continuous variable | 90-d mRS 0-2 | 174 | n=83 (50%) | OR 0.941, 95% CI 0.855-1.035 p=0.211 | Low | The study had limitations, including its retrospective design, LDL-C was major predictor evaluated. The demographics, stroke severity, and outcomes were comparable to large EVT trials, with similar rates of favorable outcomes and mortality. |
| Potreck | 2019 | 30887195 | admission | Continuous variable | 90-d mRS 0-2 | 131 | NA | OR 0.90, 0.82-1.00 p=0.045 | Low | Study attrition- retrospective study, n=156. 25 excluded due to image quality. These patients could have had more severe/complicated cases or just due to other reasons. ; outcome measurement- image quality was evaluated blindly, excluding 25 patients |
| Henden | 2018 | 30117146 | admission | Continuous variable | 90-d mRS 0-2 | 198 | NA | OR 1.092, 95% CI 0.997-1.197 p=0.059 | Low | Well-designed study |
| Madelung | 2017 | 28905995 | admission | Continuous variable | 90-d mRS 0-2 | 187 | NA | OR 0.93, 0.87-0.99 p=0.028 | Moderate | The study did not specify the protocol for evaluating the modified Rankin Scale (mRS) outcome. The outcome measurement had a high risk of bias. There was a moderate risk of bias related to self-fulfilling prophecy, possibly due to a treatment suspension policy where 35 participants did not receive treatment, potentially indicating severe conditions. |
| Broocks | 2019 | 31667625 | admission | Continuous variable | 90-d mRS 0-2 | 178 | n=69 (39%) 90-d mRS 0-2  n=109 (61%) 90-d mRS 3-6 | OR 0.87, 0.78-0.95 p=0.009 | High | Study participation had a moderate risk of bias, while outcome measurement also had a moderate risk, lacking sufficient information. The study did not specify the registry used for functional outcome assessment or the university involved. The statistical analysis and reporting also had a moderate risk of bias, as the data were not explicitly provided. |
| Ducci | 2017 | 29125072 |  | Continuous variable | 90-d dependence (early) mRS between 3-5 | 144 | Dependency in n=68 (47.2%) | Sensitivity 73.5%, specificity 78.9% p<0.001 | Moderate | The study had moderate risk of bias in participation and high risk of bias in attrition, with approximately 15% attrition due to death or unclear reasons. Prognostic factor measurement had moderate risk of bias and moderate attrition and selection bias. Outcome measurement had high risk of bias as it relied on telephone interviews. The study had moderate risk of confounding due to small sample size, but key variables were included. There was a moderate risk of bias in self-fulfilling prophecy. |
| Ducci | 2017 | 29125072 |  | Continuous variable | 90-d late dependency mRS between 3-5 | 99 | Dependency in n=39 (39.4%) | Sensitivity 79.5%, specificity 73.3% p<0.001 | Moderate | The study had moderate risk of bias in participation and high risk of bias in attrition, with approximately 15% attrition due to death or unclear reasons. Prognostic factor measurement had moderate risk of bias and moderate attrition and selection bias. Outcome measurement had high risk of bias as it relied on telephone interviews. The study had moderate risk of confounding due to small sample size, but key variables were included. There was a moderate risk of bias in self-fulfilling prophecy. |
| Lee | 2017 | 29067622 | admission | Continuous variable | 90-d poor outcome = mRS ≥ 3 | 926 | NA | OR 1.23, 95% 1.20-1.26 p<0.001 | Moderate | A study with moderate risk of bias analyzed 926 out of 1390 participants (66.6%). Those who received intra-arterial intervention were excluded. However, 370 patients lacked 3-month outcome data, indicating high attrition. The modified Rankin Scale (mRS) was used to measure outcomes, documented by stroke NP/neurologists, but not all were certified in mRS scoring. |
| Rost | 2020 | 22116811 | admission | Continuous variable | 180-d good outcome (mRS 0-2) | 569 | NA | OR 0.86, 95% CI 0.8-0.96 | Low | Retrospective analysis hampers assessment of residual confounding and timing of stroke symptom onset interaction. |
| Kuwashiro | 2011 | 21992812 | admission | Continuous variable | 90-d poor functional outcome | 241 | NA | AOR 1.22, 95% CI 1.12 to 1.35, p<0.001, per 1-score increase | Low | This study only included patients with first time ischemic stroke and who also had DM |

**PREVIOUS STROKE**

| **First author last name** | **Year** | **PMID** | **Timing of prognostic factor evaluation** | **Prevalence % of the predictor** | **Outcome(s)** | **Sample size for the outcome** | **Prevalence % of the outcome** | **Effect size** | **Overall risk of bias** | **Overall risk of bias: comments** |
| --- | --- | --- | --- | --- | --- | --- | --- | --- | --- | --- |
| Shi | 2016 |  | On admission | 13.3% | 1-y death/dependency = mRS 3-6 | 2168 | NA | AOR 2.08 (95% CI 1.51-2.87) | Moderate | The study included AIS patients within 14 days of symptom onset and had a complete 12-month follow-up. However, there was no data on loss to follow-up or imputation, and no information was provided on WLST or patient deaths. |
| Sumer | 2003 | 12675701 | On admission | 13.9% | 6-m mRS | 266 | Prior stroke  mRS 1-2 = 20 patients (11%) mRS 3-6 = 17 patients (19%) | p=0.09 | Moderate | Study attrition- 54 patients not included because follow-up was incomplete. 34 patients died by six months; outcome measurement- moderate ROB, does not say how the mRS scores were evaluated at six months |
| Demchuk | 2001 | 11502916 | On admission | 13.2% | good outcome = mRS <2 | 616 | NA |  | High | Large proportion of eligible patients were excluded from study. NIHSS and mRS evaluation didn't seem super valid/reliable. |
| Kammersgaard | 2005 | 21852614 | On admission | 21.6% | 5-y mortality | 869 | 71.9% | HR 1.4, 95% CI 1.1-1.7  p<0.001 | Low | study attrition- only 6 patients lost due to moving out of the country; outcome measurement- all causes of death attainable through Danish Central Registry; |
| Cucchiara | 2004 | 14707586 | On admission | 19% | mortality | 564 | alive at 30 days (n=451) previous TIA 19% dead at 30 days (n=113) previous TIA 20% | p=0.72 | Low | Study attrition- moderate; study confounding- moderate; self-fulfilling prophecy- treatment was never started for 15 patients. |
| Cucchiara | 2004 | 14707586 | On admission | 22% | mortality | 564 | alive at 30 days (n=451) previous stroke 22% dead at 30 days (n=113) previous stroke 22% | p=0.89 | Low | Study attrition- moderate; study confounding- moderate; self-fulfilling prophecy- treatment was never started for 15 patients. |
| Jorgenson | 2020 | 10512899 | On admission | 25% | good functional outcome = BI ≥ 50 points | 84 | good outcome 24% poor outcome 25% | p=0.45 | Low | Well-designed study with minimal risk of bias |
| Henon | 1995 | 7886712 | On admission | 11% | 3-mo functional outcome (Glasgow Outcome Scale) score 1-2 | 152 | NA | OR 4.06, 95% CI 1.40-11.75 | moderate | The study had issues with participation, attrition, and outcome measurement. A high cutoff point for preexisting dementia excluded certain patients. There was a significant drop in participation at each time point, with only 58.6% remaining at month 24. Evaluating cognitive status through telephone contact with patient family or practitioners raised concerns about validity. |
| Tatemichi | 1993 | 8498836 | On admission | NA | 3 mo post stroke dementia | NA | Non demented, n=185  20.0% with prior stroke  demented n=66  17.2% with prior stroke  p=0.064 | B=0.9976, SE=0.3943  OR 2.7, 95% CI 1.3-3.9 | Low | Outcome measurement- follow-up performed in-person at outpatient clinic or by medical personnel at patient's home |
| Timsit | 2016 | 31008289 | On admission | 13.59% | 28-d late mortality | 3024 | overall mortality was 38.49% at 60mo | AHR = 1.08, 0.88-1.33, p=0.4694 | Low | Study participation- early mortality occurred in 313 (9.3%) of patients during the first 28d; |
| Toon | 2017 | 28178407 | On admission | NA | 90-d mortality | 335 | good outcome achieved in 151/335 (45.1%) of patients | AOR 3.124, 95% CI 1.340-7.281 p=0.008 | Low | Study attrition: n=2 lost to follow-up. Outcome measurement: mRS assessed by stroke neurologist at 3-month outpatient visit, or via telephone interview if in-person assessment was not possible. |
| Li | 2019 | 30396839 | On admission | 23.5% | 90-d mRS good = mRS 0-2 poor = mRS 3-6 | 685 | Good outcome (n=445) n=92 (20.7%)  poor outcome (n=240) n=69 (28.8%) | OR 1.79 (1.14-2.81), P=0.012 | High | Study group lost to follow-up (10.1%), imputation used for 90-day mRS, validity unknown. |
| Lee | 2017 | 29067622 | On admission | 23.5% | 90-d poor outcome = mRS ≥ 3 | 926 | NA | OR 1.23, 95% 1.20-1.26 p<0.001 | High | Study had issues with participation, attrition, and outcome measurement. Despite these, it was a large study with interesting data, so it may be worth verifying the findings. |
| Akhtar | 2019 | 30545720 | On admission | 12.70% | 90-d mRS 3-6 | 2961 | 24.00% | OR: 1.765 (1.205 2.585), p=0.004 | Moderate | Prospective study, 619 patients |
| Gory | 2018 | 29393092 | On admission | 29.17% | 90-d mortality | 117 | 41.90% | OR: 3.78 (1.29–14.53) p=0.024 | Moderate | 152 patients, prospective, 2016-2017 |
| Laible | 2017 | 29084408 | On admission | 19.40% | 90-d mRS ≥ 3 | 505 | 66.52% | OR: (1.28 0.70–2.34) p=0.429 | Moderate | Prospective, 2010-2016, 505 patients |
| Li | 2019 | 30396839 | On admission | 23.50% | 90-d mRS 3-6 | 685 | 35.04% | OR: 1.79 (1.14-2.81) p=.012 | Moderate | Retrospective study, 685 participants, 2013-2025 |
| Li | 2019 | 30396839 | On admission | 23.50% | AF | 685 | 29.80% | NA | Moderate | Retrospective study, 685 participants, 2013-2039 |
| Wei | 2010 | 20651267 | On admission | 29% | 1-y mRS | 4782 | 29.97% | OR: 1.68 (1.44–1.97) | Moderate | Prospective, 4782 participants, 2010 |
| Kissela | 2009 | 19109548 | On admission | 28.80% | 4-year functional outcome (mRS) | 451 | NA | OR 0.58 (0.18 – 0.98, P=0.005 | High | The study revealed puzzling results, such as smoking and high cholesterol appearing beneficial, possibly due to cohort characteristics or survival bias. The models developed have limitations, applying only to hospitalized stroke patients who survive the initial days, with biased data collection and non-uniform follow-up, emphasizing the need for ongoing testing and validation. |
| Wahlgren | 2008 | 18927461 | On admission | 9% | 90-d mRS 0-2 | 6947 | 50% | OR: 0.80 (0.65-0.98) | Moderate | 6947 participants, review of RCT, 2002-2006 |
| Wahlgren | 2008 | 18927461 | On admission | 12% | 90-d mRS 0-2 | 6947 | 50% | OR: 0.75 (0.62 - 0.91) | Moderate | 6947 participants, review of RCT, 2002-2006 |
| Demeestere | 2018 | 30355098 | On admission | 10.30% | 90-d mRS 0-2 | 156 | 58.9% | OR 1.28 (0.33-5.01); p=0.72 | High | 156 patients, retrospectively analyzed, but prospectively enrolled in the CRISP study evaluating LVO in the anterior circulation who underwent endovascular therapy and had successful reperfusion |
| Kim | 2014 | 24719133 | On admission | 17.1% | 3-y mortality | 2820 | 17.7% | p<0.001 | Moderate | 2820 patients, 344 with early neurologic deterioration, which was the primary analysis; 200-2008 single hospital, excluding patients with recurrent stroke, alteplase receipt, and late >3 day presentation |

REVASCULARIZATION STATUS

| **First author last name** | **Year** | **PMID** | **Timing of prognostic factor evaluation** | **Prevalence % of the predictor** | **Outcome(s)** | **Sample size for the outcome** | **Prevalence % of the outcome** | **Effect size** | **Overall risk of bias** | **Overall risk of bias: comments** |
| --- | --- | --- | --- | --- | --- | --- | --- | --- | --- | --- |
| Toon | 2017 | 28178407 | After thrombolysis/thrombectomy | 81.8% | 90-d mRS 0-2 | 335 | 45.1% | AOR 4.658, 95% CI 2.240-9.689, p<0.001 | Low | Study attrition: 2 participants were lost to follow-up. Outcome measurement: The Modified Rankin Scale (mRS) was assessed by a stroke neurologist during the 3-month outpatient visit. If an in-person assessment was not possible, it was conducted through a telephone interview. |
| Park | 2020 | 32323503 | After thrombolysis/thrombectomy | NA | 90-d mRS 0-2 | 136 | 25.7% | AOR 5.636, 95% CI 1.216-26.119 | Low | Recanalization status was assessed by two blinded investigators. The interrater agreement, measured by kappa values, was high at 0.875, 0.813, and 0.905. |
| Pikija | 2018 | 30353493 | After thrombolysis/thrombectomy | 75% | 90-d mRS 0-2 | 174 | 50% | OR 5.12, 95% CI 1.01-25.80, p=0.015 | Low | The study had limitations, including its retrospective design, LDL-C was major predictor evaluated. The demographics, stroke severity, and outcomes were comparable to large EVT trials, with similar rates of favorable outcomes and mortality. |
| Potreck | 2019 | 30887195 | After thrombolysis/thrombectomy | 67% | 90-d mRS 0-2 | 131 | 86% | OR 5.54, 1.80-17.02, p=0.003 | Low | Study attrition- retrospective study, n=156. 25 excluded due to image quality. These patients could have had more severe/complicated cases or just due to other reasons. ; outcome measurement- image quality was evaluated blindly, excluding 25 patients |
| Broocks | 2019 | 31667625 | After thrombolysis/thrombectomy | 77% | 90-d mRS 0-2 | 178 | 93 | OR 2.19, 1.14-69.75, p=0.040 | High | Prognostic factors were measured through blinded image analysis, and functional outcomes after 90 days were determined using mRS scores from an undisclosed registry. |
| Wollenweber | 2019 | 31337298 | After thrombolysis/thrombectomy | 83% | 90-d mRS 0-2 | 2637 | NA | OR 1.69, 95% CI 1.45-1.96 | Moderate | Absence of external on-site monitoring and a central imaging core laboratory. Data underwent central quality checks, and centers with unresolved queries were excluded. Complete coverage of all EVT procedures and the total number of procedures in Germany is unknown, but the study's large overall sample size reduces the likelihood of systematic inclusion bias. |
| Kaesmacher | 2019 | 30827193 | After thrombolysis/thrombectomy | 69.9% | 90-d mRS 0-3 | 237 | 40.1% | AOR 5.534, 95% CI 2.363-12.961 | Low | Several biases exist in the study. ASPECTS scores were rated at each center without core-lab adjudication, leading to uncertainty due to interrater variability. The low ASPECTS group was primarily represented by a few centers, limiting generalizability. The ASPECTS scale failed to account for ischemia outside the middle cerebral artery territory, and the inclusion of only endovascularly treated patients introduced a selection bias. Other biases included operator-graded TICI scoring and a potential selection bias due to a 10.1% lost-to-follow-up rate. |
| Kaesmacher | 2019 | 30827193 | After thrombolysis/thrombectomy | 69.9% | mortality | 237 | 40.9% | AOR 0.180, 95% CI 0.083-0.390 |  | Several biases exist in the study. ASPECTS scores were rated at each center without core-lab adjudication, leading to uncertainty due to interrater variability. The low ASPECTS group was primarily represented by a few centers, limiting generalizability. The ASPECTS scale failed to account for ischemia outside the middle cerebral artery territory, and the inclusion of only endovascularly treated patients introduced a selection bias. Other biases included operator-graded TICI scoring and a potential selection bias due to a 10.1% lost-to-follow-up rate. |
| Kaesmacher | 2019 | 30827193 | After thrombolysis/thrombectomy | NA | NIHSS improvement (on admission and after 24h) | 237 | NA | p<0.001 | Low | Several biases exist in the study. ASPECTS scores were rated at each center without core-lab adjudication, leading to uncertainty due to interrater variability. The low ASPECTS group was primarily represented by a few centers, limiting generalizability. The ASPECTS scale failed to account for ischemia outside the middle cerebral artery territory, and the inclusion of only endovascularly treated patients introduced a selection bias. Other biases included operator-graded TICI scoring and a potential selection bias due to a 10.1% lost-to-follow-up rate. |
| Yoo | 2020 | 22426317 | After thrombolysis/thrombectomy | 72.9% | 90-d mRS 0-2 | 107 | NA | R=-0.512, P=0.0006 | High | Exclusion of approximately 25% of patients due to inadequate imaging, but there were no significant differences in major clinical variables between included and excluded patients, except for congestive heart failure. The optimal time window for endovascular therapy remains uncertain, |
| Galimanis | 2011 | 22363057 | After thrombolysis/thrombectomy | 70.3% | 90-d mRS 0-2 | 623 | 48.9% | OR 4.13 (2.55-6.6), P<0.0001 | Low | The study had a high follow-up rate of 98.6% at 3 months. Follow-ups included both clinical exams and telephone interviews, although specific details regarding the distribution between these two methods were not provided. |
| Galimanis | 2011 | 22363057 | After thrombolysis/thrombectomy | 70.3% | 90-d survival | 623 | NA | OR 1.78 (1.08-2.9), P=0.023 | Low | The study had a high follow-up rate of 98.6% at 3 months. Follow-ups included both clinical exams and telephone interviews, although specific details regarding the distribution between these two methods were not provided. |
| Yeo | 2013 | 23599933 | After thrombolysis/thrombectomy | NA | 90-d mRS 0-1 | NA | NA | OR: 3.048, 95% CI: 1.537-6.046; P = 0.001 | Low | Limited arterial recanalization data, taking wither within 2 or at 24 hours, excluding potential recanalization in between. Recanalization-reocclusion-recanalization phenomenon may impact outcomes. Missing data. |
| Yeo | 2013 | 23599933 | After thrombolysis/thrombectomy | NA | 90-d mRS 0-1 | NA | NA | OR: 4.329, 95% CI: 2.382-9.974; P =0.001 | Low | Limited arterial recanalization data, taking wither within 2 or at 24 hours, excluding potential recanalization in between. Recanalization-reocclusion-recanalization phenomenon may impact outcomes. Missing data. |
| Wang | 2019 | 30539756 | After thrombolysis/thrombectomy | 61.68% | 90-d mortality | 321 | 19.63% | NA | Moderate | 321 participants, retrospective study |
| Zhang | 2018 | 29053905 | After thrombolysis/thrombectomy | 62.03% | 90-d mRS 0-2 | 158 | 29.70% | OR: 10.358 (3.113–34.468) p<0.001 | Moderate | 158 participants, retrospective analysis, 2009-2016 |
| Zhang | 2018 | 29053905 | After thrombolysis/thrombectomy | 62.03% | 90-d mRS 0-2 | 158 | 29.70% | OR: 34.088 (2.812–413.223) p=0.006 | Moderate | 158 participants, retrospective analysis, 2009-2016 |
| Kimura | 2011 | 21397255 | After thrombolysis/thrombectomy | 73.50% | Ischemic lesion volume < 40 | 110 | 44.55% | OR: 6.8 (2.2–21.1) p=0.0009 | Moderate | 110 participants, prospective study, 2006-2010 |
| Kimura | 2011 | 21397255 | After thrombolysis/thrombectomy | 73.50% | Ischemic lesion volume < 80 | 110 | 55.45% | OR: 8.4 (2.4–29.9) p=0.001 | Moderate | 110 participants, prospective study, 2006-2010 |
| Nogueira | 2009 | 19875740 | After thrombolysis/thrombectomy | 100% | 90-d mRS 0-2 | 290 | 32.40% | OR: 20.43 (7.74 –53.92) p<0.0001 | Moderate | Secondary analysis of MERCI trial, 305 participants |
| Al-Ajlan | 2018 | 29170266 | After thrombolysis/thrombectomy | 100% | 90-d mRS 0-2 | 206 | 35.9% | p=0.02 | Moderate | 206 prospectively enrolled patients in the REVASCAT trial; 103 received mechanical thrombectomy for anterior circulation, LVO |
| Kim | 2017 | 28176499 | After thrombolysis/thrombectomy | 88.5% | 90-d mRS 0-2 | 104 | 44% | p=0.03 | Moderate | 104 patients from a prospective registry of 5558 patients, with endovascular recanalization treatment for LVO |
| Zhang | 2014 | 24994827 | After thrombolysis/thrombectomy | 19.00% | 90-d mRS >2 | 129 | 57% | OR 3.72 (1.10–12.53); p=0.034 | Moderate | 129 consecutive, prospectively enrolled patients with stroke undergoing endovascular therapy at a single hospital from 2006-2013 |
| Ricciardi | 2014 | 24851928 | After thrombolysis/thrombectomy | 31.5% | 90-d mRS 0-2 | 159 | 46.5% | OR 6.02 (1.10-32.85); p=0.038 | Moderate | 159 patients in a prospective registry at a single hospital with LVO, presenting within 12 hours of onset and evaluated using the IPAQ assessing pre-stroke physical activity |

ASTRAL

| **FIRST AUTHOR LAST NAME** | **YEAR OF PUBLICATION** | **PMID** | **OUTCOME(S)** | **Sample size for the outcome of interest** | **Number of patients with the outcome of interest** | **DISCRIMINATION: C-statistic/ Area under the curve with 95% CI** | **CALIBRATION: Predicted to observed, Hosmer-Lemeshow or other, or enter "Not reported"** | **Any other reported effect size (Odds Ratio, 95% CI etc)- enter as comment** | **Development vs Validation of model** | **OVERALL RISK OF BIAS FOR THE STUDY** | **Comments: Overall Risk of Bias** | **OVERALL CONCERN ABOUT APPLICABILITY** |
| --- | --- | --- | --- | --- | --- | --- | --- | --- | --- | --- | --- | --- |
| Ntaios | 2012 | 22649218 | 90-d mRS >2 | 1645 | 559 | 0.85 (0.82-0.88) | Calibration plot | NA | Development | moderate | ROB- Predictors. Self-fulfilling prophecy Applicability- participants. General IS population, not specific for NCC patients | moderate |
| Ntaios | 2012 | 22649218 | 90-d mRS >2 | 1659 | Not reported | 0.94 (0.91-0.97) | Calibration plot | NA | Validation | moderate | ROB- Predictors. Self-fulfilling prophecy Applicability- participants. General IS population, not specific for NCC patients | moderate |
| Ntaios | 2012 | 22649218 | 90-d mRS >2 | 653 | Not reported | 0.77 (0.72-0.82) | Calibration plot | NA | Validation | moderate | ROB- Predictors. Self-fulfilling prophecy Applicability- participants. General IS population, not specific for NCC patients | moderate |
| Papavasileio | 2013 | 23559264 | 5-year mRS >2 | 1520 | Not reported | 0.89 (95% CI, 0.88–0.91) | Calibration plot | NA | Validation | high | ROB- Participants, predictors, outcome. Self-fulfilling prophecy Applicability- participants. Single-center design, limited to first-ever stroke patients. Some mRS assessments conducted via telephone | high |
| Papavasileio | 2013 | 23559264 | 5-year Mortality | 1520 | Not reported | 0.81 (95% CI, 0.78–0.83) | Calibration plot | NA | Validation | high | ROB- Participants, predictors, outcome. Self-fulfilling prophecy Applicability- participants. Single-center design, limited to first-ever stroke patients. Some mRS assessments conducted via telephone | high |
| Cooray | 2016 | 27174528 | 90-d mRS >2 | 36 131 | 20740 | 0.79 | Calibration plot | NA | Validation | high | ROB- Participants, predictors. Self-fulfilling prophecy Applicability- participants. IVT population | low |
| Asuzu | 2015 | 26032809 | 90-d mRS >4 | 303 | Not reported | 0.84 | Hosmer-Lemeshow | NA | Validation | high | ROB- Participants, predictors. Self-fulfilling prophecy Applicability- participants. IVT population | high |
| Liu | 2013 | 23493731 | 90-d mRS >2 | 3755 | 1473 | 0.82 | Calibration plot | NA | Validation | high | ROB- Predictors. Self-fulfilling prophecy Applicability- participants. General IS population, not specific for NCC patients | high |
| Quinn | 2017 | 28794250 | 90-d mRS>2 | 5341 | Not reported | 0.79 (0.78 - 0.80) | Hosmer-Lemeshow | NA | Validation | high | ROB- Participants, predictors. Self-fulfilling prophecy Applicability- participants. VISTA Database | unclear |
| Quinn | 2017 | 28794250 | 90-d Mortality | 5501 | Not reported | 0.76 (0.74-0.77) | Hosmer-Lemeshow | NA | Validation | high | ROB- Participants, predictors. Self-fulfilling prophecy Applicability- participants. VISTA Database | unclear |
| Wang | 2017 | 28236594 | 180-d mRS >2 | 323 | 96 | 0.83 | Hosmer-Lemeshow | NA | Validation | high | ROB- Predictors. Self-fulfilling prophecy Applicability- participants. General IS population, not specific for NCC patients | high |
| Wang | 2017 | 28236594 | 180-d Mortality | 323 | 21 | 0.85 | Hosmer-Lemeshow | NA | Validation | high | ROB- Predictors. Self-fulfilling prophecy Applicability- participants. General IS population, not specific for NCC patients | high |
| Ferraz | 2020 | 31858586 | 90-d mRS>2 | 375 | 228 | 0.73 | Not reported | NA | Validation | high | ROB- Participants, predictors. Self-fulfilling prophecy Applicability- participants. IVT-EVT population | high |

Body of evidence: ROB in the following domains: participants, predictors and self-fulfilling prophecy, with concern about applicability in the participants domain.

DRAGON

| **FIRST AUTHOR LAST NAME** | **YEAR OF PUBLICATION** | **PMID** | **OUTCOME(S)** | **Sample size for the outcome of interest** | **Number of patients with the outcome of interest** | **DISCRIMINATION: C-statistic/ Area under the curve with 95% CI** | **CALIBRATION: Predicted to observed, Hosmer-Lemeshow or other, or enter "Not reported"** | **Any other reported effect size (Odds Ratio, 95% CI etc)- enter as comment** | **Development vs Validation of model** | **OVERALL RISK OF BIAS FOR THE STUDY** | **Comments: Overall Risk of Bias** | **OVERALL CONCERN ABOUT APPLICABILITY** |
| --- | --- | --- | --- | --- | --- | --- | --- | --- | --- | --- | --- | --- |
| Strbian | 2012 | 22311929 | 90-d mRS>2 | 1319 | 521 | 0.84 | NA | NA | Both | moderate | ROB- Participants, predictors. Self-fulfilling prophecy Applicability- participants. IVT population | moderate |
| Strbian | 2013 | 23929752 | 90-d mRS>2 | 4519 | 2543 | 0.84 | Hosmer-Lemeshow | NA | Validation | moderate | ROB- Participants, predictors. Self-fulfilling prophecy Applicability- participants. IVT population | moderate |
| Turc | 2013 | 23482603 | 90-d mRS>2 | 228 | 98 | 0.83 (95% CI 0.78–0.88) | NA | NA | Validation | high | ROB- Participants, predictors. Self-fulfilling prophecy Applicability- participants. The study's monocenter design, adapted score, and limitations in reaching significance for certain parameters in multivariate analysis raise concerns. Applicability to posterior circulation stroke patients is uncertain | high |
| Turc | 2014 | 24896827 | 90-d mRS>2 | 230 | 78 | 0.81 (95%CI 0.75-0.87) | Hosmer-Lemeshow | NA | Validation | high | ROB- Participants, predictors. Self-fulfilling prophecy Applicability- participants. The study's monocenter design, adapted score, and limitations in reaching significance for certain parameters in multivariate analysis raise concerns. Applicability to posterior circulation stroke patients is uncertain | high |
| Pan | 2018 | 29484275 | 90-d mRS>2 | 1128 | 462 | 0.73 | NA | NA | Validation | high | ROB- Participants, predictors. Self-fulfilling prophecy Applicability- participants. IVT population | high |
| Pan | 2018 | 29484275 | 90-d Mortality | 1128 | 115 | 0.74 | NA | NA | Validation | high | ROB- Participants, predictors. Self-fulfilling prophecy Applicability- participants. IVT population | high |
| Cooray | 2016 | 27174528 | 90-d mRS >2 | 33716 | 20740 | 0.77 | Calibration plot | NA | Validation | high | ROB- Participants, predictors. Self-fulfilling prophecy Applicability- participants. IVT population | high |
| Asuzu | 2015 | 26032809 | 90-d mRS >4 | 303 | Not reported | 0.78 | Hosmer-Lemeshow | NA | Validation | high | ROB- Participants, predictors. Self-fulfilling prophecy Applicability- participants. IVT population | high |
| Giralt-Steinhauer | 2013 | 24029547 | 90-d mRS >2 | 297 | 143 | 0.84 | Hosmer-Lemeshow | NA | Validation | high | ROB- Participants, predictors. Self-fulfilling prophecy Applicability- participants. Geriatric IVT population | high |
| Van Hoff | 2014 | 25171393 | 90-d mRS >2 | 169 | 73 | 0.82 | Not reported | NA | Validation | high | ROB- Participants, predictors. Self-fulfilling prophecy Applicability- participants. IVT population | high |
| Zhang | 2015 | 26028300 | 90-d mRS >2 | 970 | 407 | 0.75 (0.70-0.79) | Not reported | NA | Validation | high | ROB- Participants, predictors. Self-fulfilling prophecy Applicability- participants. The study acknowledges selection bias due to a small sample size and low thrombolysis rate. The focus on urban hospitals raises concerns about the generalizability of the findings to a broader population in China. | high |
| Zhang | 2015 | 26028300 | 90-d mRS 0-2 | 970 | 563 | 0.73 (0.70-0.76) | Not reported | NA | Validation | high | ROB- Participants, predictors. Self-fulfilling prophecy Applicability- participants. The study acknowledges selection bias due to a small sample size and low thrombolysis rate. The focus on urban hospitals raises concerns about the generalizability of the findings to a broader population in China. | high |
| Baek | 2015 | 25576347 | 90-d mRS >2 | 120 | 78 | 0.85 | Not reported | NA | Validation | high | ROB- Participants, predictors. Self-fulfilling prophecy Applicability- participants. | high |

Body of evidence: ROB in the following domains: participants, predictors and self-fulfilling prophecy, with concern about applicability in the participants domain.

iSCORE

| FIRST AUTHOR LAST NAME | YEAR OF PUBLICATION | PMID | OUTCOME(S) | Sample size for the outcome of interest | Number of patients with the outcome of interest | DISCRIMINATION: C-statistic/ Area under the curve with 95% CI | CALIBRATION: Predicted to observed, Hosmer-Lemeshow or other, or enter "Not reported" | Any other reported effect size (Odds Ratio, 95% CI, etc)- enter as comment | Development vs Validation of model | OVERALL RISK OF BIAS FOR THE STUDY | Comments: Overall Risk of Bias | OVERALL CONCERN ABOUT APPLICABILITY |
| --- | --- | --- | --- | --- | --- | --- | --- | --- | --- | --- | --- | --- |
| **Saposnik** | 2013 | 23887844 | 90 d mortality | 7140 | 1112 | NA | NA | iScore≥200: OR 5.18; 95%CI 4.37-6.15) | NA | high | ROB- Participants, predictors. Self-fulfilling prophecy Applicability- participants. Small sample size, potential underestimation of the iScore due to unreported renal failure, lack of inclusion of imaging predictors, and data collected from an earlier time period. | high |
| **Saposnik** | 2013 | 23887844 | 90 d mRS <2 | 7140 | 2878 | NA | NA | iScore≥200: OR 0.120; 95% CI 0.092-0.157; p<0.001 | NA | high | ROB- Participants, predictors. Self-fulfilling prophecy Applicability- participants. Small sample size, potential underestimation of the iScore due to unreported renal failure, lack of inclusion of imaging predictors, and data collected from an earlier time period. | high |
| **Saposnik** | 2013 | 23887844 | 90 d BI >90 | 7140 | 3123 | NA | NA | Iscore continuous: OR 0.977; 95%CI 0.976-0.979 | NA | high | ROB- Participants, predictors. Self-fulfilling prophecy Applicability- participants. Small sample size, potential underestimation of the iScore due to unreported renal failure, lack of inclusion of imaging predictors, and data collected from an earlier time period. | high |
| **Saposnik** | 2011 | 21300951 | 30 d mortality | 12, 262 | Derivation cohort: 1004  Internal validation cohort: 509  External validation cohort: 380 | Derivation: 0.850  Internal validation: 0.85  External validation: 0.79 | Hosmer-Lemeshow test | Pearson correlation coefficient, 0.992 | Both | moderate | ROB- Participants, predictors. Self-fulfilling prophecy Applicability- participants. Non-representative patient selection, exclusion of certain variables, limited applicability, and the need for recalibration. | moderate |
| **Saposnik** | 2011 | 21300951 | 1 y mortality | 12, 262 | Derivation cohort: 1853  Internal validation cohort: 924  External validation cohort: 798 | Derivation: 0.823  Internal validation: 0.84  External validation: 0.782 | Hosmer-Lemeshow test | Pearson correlation coefficient, 0.996 | Both | moderate | ROB- Participants, predictors. Self-fulfilling prophecy Applicability- participants. Non-representative patient selection, exclusion of certain variables, limited applicability, and the need for recalibration. | moderate |
| **Nikneshan** | 2013 | 23359359 | Discharge mRS 0–2 | 12,686 | 328 | NA | NA | Significant decrease in the likelihood of a favorable outcome with higher iScores after tPA administration (P < 0.001). | NA | high | ROB- Predictors. Self-fulfilling prophecy Applicability- participants. Primarily focused on diabetes's influence on outcomes using the iScore. Smaller sample sizes in subgroup analysis could lead to a type II error, and despite adjusting for confounding variables, residual confounding is possible. | high |
| **Nikneshan** | 2013 | 23359359 | 30 d mortality | 12,686 | 1559 | NA | NA | Non-significant | NA | high | ROB- Predictors. Self-fulfilling prophecy Applicability- participants. Primarily focused on diabetes's influence on outcomes using the iScore. Smaller sample sizes in subgroup analysis could lead to a type II error, and despite adjusting for confounding variables, residual confounding is possible. | high |
| **Park** | 2013 | 23539526 | 90 days mRS >2 | 4061 | 1496 | 0.819 (0.805-0.833) | Hosmer–Lemeshow test | r=0.990 | Validation | moderate | ROB- Predictors. Self-fulfilling prophecy Applicability- participants. Exclusion of imaging findings limits discrimination power; no treatment-based stratification; small sample size. | moderate |
| **Park** | 2013 | 23539526 | 30 d mortality | 4061 | 294 | 0.861 (0.840-0.883) | Hosmer–Lemeshow test | r=0.969 | Validation | moderate | ROB- Predictors. Self-fulfilling prophecy Applicability- participants. Exclusion of imaging findings limits discrimination power; no treatment-based stratification; small sample size. | moderate |
| **Zhang** | 2013 | 23652267 | 30 d mortality | 11,656 | NA | 0.825 (0.807-0.843) | Hosmer–Lemeshow test | r= 0.925 | Validation | moderate | ROB- Participants, predictors. Self-fulfilling prophecy Applicability- participants. Selection bias (urban hospitals only), NIHSS to CNS conversion, incomplete iScore data (no imaging/laboratory info), limited applicability to hospitalized acute ischemic stroke patients. | moderate |
| **Zhang** | 2013 | 23652267 | 1 y mortality | 11, 051 | NA | 0.822 (0.810-0.833) | Hosmer–Lemeshow test | r=0.998 | Validation | moderate | ROB- Participants, predictors. Self-fulfilling prophecy Applicability- participants. Selection bias (urban hospitals only), NIHSS to CNS conversion, incomplete iScore data (no imaging/laboratory info), limited applicability to hospitalized acute ischemic stroke patients. | moderate |
| **Bejot** | 2013 | 24052070 | 30 d mortality | 1,092 | 8.7% | 0.85 (0.82-0.89) | Hosmer-Lemeshow test | NA | Validation | high | ROB- Participants, predictors. Self-fulfilling prophecy Applicability- participants. Limited sample size and unavailable data undermine iScore's reliability for long-term functional outcomes. Lack of recorded cause of death data impedes accurate assessment of iScore's prediction accuracy for specific mortality | high |
| **Bejot** | 2013 | 24052070 | 1 y mortality | 1,092 | 22.3% | 0.84 (0.81-0.87) | Hosmer-Lemeshow test | NA | Validation | high | ROB- Participants, predictors. Self-fulfilling prophecy Applicability- participants. Limited sample size and unavailable data undermine iScore's reliability for long-term functional outcomes. Lack of recorded cause of death data impedes accurate assessment of iScore's prediction accuracy for specific mortality | high |
| **Park** | 2014 | 23800501 | 90 days mRS >2 | 4760 | NA | 0.813 (0.778-0.848) | Hosmer-Lemeshow test | iScore ≥ 180: RR 2.28 (1.99-2.61) | Validation | moderate | ROB- Predictors. Self-fulfilling prophecy Applicability- participants. Insufficient early imaging and blood pressure data. Inaccuracy in predicting outcomes for patients with extreme iScore values due to a small sample size. | moderate |
| **Park** | 2014 | 23800501 | 90 d mortality | 4760 | NA | 0.820 (0.769-0.872) | Hosmer-Lemeshow test | iScore ≥ 180: RR 5.84 (3.66-9.33) | Validation | moderate | ROB- Predictors. Self-fulfilling prophecy Applicability- participants. Insufficient early imaging and blood pressure data. Inaccuracy in predicting outcomes for patients with extreme iScore values due to a small sample size. | moderate |
| **van Hooff** | 2014 | **25171393** | 90 d mRS 0-1 | 169 | NA | 0.72 (0.63-0.8) | Calibration plots | NA | Validation | high | ROB- Participants, predictors. Self-fulfilling prophecy Applicability- participants. Small sample size, retrospective design, selection bias | high |
| **van Hooff** | 2014 | **25171393** | 90 d mRS 0-2 | 169 | NA | 0.80(0.73-0.87) | Calibration plots | NA | Validation | high | ROB- Participants, predictors. Self-fulfilling prophecy Applicability- participants. Small sample size, retrospective design, selection bias | high |
| **van Hooff** | 2014 | **25171393** | 90 d mRS 5-6 | 169 | NA | 0.86 (0.8-0.92) | Calibration plots | NA | Validation | high | ROB- Participants, predictors. Self-fulfilling prophecy Applicability- participants. Small sample size, retrospective design, selection bias | high |
| **Wang** | 2017 | 28236594 | 180 d mRS 3-6 | 323 | 96 | 0.798 (0.742-0.853) | Hosmer-Lemeshow test | NA | Validation | high | ROB- Participants, predictors. Self-fulfilling prophecy Applicability- participants. Small sample size | high |
| **Wang** | 2017 | 28236594 | 180 d mortality | 323 | 21 | 0.833 (0.733-0.933) | Hosmer-Lemeshow test | NA | Validation | high | ROB- Participants, predictors. Self-fulfilling prophecy Applicability- participants. Small sample size | high |
| **Quinn** | 2017 | 28794250 | 90 d mRS 3-6 | 10,777 | 5551 | 0.68 (0.67–0.70) | Hosmer-Lemeshow tests | NA | Validation | low | ROB- Participants, predictors. Self-fulfilling prophecy Applicability- participants. Selection bias, measurement bias. Limited inclusion criteria and dichotomization of data may distort the findings. The study's sample, consisting of younger and less impaired patients, may not represent the wider population | low |

Body of evidence: ROB in the following domains: participants, predictors and self-fulfilling prophecy, with concern about applicability in the participants domain.

THRIVE

| **FIRST AUTHOR LAST NAME** | **YEAR OF PUBLICATION** | **PMID** | **PROGNOSTIC MODEL(S) EVALUATED** | **OUTCOME(S)** | **Sample size for the outcome of interest** | **Number of patients with the outcome of interest** | **DISCRIMINATION: C-statistic/ Area under the curve with 95% CI** | **CALIBRATION: Predicted to observed, Hosmer-Lemeshow or other, or enter "Not reported"** | **Any other reported effect size (Odds Ratio, 95% CI etc.)- enter as comment** | **Development vs Validation of model** | **OVERALL RISK OF BIAS FOR THE STUDY** | **Comments: Overall Risk of Bias** | **OVERALL CONCERN ABOUT APPLICABILITY** |
| --- | --- | --- | --- | --- | --- | --- | --- | --- | --- | --- | --- | --- | --- |
| Flint | 2010 | 20223889 | THRIVE | 90-d mRS ≤ 2 | 305 | NA | 0.709 | Hosmer-Lemeshow | NA | Development | high | ROB- Participants, predictors. Self-fulfilling prophecy. EVT population | high |
| Flint | 2013 | 24072004 | THRIVE | 90-d mRS >2 | 5724 | NA | 0.76 (no CI) | Hosmer-Lemeshow | NA | Validation | low | ROB- Participants, predictors. Self-fulfilling prophecy. VISTA Database | low |
| Flint | 2013 | 24072004 | THRIVE | 90-d Mortality | 5724 | NA | 0.72 (no CI) | Hosmer-Lemeshow | NA | Validation | low | ROB- Participants, predictors. Self-fulfilling prophecy. VISTA Database | low |
| Kamel | 2013 | 23122722 | THRIVE | 90-d mRS >2 | NA | NA | 0.22(0.18-0.26) | NA | NA | Validation | high | ROB- Participants, predictors. Self-fulfilling prophecy. NINDS - MERCI Database | high |
| Kamel | 2013 | 23122722 | THRIVE | 90-d Mortality | NA | NA | 0.74 (0.69-0.79) | NA | NA | Validation | high | ROB- Participants, predictors. Self-fulfilling prophecy. NINDS - MERCI Database | high |
| Lei | 2014 | 24743443 | THRIVE | 90-d mRS >2 | 3879 | NA | 0.73 / 0.71 NO CI | NA | NA | Validation | moderate | ROB- Predictors. Self-fulfilling prophecy. Non IVT population | low |
| Lei | 2014 | 24743443 | THRIVE | 90-d Mortality | 3879 | NA | 0.71 / 0.76 NO CI | NA | NA | Validation | moderate | ROB- Predictors. Self-fulfilling prophecy. Non IVT population | low |
| Prabhakaran | 2014 | 24942008 | THRIVE | 90-d mRS >2 | 511 | 186 | 0.65 (0.60–0.70) | NA | NA | Validation | high | ROB- Predictors. Self-fulfilling prophecy. EVT population | high |
| Chen | 2015 | 25649799 | THRIVE | 90-d mRS >2 | 1128 | NA | 0.71 (0.68–0.74) | Calibration plot | NA | Validation | high | ROB- Participants, predictors. Self-fulfilling prophecy. IVT population | high |
| Chen | 2015 | 25649799 | THRIVE | 90-d Mortality | 1128 | NA | 0.78 (0.74–0.82) | Calibration plot | NA | Validation | high | ROB- Participants, predictors. Self-fulfilling prophecy. IVT population | high |
| Flint | 2015 | 26045081 | THRIVE-c | 90-d mRS >2 | 6194 | 2911 | 0·786 | NA | NA | Development | moderate | ROB- Predictors. Self-fulfilling prophecy. SITS+VISTA | low |
| Flint | 2015 | 26045081 | THRIVE-c | 90-d mRS >2 | 6013 | 2786 | 0·784 | NA | NA | Validation | moderate | ROB- Predictors. Self-fulfilling prophecy. SITS+VISTA | low |
| Flint | 2015 | 26045081 | THRIVE-c | 90-d mRS 0-2 | 6013 | 2786 | 0·785 | NA | NA | Validation | moderate | ROB- Predictors. Self-fulfilling prophecy. SITS+VISTA | low |
| Pan | 2018 | 29484275 | THRIVE | 90-d mRS>2 | 1128 | 462 | 0.71 (0.68–0.74) | Calibration plot | NA | Validation | high | ROB- Predictors. Self-fulfilling prophecy. TIMS-China | high |
| Pan | 2018 | 29484275 | THRIVE | 90-d Mortality | 1128 | 115 | 0.78 (0.74–0.82) | Calibration plot | NA | Validation | high | ROB- Predictors. Self-fulfilling prophecy. TIMS-China | high |
| Pan | 2018 | 29484275 | THRIVE-c | 90-d mRS>2 | 1128 | 462 | 0.75 (0.72–0.78) | NA | NA | Validation | high | ROB- Predictors. Self-fulfilling prophecy. TIMS-China | high |
| Pan | 2018 | 29484275 | THRIVE-c | 90-d Mortality | 1128 | 115 | 0.81 (0.77–0.85) | NA | NA | Validation | high | ROB- Predictors. Self-fulfilling prophecy. TIMS-China | high |
| Quinn | 2017 | 28794250 | THRIVE | 90-d mRS>2 | 10033 | NA | 0.77 (0.76–0.78) | Hosmer-Lemeshow | NA | Validation | low | ROB- Predictors. Self-fulfilling prophecy. VISTA Database | unclear |
| Quinn | 2017 | 28794250 | THRIVE | 90-d Mortality | 10033 | NA | 0.73 (0.72-0.74) | Hosmer-Lemeshow | NA | Validation | low | ROB- Predictors. Self-fulfilling prophecy. VISTA Database | unclear |
| Kastrup | 2017 | 28556351 | THRIVE | Discharge mRS 0-2 | 546 | NA | 0.72 | NA | NA | Validation | moderate | ROB- Predictors, analysis. Self-fulfilling prophecy. Non-randomized retrospective design, reliance on discharge outcomes, and absence of routine recanalization assessment | moderate |
| Kastrup | 2017 | 28556351 | THRIVE-c | Discharge mRS 0-2 | 546 | NA | 0.77 | NA | NA | Validation | moderate |  | moderate |
| Kastrup | 2017 | 28556351 | THRIVE | Discharge mRS > 2 | 546 | NA | 0.71 | NA | NA | Validation | moderate |  | moderate |
| Kastrup | 2017 | 28556351 | THRIVE-c | Discharge mRS > 2 | 546 | NA | 0.77 | NA | NA | Validation | moderate |  | moderate |
| Kastrup | 2017 | 28556351 | THRIVE | Mortality | 546 | NA | 0.72 | NA | NA | Validation | moderate |  | moderate |
| Kastrup | 2017 | 28556351 | THRIVE-c | Mortality | 546 | NA | 0.78 | NA | NA | Validation | moderate |  | moderate |
| Chen | 2022 | 34971994 | THRIVE | 90 d mRS ≥ 3 or mortality | 858 | NA | AC: 0.685 (95% CI 0.644-0.724)  PC: 0.709 (95% CI 0.647-0.765) | Hosmer–Lemeshow | NA | Validation | moderate | ROB- Participants, predictors. Self-fulfilling prophecy. Single centre, small sample size | moderate |
| Chen | 2022 | 34971994 | THRIVE | 1 y mRS ≥ 3 or mortality | 858 | NA | AC: 0.701 (95% CI 0.660-0.740)  PC: 0.747 (95% CI 0.687-0.800) | Hosmer–Lemeshow | NA | Validation | moderate | ROB- Participants, predictors. Self-fulfilling prophecy. Single centre, small sample size | moderate |
| Flint | 2022 | 35319310 | THRIVE-EVT | 90 d mRS 0-2 | 787 | 366 | 0.72 | Hosmer-Lemeshow | NA | Both | moderate | ROB- Predictors. Self-fulfilling prophecy. VISTA (7 RCTs of EVT) | low |
| de Oliveira | 2023 | 36434859 | THRIVE | 90 d mRS 3-6 | 386 | 218 | 0.77 | NA | NA | Validation | high | ROB- Participants, predictors. Self-fulfilling prophecy. Single centre, small sample size | unclear |
| Flint | 2023 | 35319310 | THRIVE-c | 90 d mRS 0-2 | 1582 | 366 | 0.716 in development  0.727 in validation | Hosmer-Lemeshow | NA | Both | moderate | ROB- Predictors. Self-fulfilling prophecy. Potentially modifiable factors were not considered, important data on collateral status and operator factors were unavailable | moderate |

Body of evidence: ROB in the following domains: participants, predictors and self-fulfilling prophecy.
